# An Inverse Design Workflow for Solvent Mixtures Based on Hansen Solubility Parameters

**DOI:** 10.1021/acs.jcim.6c02092

**Published:** 2026-10-02

**Authors:** Xue Fang, Shaohua Li, Bo Gao, Ella M. Gale, Charl F. J. Faul, Natalie Fey

**Affiliations:** † School of Chemistry, 1980 University of Bristol, Cantock’s Close, Bristol BS8 1TS, U.K.; ‡ Molecular Sciences Research Hub, 4615 Imperial College London, White City Campus, 82 Wood Lane, London W12 0BZ, U.K.; § Department of Chemical Engineering, Imperial College London, South Kensington campus, London SW7 2AZ, U.K.; ∥ School of Physics, HH Wills Physics Laboratory, University of Bristol, Tyndall Avenue, Bristol BS8 1TL, U.K.

## Abstract

We present *SolvPred*, an open-source Python framework for inverse design of solvent mixtures based on Hansen solubility parameters (HSPs). *SolvPred* allows users to control the composition of solvent mixtures while setting essential experimental constraints. As a case study, we applied *SolvPred* to optimize a polyimide condensation as a model reaction, where the hydrogen-bonding HSP term was selectively tuned. *SolvPred* identified seven viable ternary mixtures from an otherwise intractable space of 171,871 possibilities. The best-performing solvent mixture achieved a fivefold improvement in the product yield at a milder temperature when compared with the conventional single-solvent condition. This work establishes *SolvPred* as a practical solution for rational solvent mixture design and experimental planning, as well as an efficient tool for property-guided solvent optimization within data-driven experimental workflows.

## Graphical abstract

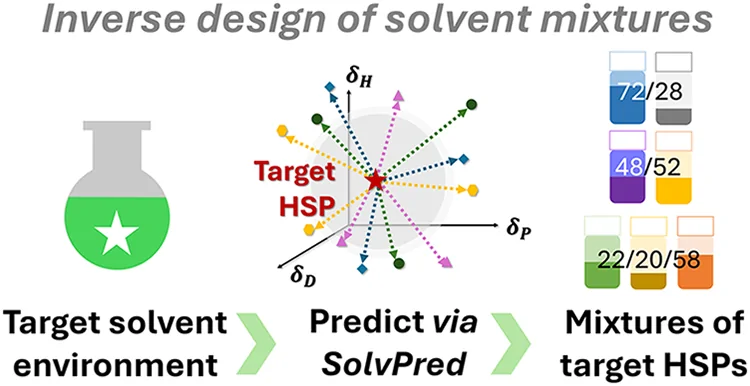

## Introduction

Solvents play a crucial role in chemistry, materials science and the pharmaceutical industry, influencing solubility,^1^ reaction kinetics,^2,3^ and crystallization,^4^ among other processes.^5,6^ To efficiently select solvents, computational tools and algorithms for solvent selection have attracted recent interest. Early milestones included the systematic organization of solvent property space using principal component analysis (PCA) to navigate the high-dimensional solvent space by similarity and variance coverage.^7^ The ACS Green Chemistry Institute also released a solvent selection guide arising from a collaboration of industrial partners, including Pfizer, GSK, and AstraZeneca, *etc.*, ranking solvents based on safety, health and environmental impact, and providing a handy solvent tool for pharmaceutical and green chemistry.^8–10^ More recent progress includes using active learning (AL) for solvent optimization,^11^ natural language processing (NLP) for solvent embedding design,^12^ and multitask machine learning approaches for solvent–solute phase behavior predictions.^13,14^

Despite the importance of solvent selection, navigating the solvent space remains a significant challenge,^15,16^ not just because developing suitable solvent descriptors can be complex,^17^ but also because solvents are categorical variables. Most data-driven approaches, including Design of Experiments (DoE) and Bayesian optimization (BO), are more successful when there is a continuum of options.^18,19^

Unlike continuous variables such as temperature and concentration, categorical variables require specialized encoding or featurization and modelling strategies.^15,16,20–23^ Established approaches for solvent representation, including one-hot encoding,^24^ dimensionality reduction techniques applied to databases of solvent descriptors,^7,25^ graph neural networks^26^ and autoencoders^27^ embed solvents in a numerical latent space. However, these representations are often heavily parameterized, and both the boundaries of the latent space and the rules for mapping from there back to the actual solvents, *i.e.*, selecting the best solvent to have a specific property profile (inverse design), remain uncertain. This “lack of smoothness” renders rigorous interpolation and quantitative comparisons between solvents challenging.^28^

Strategies for inverse design in discrete chemical space include virtual screening *via*, for example, *ab initio* calculations, to compute the target properties of all candidates before selecting the best; global optimization, using for example AL and genetic algorithms to iteratively approach an ideal outcome while selectively maximizing the candidate diversity;^23^ and generative models, via recurrent neural networks and variational autoencoders, to map between the latent space and the design space.^29^ However, virtual screening and global optimization can incur large computing costs and it can be challenging to define the optimization matrices. Here, rationally quantifying the molecular representations and objectives can be bottlenecks.^30^ Generative methods involving neural networks require case-by-case pre-training and fine-tuning of hyperparameters on sufficiently large property datasets, which adds to the complexity of method development. It is also not trivial to define rules for approximating the continuous prediction outcome to the nearest meaningful candidate in a discrete design space. Moreover, experimentally operable conditions, which are typically associated with apparatus, scales or specific experimental procedures, can introduce further divergence from computational models and algorithms, making the reliability of inverse design algorithms less clear when it comes to their application to real-world experiments.

A strategy to improve the continuity of solvent space is to introduce solvent mixtures at different concentration gradients.^28,31^ By varying the ratios of co-solvents, the dataset for solvent-dependent measurables can be systematically expanded, hence building up a foundation that can be used to unravel complex solvent behaviors.^32^ Solvent mixtures can be a useful remedy when single solvents do not allow experimenters to finely control the sensitive solvent–solute interactions.^31,33–36^ Such scenarios, where multiple solutes or functional groups are present in a chemical process where each favors a different microsolvation environment, are commonly anticipated in organic synthesis,^37^ co-crystallization,^38^ and polymer formulation applications.^39^ In addition, solvent mixtures that have overall similar microscopic properties (*e.g.*, polarity) as a single solvent, but differ in macroscopic properties (*e.g.*, toxicity, volatility, pH) can serve as a replacement of the corresponding single solvent.^40^ Because of these advantages, solvent mixtures have been applied to synthetic,^41,42^ analytical,^33,43–45^ material^46–48^ and green chemistry.^40^

However, designed solvent mixtures can introduce substantial combinatorial complexity: for a laboratory holding just 10 common solvents, binary mixtures, varying the composition in 10% increments and assuming full miscibility, can lead to 415 possible combinations. This number grows to 4735 possible options when ternary mixtures are considered. Other practical constraints, such as miscibility, boiling points, thermal expansion effects, and the precision of volumetric mixing, can further complicate the design of solvent mixtures.

In practice, identifying a promising solvent mixture for target applications is highly context-dependent and typically relies on exhaustive trial-and-error screening. This can raise concerns about resource consumption, environmental impact and sustainability. As a result, most reported applications of solvent mixtures focus on binary systems, with considerably fewer studies dedicated to ternary solvent optimization.^35,49–53^

Often in experimental research, solvents are considered less of a priority than other variables of interest and they are selected either from experience or modified from literature suggestions. A good-enough outcome using a single solvent or simple binary mixtures can halt the optimization and claim the “most suitable” conditions for the target application, even if a complex mixture may outperform single solvents at milder conditions. This has left a vast space out of the experimental design scope and can miss potentially interesting results affected by solvents. An efficient inverse design framework to suggest solvent mixtures is therefore needed.

Current efforts in solvent mixture selection fall into two categories: constructing large-scale, standardized datasets that combine computational, literature-mined and experimental data to serve as the foundation for machine learning;^26,54,55^ or adopting high-throughput workflows to screen solvent formulations.^56–58^ Both approaches have limitations: the first, as discussed in our earlier work,^17^ can struggle with large solutes or composite materials, for which molecular representations are non-trivial to define; the second may require specialized equipment and is not always transferable to conventional laboratory settings.^57^ In either case, the direct prediction of the composition of a solvent mixture from desirable solvent properties remains challenging.

Hansen solubility parameters (HSPs) offer a simple yet effective framework for quantifying solvent–solute interactions based on the principle of “like-dissolves-like”.^59^ Over the past decade, HSP theory has been increasingly applied in materials chemistry,^5,17,60–64^ including guiding the design of porous liquids,^62^ improving carbon capture efficiency of porous organic polymers (POPs),^5,17,63^ and controlling polymerization outcomes.^5,17,63,65^ Molecular dynamics studies have revealed that varying HSPs can alter reactant compatibility, leading to different degrees of phase separation that affects the polymerization progress.^65^ Matching HSPs between solvents and polymers has also been experimentally proven as a key factor to promote the formation of extended polymer networks instead of oligomers.^5,63^

HSPs decompose the overall solvent–solute interaction into three orthogonal components: dispersion (δ_*D*_/MPa^1/2^), dipolar effects (δ_*P*_/MPa^1/2^), and hydrogen-bonding (δ_*H*_/MPa^1/2^) interactions.^59,66,67^ These components together define the Hansen space, in which the distance between two species reflects their thermodynamic compatibility, which can serve as a proxy for solubility. Selectively tuning a single HSP dimension directly influences the corresponding interaction type, providing a mechanistic basis for rationalizing solvent effects and enabling systematic solvent optimization.

Beyond single solvents, HSP theory can be extended to solvent mixtures,^68^ where the HSPs of a mixture are defined as a linear combination of component solvents (eq 1)^59,69^

1 $$\delta_{\mathrm{blend}}=\Sigma_{i=1}^{n}c_{i}\delta^{\left(i\right)}$$

where **δ**_**blend**_ are the HSPs of a solvent mixture that consists of *n* different solvents. Each solvent, *i*, has an individual HSP vector **δ** ^(***i***)^, with a volume fraction *c_i_*.

Using this theory enables the construction of a continuous solvent property space, filling the gap between discrete single solvents by using a concentration gradient for a binary (or higher) mixture, hence allowing solvents to be treated as a quasi-continuous variable (Figure 1) in an optimization of experimental conditions.

**Figure 1:**
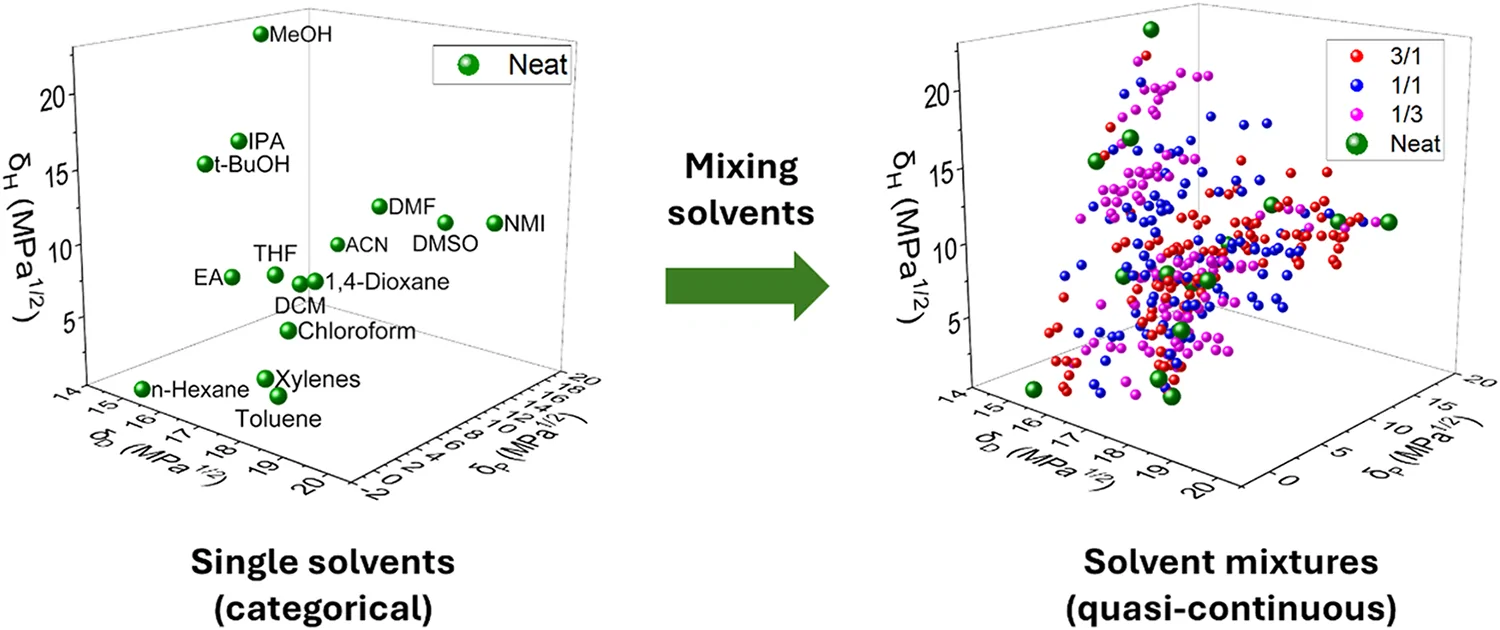
An example demonstrates that solvent mixtures access a substantially larger region of Hansen space compared with single solvents. Under HSP theory, solvents transition from categorical variables in the single solvent case (left) to quasi-continuous variables as solvent mixtures (right), generated by mixing any two neat solvents (green) at 3/1 (red), 1/1 (blue), 1/3 (pink) volumetric ratios.

Based on the linear mixing assumption of eq 1, the mapping between target HSPs and single solvent components can, in principle, be inverted. However, determining a chemically meaningful and experimentally practical composition to achieve an arbitrary target set of HSP values in Hansen space is not straightforward (Figure 2).

**Figure 2:**
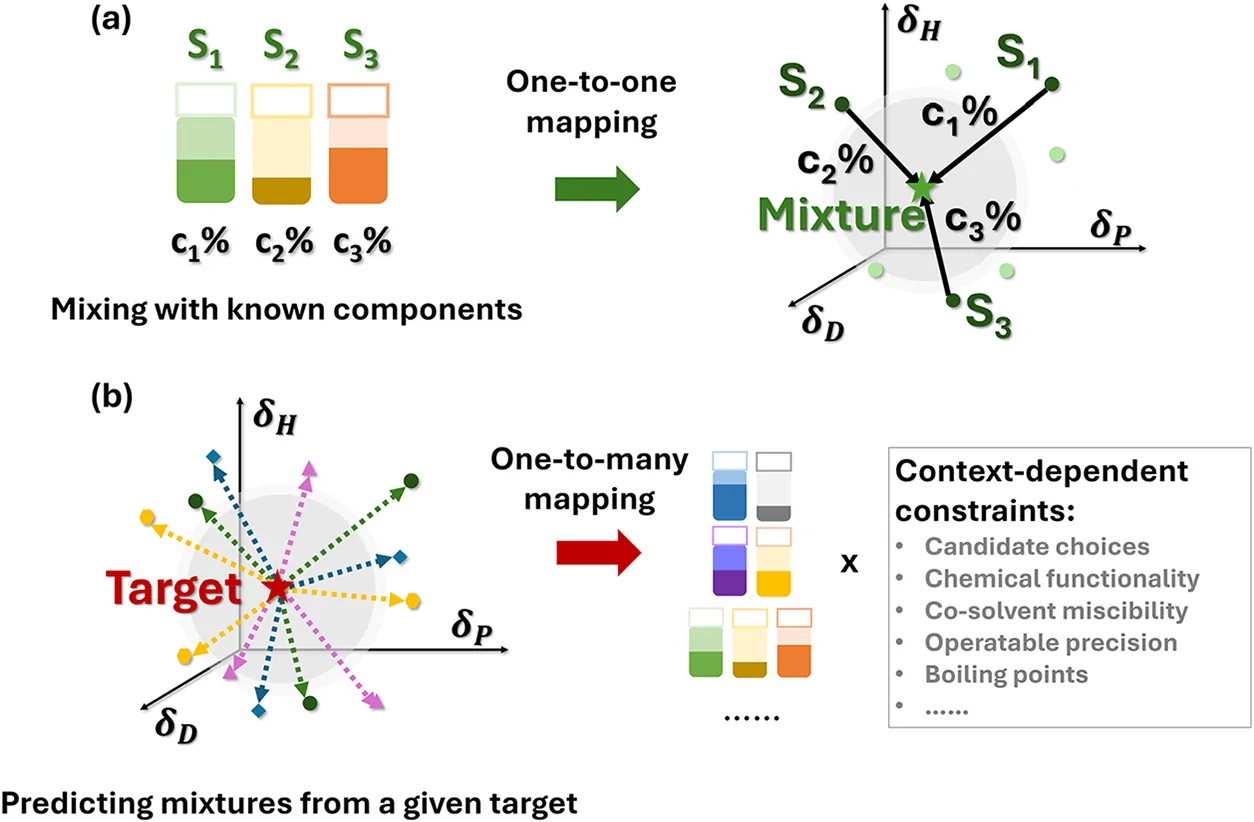
Comparison of forward calculation of solvent mixture HSPs and inverse calculation of mixture components. The forward calculation, (a), with all solvent components (*S*_1_, *S*_2_, *S*_3_) and solvent composition (*c*_1_%, *c*_2_%, *c*_3_%) clearly defined, would yield uniquely determined mixture HSPs as a one-to-one mapping, whereas the inverse calculation, (b), *i.e.*, to deconvolute an arbitrary target HSP into solvent components, would yield one-to-many mapping, with no unique solution and relying on practical constraints.

Predicting the HSPs for a known mixture requires one-to-one mapping (Figure 2a), whereas the inverse is a one-to-many mapping challenge (Figure 2b). In the inverse design scenario, several solutions are possible, depending not only on the choice of solvent candidates, but also on the volume precision with which solvent can be measured and mixed. This may depend on experimental capability, along with other physical property constraints related to application scenarios, including co-solvent miscibility and boiling points. Beyond HSPs, explicit chemical functionalities and safety information on solvents are also important, especially in reaction optimization, when a solvent may co-catalyze a reaction or compete with a reactant.

Here, we introduce an inverse solvent design program, *SolvPred*, for co-solvent prediction from targeted HSPs to address the complexity of solvent mixture research. *SolvPred* allows users to flexibly define the number of co-solvents, volumetric precision, application temperature and solvent candidates. The workflow is associated with an extendible module to filter candidates based on physicochemical constraints and safety requirements.

As a proof-of-concept case study, we applied *SolvPred* to optimize a polyimide condensation reaction, in which the hydrogen-bonding HSP was selectively modified, while dipolar and dispersion interactions were held constant. This approach was motivated by our recent discovery from the HSP database of POPs, where δ_*H*_ was found as the dominant HSP influencing the synthesis outcomes of POPs.^17^ However, the majority of previous reactions were conducted in single solvents. This case study aims to prove the possibility of tuning a specific dimension of HSPs at a well-defined resolution using *SolvPred*, without being overwhelmed by combinatorial complexity.

## Materials and Methods

Details of the theoretical foundations and implementation of the *SolvPred* algorithm are provided in the electronic supporting information (ESI), Section S1. *SolvPred* was developed using Python 3.9.12 and the PyQt6 framework. Theoretical details of the co-solvent prediction algorithm are explained in S1.1 of the SI. Specifically, the construction of *SolvPred* database is described in S1.1.1; the equations and theoretical sources of thermal expansion corrections are provided in S1.1.2; the mathematical details of the inverse design algorithm is introduced in S1.1.3; the perturbation strategy is detailed in S1.1.4; the workflow for indeterminate properties filtering is explained in S1.1.5. The extended solvent candidate filtering modules requires the *RDKit.chem* module,^70^ with details provided in S1.2, where the implementation workflows and working examples for each filtering method are detailed. The filtering method based on descriptive and physical information is described in S1.2.1; the filtering based on chemical information is detailed in S1.2.2; the feature-merging method is explained in S1.2.3. User interfaces are introduced in S1.3, including the toolkit implementation and working examples based on the command-line version (S1.3.1) and the graphical interface version (S1.3.2). The computing performance and sensitivity analysis of the SolvPred algorithm is provided in S1.4.

Experimental details for the case study in this work are provided in S2. Chemicals used in this work include perylene-3,4,9,10-tetracarboxylic dianhydride (PTCDA), melamine, zinc acetate, 1-methylimidazole (NMI), propylene carbonate (PC), *N*-methyl-2-pyrrolidone (NMP), triethylamine (TEA), dimethyl sulfoxide (DMSO), dimethylformamide (DMF), *tert*-butyl alcohol (*t*BuOH) and methanol. All chemicals were purchased from commercial sources and were used as received (S2.1). The model reaction of **pPI-1** was synthesized and characterized according to conditions described in a literature report.^71^ The solvent ternary mixtures used were predicted by *SolvPred*. Details of synthesis and co-solvent predictions for **pPI-1** are provided in S2.2. Characterization of **pPI-1** are provided in S2.3. The method for economic impact evaluation is provided in S2.4.

## Results and Discussion

*SolvPred* aims to predict multi-solvent components from target HSPs with context-dependent requirements. Constraints based on achievable dispensing precision, boiling points and miscibility can be incorporated to allow practical and physically meaningful prediction for experiments. The workflow is summarized in Scheme 1.

**Scheme 1:**
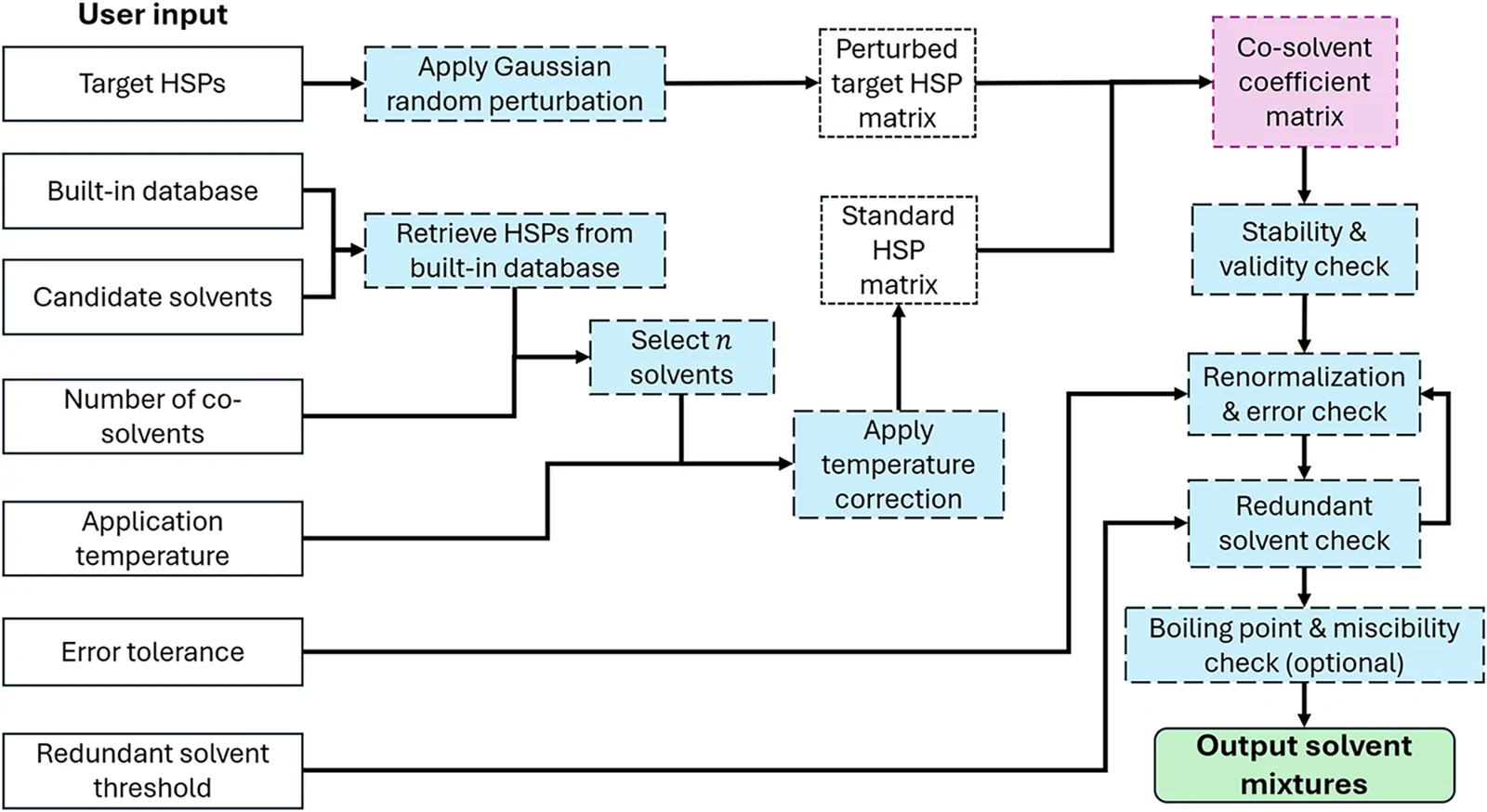
Illustration of the *SolvPred* Workflow

User inputs include the target HSPs, accessible solvent candidates and the temperature. *SolvPred* first retrieves HSPs for each candidate solvent from a built-in database (S1.1.1), followed by applying thermal expansion corrections to δ_*D*_, δ_*P*_, and δ_*H*_ of all candidate solvents (S1.1.2).^59^ The *SolvPred* database was curated from the experimental HSP database of 249 solvents reported in the HSPs user’s guidebook.^59^ To support solvent filtering by properties, this database also includes solvent CAS number, miscibility information, SMILES, and boiling points extracted from *PubChem.* ^72^ Users can specify the desired number of co-solvents. *SolvPred* will construct the corresponding candidate solvent matrix and conduct the co-solvent prediction according to the core algorithm described in the next section. The validity of results were assessed based on experimental constraints.

### SolvPred Core Algorithm

The core algorithm, detailed in S1.1.3, is designed to reverse the forward procedure of calculating blended solvent HSPs (eq 1), by solving for the linear coefficients of a total of *n* co-solvent candidates with target HSPs (eqs 2 and 3). This step aims to calculate the volumetric fractions of each solvent from the target HSPs (**δ**_**target**_, eq S4) and the known HSPs of candidate solvents (**δ**_**cand**_, eqs S5 and S6), by first solving the left pseudoinverse of candidate HSPs (**δ**_**cand**_^–1^) via singular value decomposition. The form of the solvent mixture composition, ***c***, is given in eq S7.

2 $$\delta_{target}=\delta_{cand}c$$

3 $$c=\delta_{cand}^{-1}\delta_{target}$$

To retain physical meaning, two constraints need to be applied (Figure 3). The first is needed to ensure that the total volumetric composition of all co-solvents adds up to 100% (eqs S8 to S12 and Figure 3a). With this constraint, a normalization factor is applied to **δ**_**target**_ and **δ**_**cand**_ to yield the actual target HSPs (eq S9) and the standard HSP matrix (eq S10). The second constraint is necessary to ensure that all concentrations of co-solvents are between 0% to 100% (Figure 3b). The achievable target HSPs are therefore confined to the solvent candidates specified. Specifically, the target HSPs need to be surrounded by the HSP space (convex hull) constructed by all solvent candidates; otherwise, the target is not obtainable without negative concentrations of co-solvents. Before prediction starts, *SolvPred* will calculate the achievable range of target HSPs with respect to candidates and present it to users (Figure 3c), to ensure a feasible combination of solvent candidates and target HSPs is selected. To support the initial selection of candidates, a default candidate list covering common laboratory solvents that are distributed well across the Hansen space, has been provided (Scheme S18). Users can modify the candidate solvents on this basis or create their own list from scratch.

**Figure 3:**
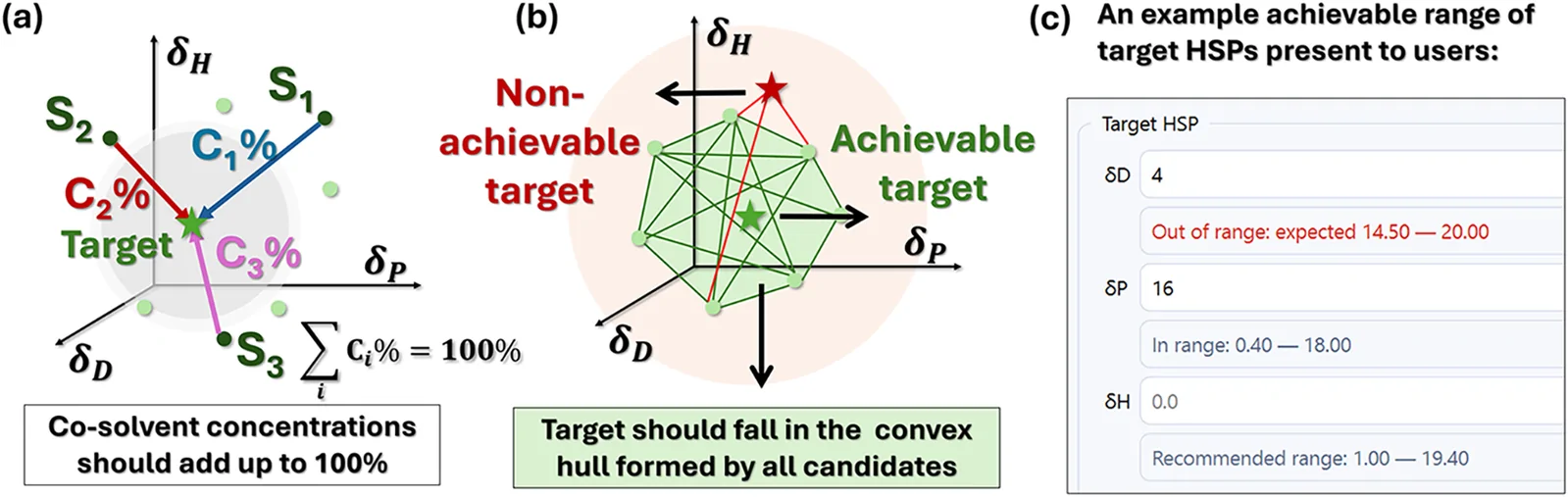
Constraints used in the *SolvPred* core algorithm to ensure physical meaning. (a) Concentrations of all co-solvents should add up to 100%. (b) All co-solvent concentrations should be between 0% to 100%. Target HSPs should be surrounded by the convex hull formed by candidate co-solvents to avoid negative concentration being predicted. An example of the range of target HSPs which is achievable, as presented to users (c).

### Dynamic Precision Control

The directly predicted compositions based on the procedure above face the next problem: the results are of excessively strict numerical precision when compared to what is experimentally practical (Figure 4a). By default, matrix operations using Python in a 64-bit system yield double-precision floating point results at the 10^–15^ level. This numerical resolution, equivalent to a volumetric precision of 10^–13^ %, far exceeds the resolution achievable by common liquid dispensing methods in a wet-lab setting.

**Figure 4:**
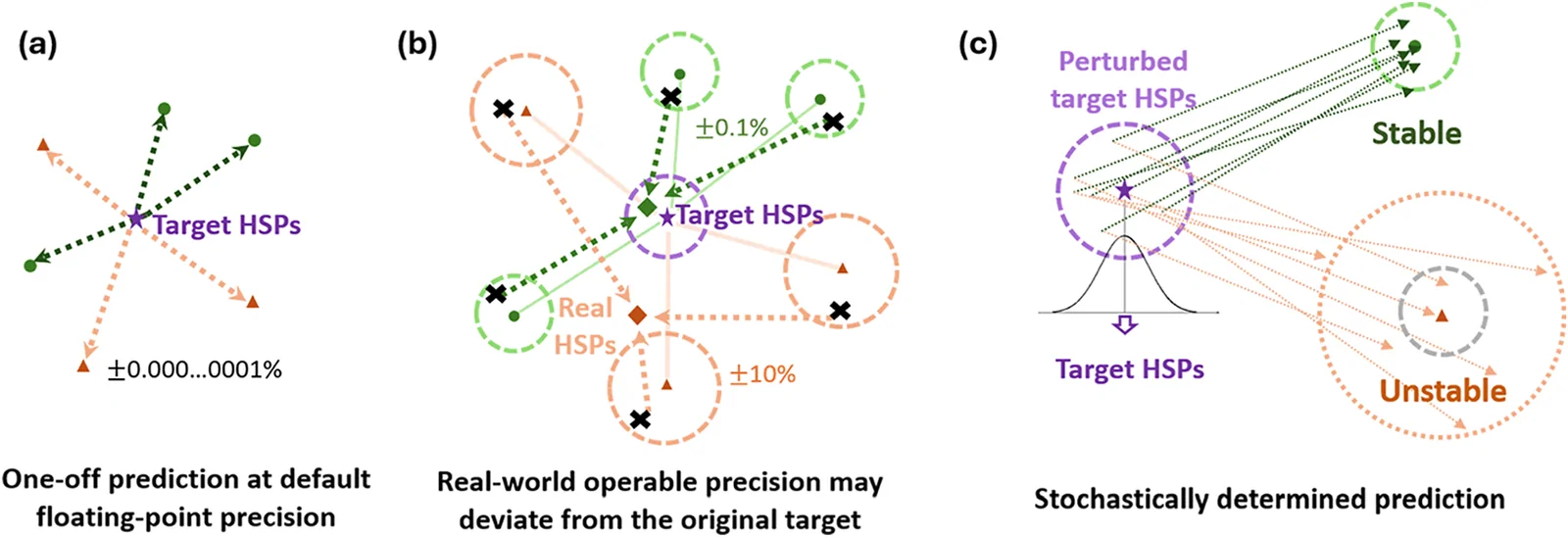
Schematic illustration of the comparison of (a) a one-off prediction at default floating-point precision *versus* (b) real-world operable precision, and (c) the perturbation strategy adopted by SolvPred. (a) A one-off prediction at default floating-point precision (10^–13^ %) yields mathematically exact but experimentally impractical compositions. (b) Experimentally sampled compositions (labelled as crosses) may deviate from the predicted values. The resulting mixture HSPs (the real HSPs, labelled as diamonds) may or may not remain acceptably close to the original target (green series–acceptable, orange series–unacceptable). A one-off prediction is unable to distinguish between these cases. (c) SolvPred expands the one-off prediction to a stochastic process: perturbations are randomly applied around the target HSPs, and stability validation is applied to distinguish stable results (the green series) from unstable ones (the orange series).

In practice, the precision of liquid compositions depends on the systematic error of the dispensing tool, the experimental scale, and the skill of operators. Selecting an appropriate precision threshold is, however, crucial, because the “real” HSPs sampled from multiple solvent concentrations that deviate from the exact prediction cannot be guaranteed to be close to the original target (Figure 4b). Since errors could either build up or cancel out by sampling multiple solvent components, a one-off prediction is insufficient to confirm the practical feasibility of a result.

To address this gap, *SolvPred* grants users the flexibility to define their own error tolerance and dynamically adapts the precision threshold to the context specified. A perturbation method has been designed to expand the one-off prediction to a stochastic process (Figure 4c). This design draws inspiration from Monte Carlo methods^73^ and perturbation theory.^74^ The key is to intentionally introduce controlled noise, using a Gaussian random number, to the target HSPs. Random sampling is then performed around the perturbed target region, such that the resulting predictions are statistically robust within the noise threshold set. A cut-off threshold based on experimentally achievable precision is defined to mark a possible sampling region around each predicted concentration. An error threshold for target HSPs is also defined, allowing to quantify the acceptable deviation between the HSPs of the actual solvent mixtures and the original targeted HSPs.

After perturbation, the actual HSPs of solvent mixtures are calculated in reverse from an ensemble of co-solvent compositions. The mean and standard deviation across all sampled predictions are compared against the user-defined error tolerance, to determine whether a prediction should be accepted or not. This allows *SolvPred* to distinguish robust (stable) solutions that consistently converge near the target from unreliable (unstable) ones that scatter widely. The deviation of stable results is expected to match the variability of induced noise level (see S1.1.4 for details).

For predictions that have passed the stability and error check, *SolvPred* then examines the redundancy of solvents. Solvents below a tolerance of ultra-low concentration are regarded as negligible for experiments. These solvents will be filtered out, with the remaining co-solvents renormalized to a total concentration of 100% and iterating through the error filtering steps.

A general practical recommendation for redundancy threshold selection is provided in S1.1.4, and the number of mixtures with respect to different levels of errors and redundancy thresholds is discussed in SI S1.4.

### Property Filtering

Once the calculation is completed, users can choose to filter the results according to the boiling point (Scheme 2a) and miscibility (Scheme 2b) of co-solvents (S1.1.5). Boling points and miscibility information have been included in the built-in database. To filter by miscibility, the database IDs (also known as the primary keys) of all immiscible solvents are compiled as an independent descriptor of each solvent. Within each predicted mixture, if the database ID of any co-solvent exists in the immiscible solvent ID, this mixture will be flagged as potentially immiscible.

**Scheme 2:**
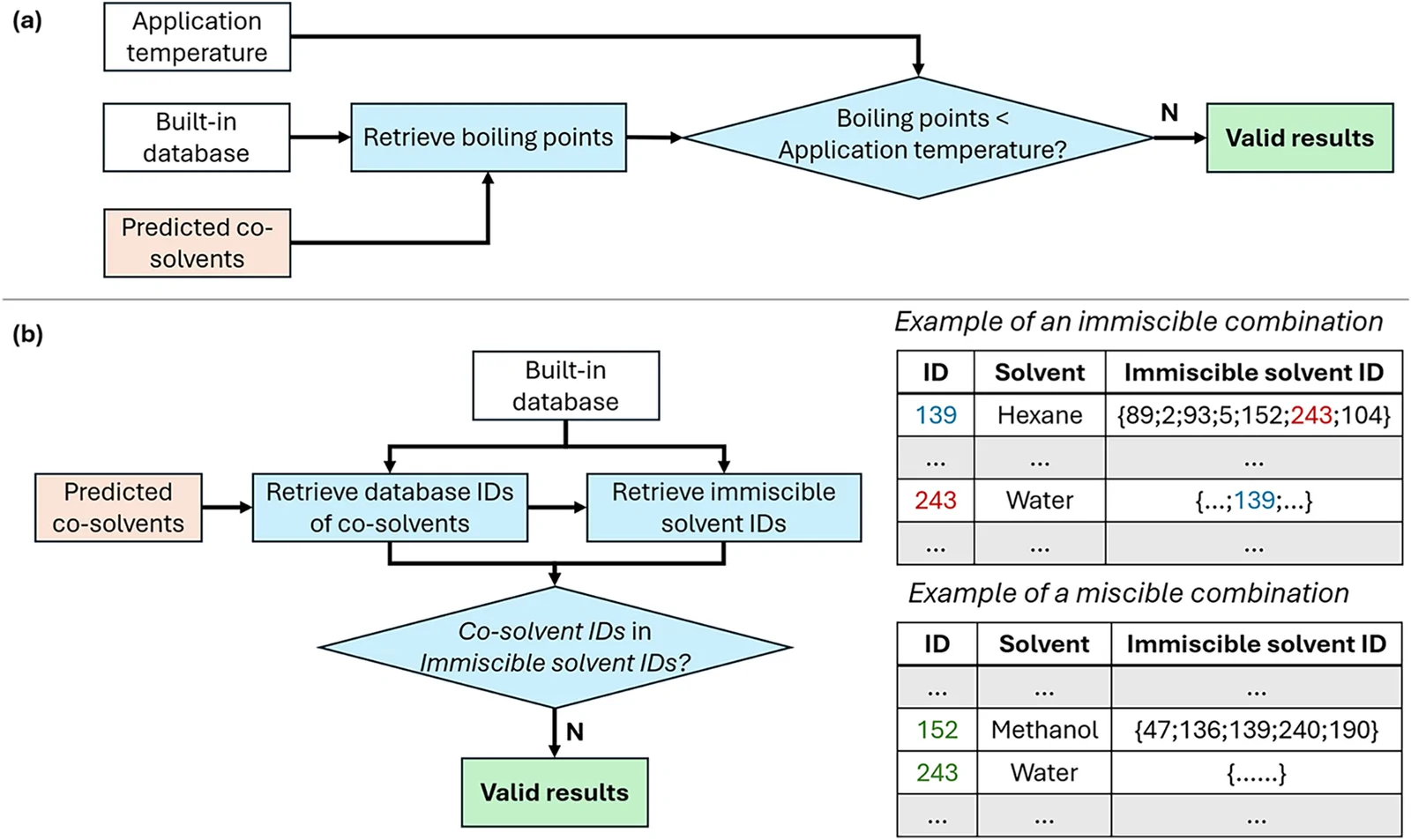
Workflows to Filter Results by Co-Solvent (a) Boiling Points and (b) Miscibility

In addition to the main workflow, a candidate filtering module was developed, which allows users to apply additional safety and physicochemical constraints. This addresses the limitations of HSPs, as these parameters are not capturing all physicochemical properties relevant to specific application scenarios.

Recognizing that such restrictions are often diverse and context-dependent, we developed a modular feature-merging workflow (Scheme 3a and S1.2). Candidates can be broadly filtered by two criteria: descriptive or physical properties (S1.2.1), and structural or chemical features (S1.2.2). The first branch retrieves safety keywords according to the GHS classifications^75^ and physical properties via the *PubChem* web API;^72^ the second branch computes chemical descriptors and subgraph patterns using *RDKit*.^70^ Users can define required properties independently and iteratively merge results to satisfy higher-level constraints (S1.2.3).

**Scheme 3:**
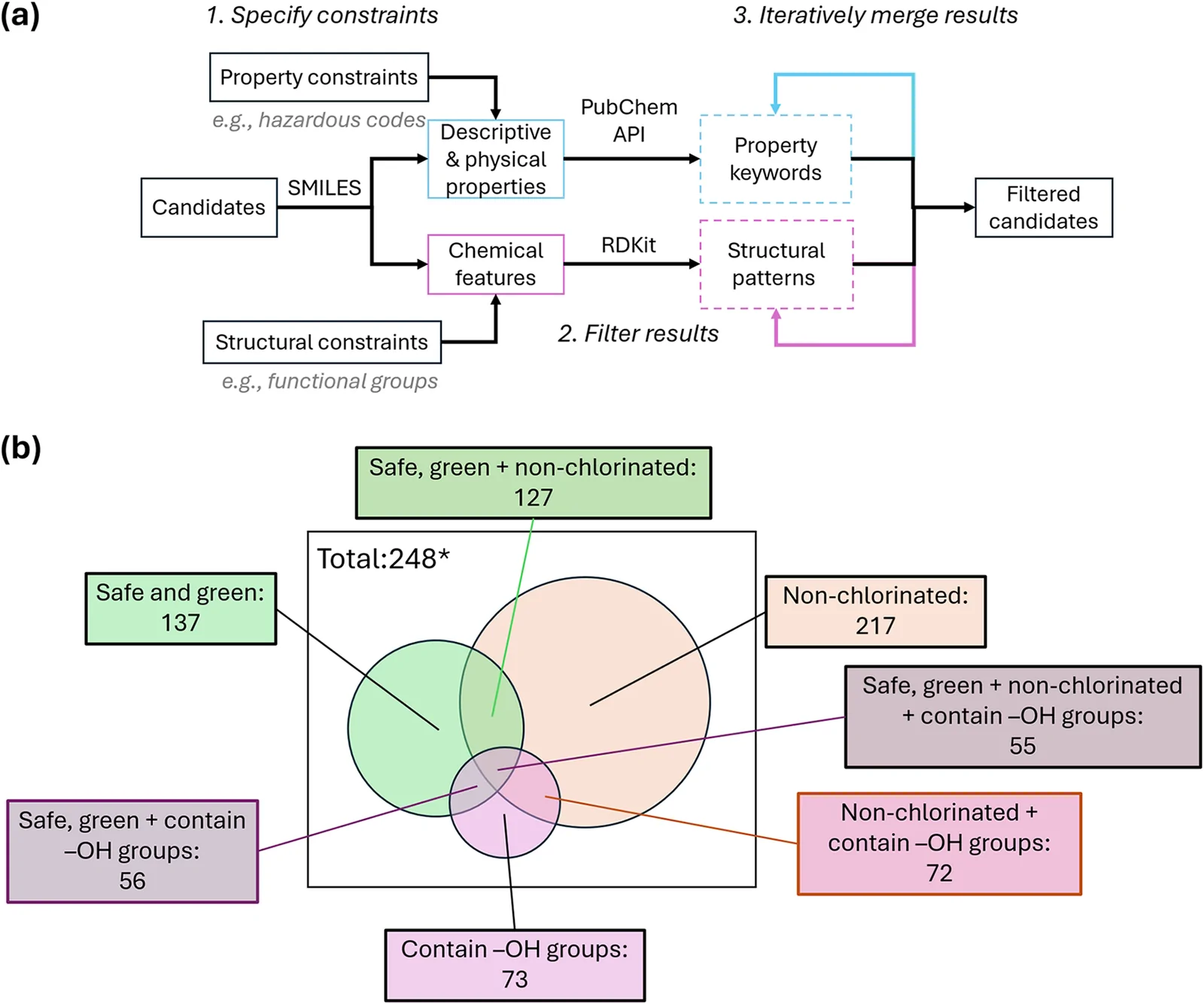
(a) An Extendible Solvent Candidate Filtering Workflow Based on Descriptive Properties and Chemical Features; (b) Example Candidate Subsets Filtered by Safety Information, Environmental Hazards and Two Representative Structural Patterns (Non-Chlorinated Species and Species Containing Hydroxyl Groups)^a^

a The number in each box is the number of filtered candidates. *One solvent (naphtha) is excluded from the filtering workflow due to unavailable SMILES information – this is because naphtha is a hydrocarbon mixture. The missing SMILES can cause failures in parsing CID or RDKit descriptors. This solvent is kept in the built-in HSP database and this would not affect the inverse calculation based solely on HSPs.

With the candidate filtering module, we provided 11 example subsets, filtering by safety keywords, environmental hazards and two representative structural patterns covering non-chlorinated species and solvents containing hydroxyl groups (Scheme 3b and Table S5). The safety keywords and environmental hazards were filtered by *PubChem* API according to the GHS classifications–solvents classified as “acute toxic (GHS06)”, “environmental hazard (GHS09)” and “health hazard (GHS08) were flagged and ruled out. These subsets are readily accessible on GitHub and can be fed into the prediction workflow as alternative candidates. Users can modify the constraints from the template code for these examples as needed. Working examples of notebooks and corresponding API queries have been provided in S1.2 and are explained in detail there.

The main *SolvPred* workflow has been integrated into a command-line-based user interface (S1.3.1) and an executable program with GUI (S1.3.2). A typical design workflow is summarized in Scheme 4. A step-by-step guide for *SolvPred* implementation and working examples are provided in S1.3 and on GitHub.

**Scheme 4:**
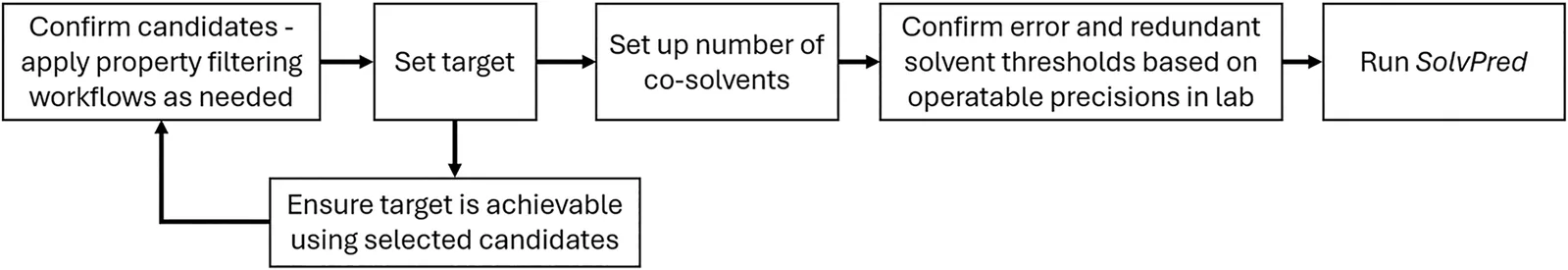
A Typical Design Workflow for SolvPred

### Case Study: Inverse Design of Solvent Mixtures for a Polyimide Condensation Reaction

To demonstrate an application of *SolvPred*, we tested its performance in a polyimide condensation reaction. The model reaction is based on a porous polyimide derivative, **pPI-1**, synthesized by coupling melamine and perylene-3,4,9,10-tetracarboxylic dianhydride (PTCDA, Scheme 5). This reaction is conventionally conducted at 180 °C in 1-methylimidazole (NMI) and the presence of zinc acetate.^71^ Using the conditions originally reported, the yield was low (ca. 33%, S2.2), potentially due to poor solubility of the PTCDA building block.

**Scheme 5:**
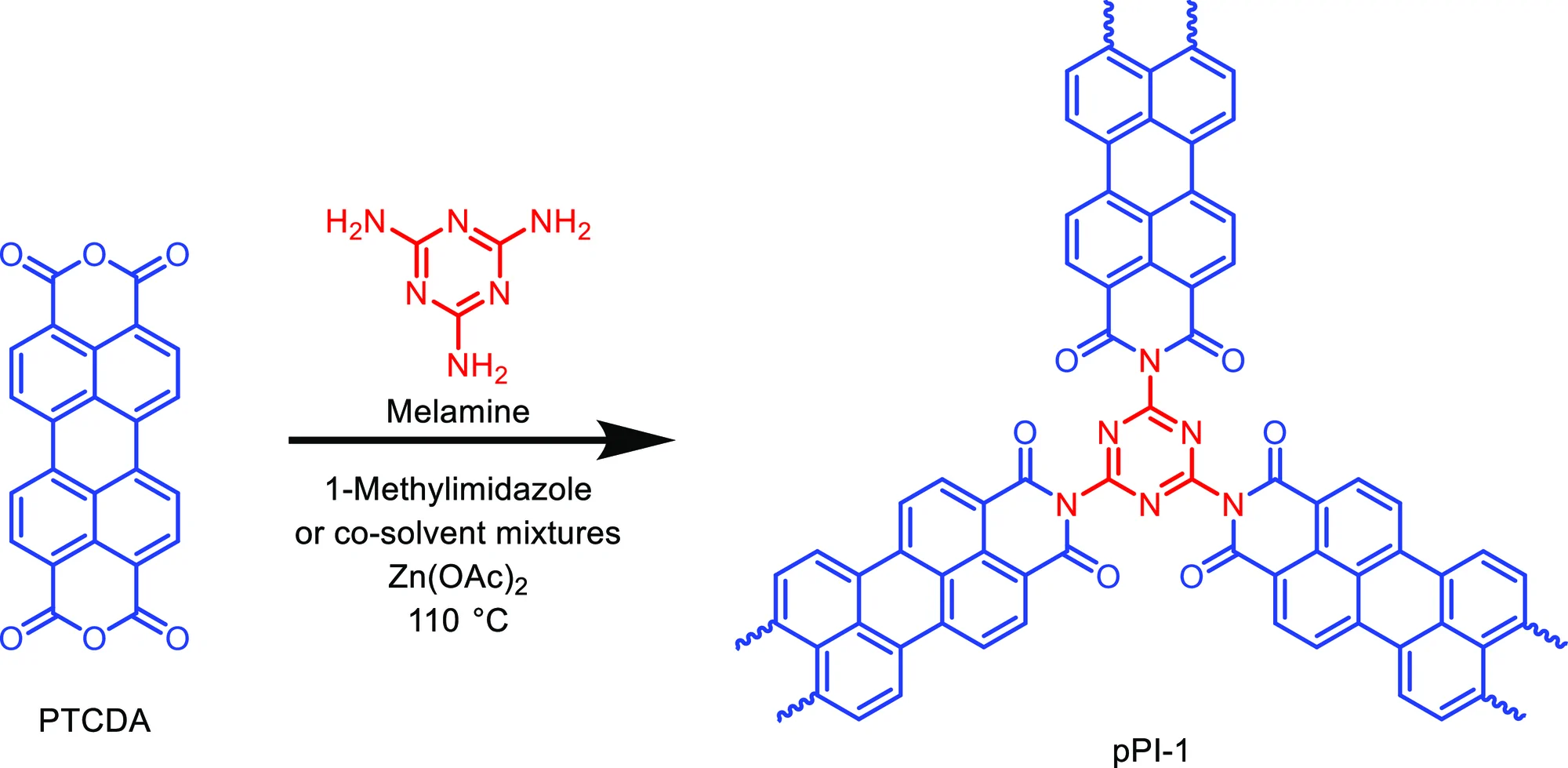
Model Reaction Scheme of the Synthesis of Porous Polyimide pPI-1 Using PTCDA and Melamine

While solvent optimization has been attempted for this reaction,^76^ we postulated that tuning HSPs can modulate the subtle solubility balance between solvents, reactants and the polymers, hence providing a route to improving the reaction yield. We specifically focused on the δ_*H*_ dimension due to its previously reported significance in analogous materials systems.^17^ Adapted from the original conditions, a series of ternary solvent mixtures were designed by *SolvPred*, systematically screening the δ_*H*_ dimension at 2.0 MPa^1/2^ intervals, while keeping the other two HSP dimensions (δ_*D*_, δ_*P*_) constant but within the user-specified errors to match those of the original single solvent (NMI).

To prepare the candidate set, we first analyzed the key requirements of this reaction. The elevated temperature necessitates the use of solvents with moderately high boiling points. The single solvent used in the original procedure, NMI, not only solubilizes PTCDA, but also functions as a base, facilitating the deprotonation of ammonium intermediates formed during this reaction, along with providing a polar environment that promotes nucleophilic attack by amines (Scheme S54). We defined the pre-filtering criteria for solvent candidates as (1) having moderately high boiling points, (2) being able to construct a polar environment and (3) containing a basic co-solvent (Scheme 6).

**Scheme 6:**
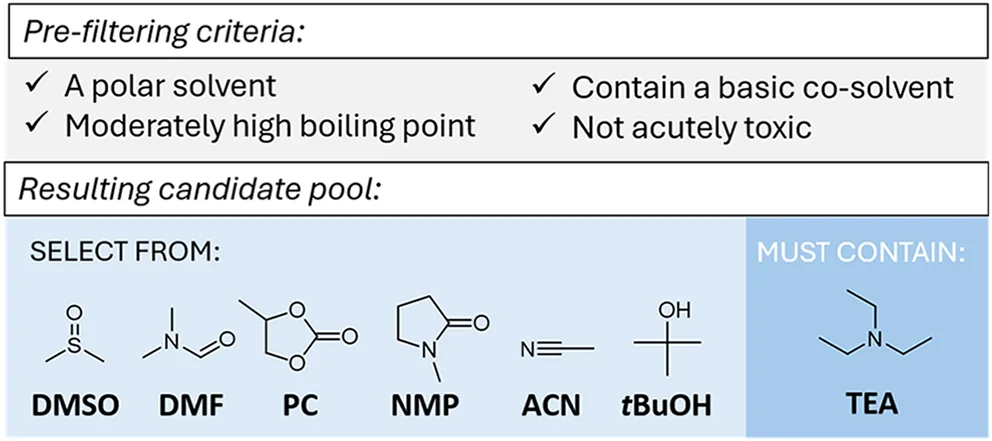
Pre-Filtering of Solvent Candidates Based on Polarity, Boiling Point, Basicity and Environmental Factors

By applying these filters, we selected a set of polar solvents with high boiling points: dimethyl sulfoxide (DMSO), dimethylformamide (DMF), propylene carbonate (PC), methyl-2-pyrroilidone (NMP), acetonitrile (ACN) and *tert*-butyl alcohol (*t*BuOH). To select a suitable basic co-solvent, we considered the three most commonly used bases in amide coupling reactions: pyridine, *N*,*N*-diisopropylethylamine (DIEA) and triethylamine (TEA).^77^ Safety constraints according to the *PubChem* GHS hazardous code were applied to exclude acutely toxic candidates (pyridine and DIEA), leaving TEA, a comparably greener reagent, as the candidate for providing basic conditions. The HSPs and physical properties of candidate solvents are provided in Table S17.

To generate solvent mixtures that selectively vary in δ_*H*_, we fixed the target δ_*D*_ and δ_*P*_ as 19.7 MPa^1/2^ and 15.6 MPa^1/2^, respectively, to be consistent with those of NMI. δ_*H*_ was modified to deviate from NMI (11.2 MPa^1/2^) in ca. 2.0 MPa^1/2^ intervals each step, yielding 9.0 MPa^1/2^, 7.0 MPa^1/2^ and 5.0 MPa^1/2^ as target δ_*H*_ using the pre-selected candidates. The error tolerance for each HSP dimension was specified as 2.0 MPa^1/2^, and the redundant solvent concentration threshold was set as 1%. This threshold was defined based on an assumed precision of measuring apparatus and the scale of the reaction, using a 40 mL-scale reaction and a measuring cylinder error of 0.5 mL in this case study. The ratio of measuring error and reaction scale (0.5 mL/40 mL) is 1%.

At this setting, even with only seven candidates, there would be 171,871 full combinatorial possibilities of ternary solvent mixtures. *SolvPred* successfully predicted seven combinations with the given target HSPs within the specified errors (2.0 MPa^1/2^). The isolated yields of pure polymeric products from reactions conducted in these mixtures are summarized in Table 1. An example snapshot of prediction outcome when target δ_*H*_ equals to 7.0 MPa^1/2^ is shown in Figure 5.

**Figure 5:**
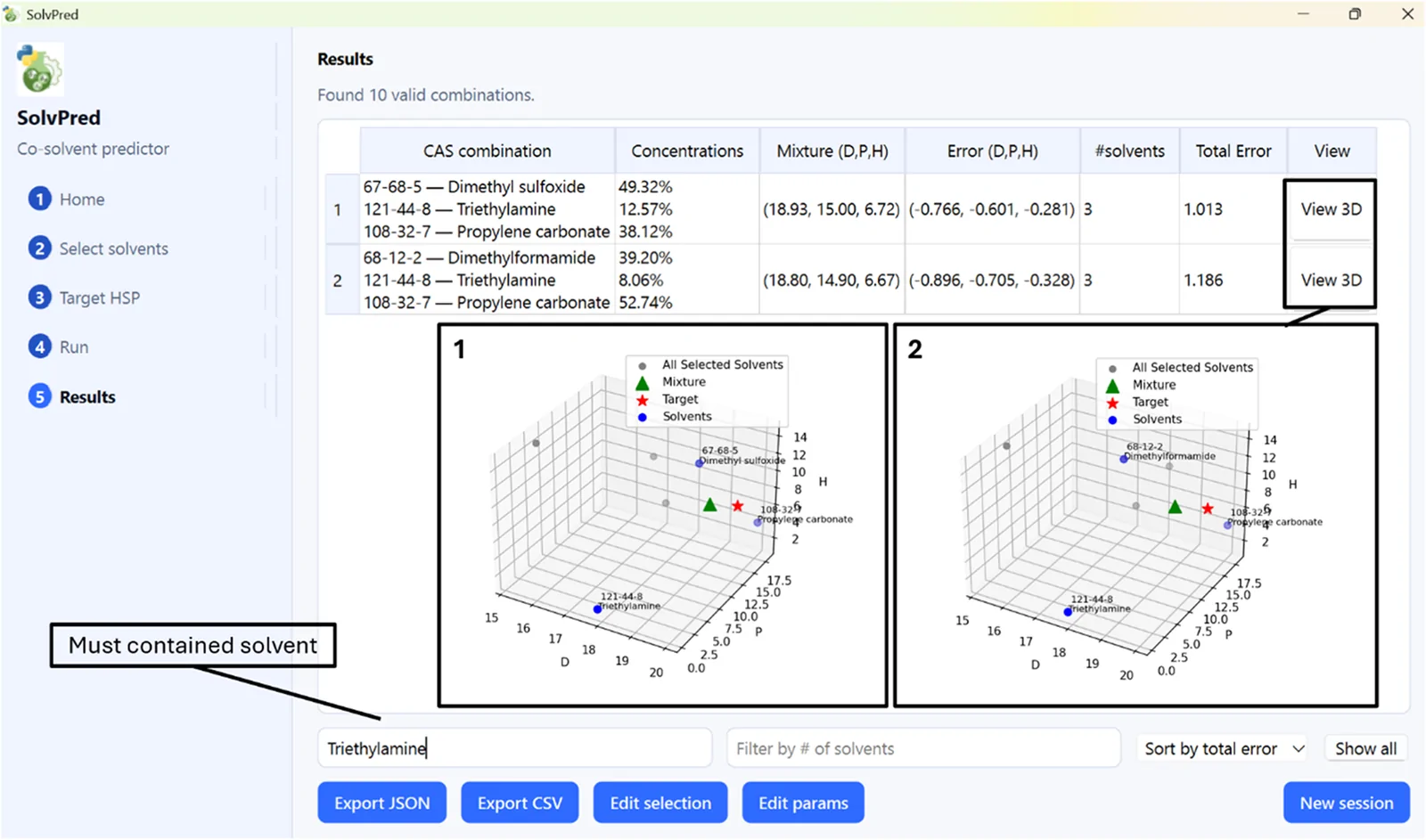
Example outcome and interface of ternary solvent prediction for target HSPs set as δ_*D*_ = 19.7 MPa^1/2^, δ_*P*_ = 15.6 MPa^1/2^, δ_*H*_ = 7.0 MPa^1/2^.

**Table 1:** Target HSPs, Predicted Mixtures, and Reaction Outcomes by Varying δ_*H*_.^c^

entry	Target δ_*H*_/MPa^1/2^	Solvent 1	Solvent 2	Solvent 3	δ_*D*_/MPa^1/2^ ^a^	δ_*P*_/MPa^1/2^ ^a^	δ_*H*_/MPa 1/2^a^	Yield/%^b^
Control (0)	-	100% NMI	-	-	19.7	15.6	11.2	16 ± 2
9A	9.0	11% PC	12% TEA	77% DMSO	18.5	14.7	8.4	NC
9B	9.0	34% PC	5% TEA	61% DMF	18.3	14.5	8.3	NC
7A	7.0	38% PC	13% TEA	49% DMSO	18.9	15.0	6.7	26 ± 5
7B	7.0	53% PC	8% TEA	39% DMF	18.8	14.9	6.7	71 ± 2
5A	5.0	67% PC	13% TEA	20% DMSO	19.4	15.4	4.9	NC
5B	5.0	73% PC	11% TEA	16% DMF	19.3	15.3	4.9	7 ± 3
5C	5.0	64% PC	5% TEA	31% NMP	19.3	15.3	4.9	NC

a The systematic variation (from 0.2 MPa^1/2^ to 0.9 MPa^1/2^) in actual δ*_D_* and δ*_P_* arises from the error specified (2.0 MPa^1/2^).The actual HSPs predicted by *SolvPred* fall within the user-specified errors.

b The yield% refers to the isolated yield of pure polymeric products – the reactions in 9A, 9B, 5A, 5C, 9N, 7N proceed to form impure mixtures of oligomeric species stacked with starting reagent PTCDA, hence denoted “NC” here for “not clean”, where no valid yield was obtained due to such impurities. Each experiment has been independently performed twice. The uncertainties represent standard deviations.

c All groups contain TEA except for the single solvent control group. All reactions were carried out at 110 °C. Yields are isolated yield of pure polymeric products.

To accommodate the use of TEA, we lowered the reaction temperature from 180 °C to a milder temperature of 110 °C and added a condenser to the reaction setup to prevent solvent evaporation. Reactions conducted in NMI at 110 °C had an average yield of 16% (entry 0), which is half of the yield obtained at 180 °C. While this result confirmed that 110 °C is less favorable than the original temperature, it also offered an opportunity to investigate whether solely tuning solvents, even under suboptimal thermal conditions, can still improve reaction outcomes.

From *SolvPred* predictions, there are two series of solvents with similar chemical compositions (PC/TEA/DMF and PC/TEA/DMSO), where only the relative concentrations of the co-solvents were varied (Figure 6a,b). We compared the isolated yield in both series. A promising solvent composition was identified when δ_*H*_ approaches 7.0 MPa^1/2^ (Figure 7), yielding an average of 26% product in the PC/TEA/DMSO group (entry 7A in Table 1) and 71% in the PC/TEA/DMF group (entry 7B in Table 1).

**Figure 6:**
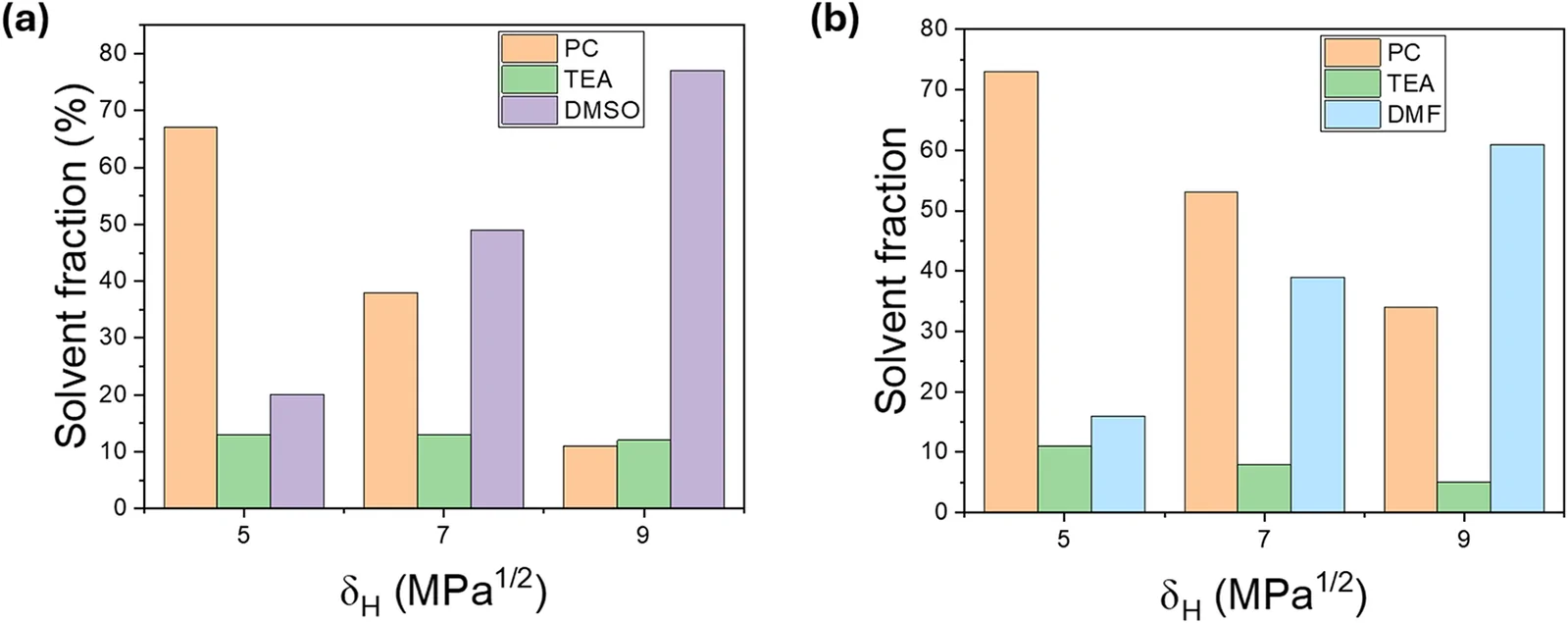
Case study: optimizing the synthesis of a polyimide condensation reaction by specifically varying δ_*H*_ using SolvPred. (a) Predicted solvent mixtures of PC/TEA/DMSO at three target δ_*H*_ values. (b) Predicted solvent mixtures of PC/TEA/DMF at three target δ_*H*_ values.

**Figure 7:**
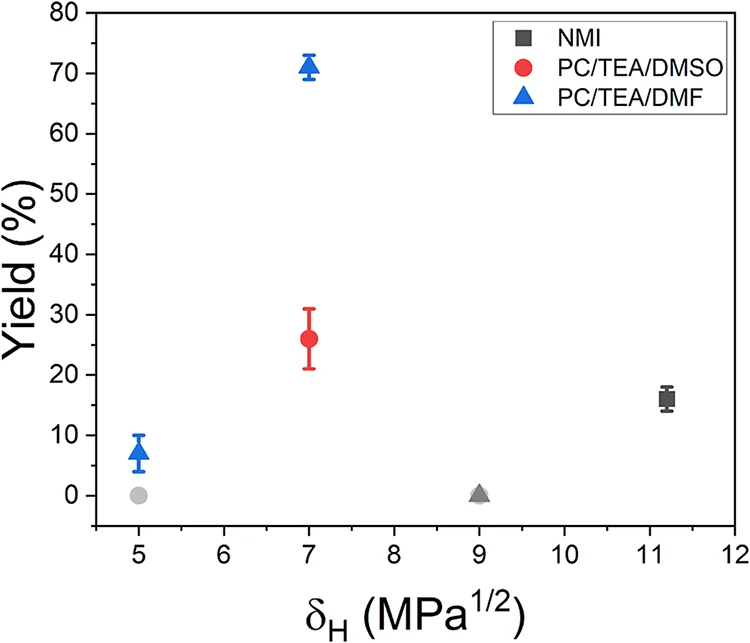
Isolated yield of the model reactions synthesized using solvent combinations of different target δ_*H*_. Gray scale points at 5.0 MPa^1/2^ and 9.0 MPa^1/2^ denote no valid yields obtained due to impurities.

Compared with the case when the target δ_*H*_ is 7.0 MPa^1/2^, no polymer product was obtained in either group when the target δ_*H*_ is set to 9.0 MPa^1/2^ (Entries 9A, 9B in Table 1). When the target δ_*H*_ is 5.0 MPa^1/2^, the PC/TEA/DMF group yielded an average of 7% product (entry 5B in Table 1), while the PC/TEA/DMSO group again yielded no product (entry 5A in Table 1). In addition to DMF and DMSO, NMP was predicted as a viable co-solvent with TEA at δ_*H*_ = 5.0 MPa^1/2^ (entry 5C in Table 1), but this combination also yielded no product.

The existence of a promising solvent environment in this work aligns well with our previously reported “over-tuning” effect of HSPs in six other POPs,^17^ where an optimal solvent–polymer distance in the Hansen space was also identified as single solvents were varied. Solvents which deviate from the promising regime could negatively affect the delicate interactions between solvent, reactants and polymers.

To further emphasize the importance of chemical constraints, we attempted the reaction at δ_*H*_ = 9.0 MPa^1/2^ and 7.0 MPa^1/2^ using solvent mixtures that excluded TEA (Table S18). A ternary mixture of 48/14/38 PC/*t*BuOH/DMF was predicted to give a target δ_*H*_ of 9.0 MPa^1/2^, and a binary mixture of 76/24 PC/tBuOH was predicted to give a target δ_*H*_ equal to 7.0 MPa^1/2^. As expected, no reaction occurred in the absence of a basic co-solvent. This result reaffirms the necessity of incorporating relevant chemical knowledge during solvent candidate selection alongside HSP theory, as considered in the *SolvPred* filtering module.

It is noteworthy that the best-performing group identified in this study, 53/8/39 PC/TEA/DMF (Group 7B), not only enhanced the yield of **pPI-1** approximately fivefold compared to the control reaction in NMI at 110 °C (71% versus 16%), but also more than doubled the yield obtained at the elevated temperature of 180 °C in NMI (71% versus 33%). Additionally, the cost of this optimal solvent mixture for the same reaction volume was only £2.04, representing a ca. 97% reduction compared to £71.20 for NMI (see S2.4 in SI for a price breakdown).

The findings in this case study highlight that the economic and green chemistry potential of *SolvPred* goes beyond the ability to efficiently optimize and interpret reaction performance, along with its capability of decoupling a specific dimension of solvent–solute interaction.

## Discussion

The impact of solvents on reactions is complex and not all aspects of this are captured by HSPs. In Table 1, clean, isolable product was obtained essentially at a single δ_*H*_ value, therefore the observed trend rests on one point rather than on a monotonic yield−δ_*H*_ relationship. In addition, whilst the target HSPs were nearly identical from each prediction, substantially different yields were obtained for solvent mixtures containing DMF and DMSO (Entries 5A, 5B and 7A, 7B), despite their similarity and comparability in all three HSPs dimensions.

We propose that this discrepancy originates from structural differences between solvent molecules, along with broader complexity arising from different macroscopic properties, such as viscosity (0.8 cP for DMF vs 2.0 cP for DMSO). Similar outcomes were noticed when removing TEA from the model reaction, where it had acted as the base, not just as solvent, as discussed in Table S18. Solvent-specific chemistry, *e.g.*, coordination of Zn catalysts, that HSPs theory does not fully describe, may also contribute to the problem.

In addition, systematic variation may occur around the target values depending on the user-defined error thresholds. This aspect needs to be considered as these thresholds can affect the independent contribution of a specific HSP dimension.

Therefore, we emphasize that HSPs can be used to define a promising search region within which solvent-specific chemistry determines the outcome. The *SolvPred* algorithm will not replace chemical knowledge about the target application scenarios, but instead can help to avoid exhaustive calculations and experiments on unsuitable candidates, as well as supporting the interpretation of results.

We also note that the workflow assumes linear mixing of HSPs, which can limit its applicability. Although the linear mixing approximation is widely accepted in the HSP literature, it may not be universally valid and may become inaccurate for mixtures exhibiting strong specific interactions (including hydrogen bonding), or significant non-ideal behavior, as observed for DMSO, DMF and PC in the case study reported here.

For properties that are not captured by HSPs, such as basicity, their scaling is also not guaranteed by the linear mixing rule. In the case study presented, the p*K*_a_ of the conjugated acid of the original single solvent (NMI) is 7.4, lower than the intentionally introduced base TEA (10.7). However, while the fraction of TEA varied from 5% to 13%, the corresponding yield did not scale proportionally. A catalytic amount of base may have been sufficient to initiate the reaction, with the actual reaction outcome dominated by other solvent–solute/catalyst interactions.

Since HSP theory originates from studies of large organic molecules and polymers, we expect *SolvPred* to be primarily suitable for applications in polymer science. Application to small or charged molecules (*e.g.*, inorganic salts or zwitterions) and organometallic chemistry have not been tested in this study, but such scenarios may not be adequately described by HSP theory and may require extra solvent descriptors.

Additionally, the physical meaning of the dipolar and hydrogen-bonding interactions needs to be interpreted with care for non-polar molecules with multiple functional groups, which, for the dipolar term, is because the local polar groups cannot be explicitly expressed by the overall δ_*P*_. For the hydrogen-bonding term, it is a residue term, generated by subtracting the induced-dipole and dipole fractions out of the total cohesive energy. This means that δ_*H*_ theoretically contains any additional non-covalent molecular interactions and cannot represent explicit donor or acceptor effects, hence should not be over-interpreted solely in terms of its values.

The potential uncertainties in the underlying HSP database are also worth considering when selecting an appropriate resolution for target HSPs and error filtering thresholds for *SolvPred*. Given that HSP data are reported to one decimal place, we recommend the same or lower resolution for target HSPs and error thresholds (*i.e.*, > 0.1 MPa^1/2^), to keep the calculations meaningful. A detailed sensitivity test based on the assumption of linear HSP mixing using different resolutions of errors (2.0 MPa^1/2^, 1.0 MPa^1/2^ and 0.1 MPa^1/2^) and redundant solvent thresholds (1.0%, 0.1%, 0.01%) has been carried out and is included in Section S1.4.

Due to the one-to-many mapping nature of inverse design, there will be more than one prediction for a set of target HSPs. The number of results depends on the number of candidates and the relative location of the target with respect to these candidates (explained in SI S1.4). Solutions obtained in *SolvPred* are ranked by their deviation from the target HSPs, as well as a filtering option based on a “must contain” co-solvent (for example triethylamine in the case study) and the number of co-solvents to narrow down the results. An example is given in the SI, Scheme S51. Deviations within each HSP dimension, along with the total errors, are systematically recorded in the calculation log (Scheme S33) and presented to users for references.

## Conclusions and Future Work

In this work, we present *SolvPred*, an open-source co-solvent prediction workflow for fine-tuning HSPs. This framework enables a proof-of-concept inverse design of solvent components within a quasi-continuous solubility space, while incorporating practical and extensible filtering criteria. With *SolvPred*, we systematically optimized solvent conditions for a porous polyimide derivative for yield improvement. We also demonstrated that rational co-solvent design can outperform traditional reaction variables such as temperatures, and offers a potential reduction in both cost and energy consumption during chemical process optimization. It is also essential to acknowledge that HSP theory has some limitations, in that explicit solvent behavior cannot be fully captured and because it relies on a linear mixing assumption. Therefore, we would encourage users of our algorithm to keep solvent-specific chemistry within the promising search region that HSPs have defined in mind. We envision *SolvPred* as an efficient property-guided solvent design strategy in data-driven chemistry. Future studies, using this tool in different and more diverse solvent design scenarios, are encouraged to be considered with this in mind.

## Supplementary Material



## Data Availability

The data underlying this study are available in the electronic supporting information documents. The supporting information for this manuscript contains additional information on the underlying theory, further details of *SolvPred* and the experimental work reported, as well as relevant references. All code and databases underlying this study are openly available on GitHub and via Zenodo at the following URLs: The production-ready command-line version of *SolvPred*: https://github.com/xueannafang/hsp_toolkit_solv_pred_v_2.0; 10.5281/zenodo.22081059; The production-ready graphical user-interface version of *SolvPred*: https://github.com/xueannafang/SolvPred_App; 10.5281/zenodo.20785883.

## Notes

Additional computational details, algorithm design principles, implementation workflows, working examples and experimental details, materials, and methods, including characterization and analysis (PDF)
