# Supplementary material for: An Inverse Design Workflow for Solvent Mixtures Based on Hansen Solubility Parameters
Source: J Chem Inf Model. 2026 Oct 2;66(20):13370–82. doi: 10.1021/acs.jcim.6c02092 (PMC13715477; doi:10.1021/acs.jcim.6c02092)
Supplement: Supplementary file 1 [file ci6c02092_si_001.pdf]

An inverse design workflow for solvent mixtures based on Hansen solubility parameters

Supplementary information

Xue Fang,[a,b] * Shaohua Li,[a,c] Bo Gao,[d] Ella M. Gale,[a] Charl F. J. Faul, [a]* Natalie Fey [a]*

[a]School of Chemistry, University of Bristol, Cantock's Close, Bristol, BS8 1TS, UK

[b] Molecular Sciences Research Hub, Imperial College London, White city campus, 82 Wood Lane, London, W12 0BZ, UK

[c] Department of Chemical Engineering, Imperial College London, South Kensington campus, London, SW7 2AZ, UK

[d] School of Physics, HH Wills Physics Laboratory, University of Bristol, Tyndall Avenue, Bristol, BS8 1TL, UK

*Corresponding authors

Keywords: Hansen solubility parameters, solvent mixtures, inverse design of solvents

Table of contents

S1 Supporting information for *SolvPred* S3

S1.1 Details of the *SolvPred* co-solvent prediction algorithm S3

S1.1.1 *SolvPred* database S3

S1.1.2 Temperature correction S6

S1.1.3 Mathematical details of the *SolvPred* algorithm S7

S1.1.4 Details of the perturbation strategy S8

S1.1.5 Details of properties filtering S12

S1.2 Extendible solvent candidate filtering module S13

S1.2.1 The descriptive and physical information filtering branch S15

S1.2.2 The chemical information filtering branch S19

S1.2.3 Merging multiple features S24

S1.3 User interfaces and working examples S26

S1.3.1 Command line-based user interface S26

S1.3.2 Graphical user interface of *SolvPred* S39

S1.4 Computing performance and sensitivity tests S50

S2 Supporting information for experimental case study S60
S2.1 Chemicals S60
S2.2 Synthesis of **pPI-1** in solvents with varied δH S60
S2.3 Characterization S66
S2.4 Economic impact evaluation of predicted solvent mixtures S71

S1 Supporting information for *SolvPred*

SolvPred is developed using Python 3.9.12. Key dependencies of the *SolvPred* Python API include numpy (version 1.21.5) and scipy.linalg (version 1.7.3). The executable program, *SolvPred_App*, is developed under the PyQt6 framework.

The *SolvPred* workflow has been tested on Microsoft Windows 10 as the operating system, a 3.20 GHz CPU (Intel Core i7-8700 processor), and 16 GB random-access memory (RAM), as well as Windows 11, 2.10 GHz CPU (Intel Core i7-1260P), with 16 GB RAM. The calculation requires negligible computing resources and commonly completes within seconds.

The *SolvPred* API can be installed by cloning the repository from GitHub using the following command at the user-specified working directory:

```
git clone https://github.com/xueannafang/hsp_toolkit_solv_pred_v_2.0
```

To install the environment for the API, run the following commands in Anaconda prompt:

```
conda create -n solvpred python=3.9.12
```

```
conda activate solvpred
```

```
pip install numpy==1.21.5 scipy==1.7.3
```

Go to the working directory where the SolvPred API Github repository has been cloned using the following command:

```
cd “C:\...\hsp_toolkit_solv_pred_v_2.0”
```

and run the solv_pred_main.py script as detailed in S1.3.1

The *SolvPred* APP can be installed by cloning the repository from GitHub using the following command:

```
git clone https://github.com/xueannafang/SolvPred_App.git
```

The step-by-step guide is provided in S1.3.2.

S1.1 Details of the *SolvPred* co-solvent prediction algorithm

S1.1.1 *SolvPred* database

For the SolvPred database (v2.0), we have compiled HSPs of 249 solvents from the HSP user’s guidebook.[1] Additional solvent features, including physicochemical properties,

miscibility information, SMILES and synonyms parsed from *PubChem*,[2] as detailed in Table S 1, have also been included to support property filtering.

Table S 1 Solvent properties included in the database of SolvPred v2.0

Column name	Explanation
No.	Index of this solvent in this database, also known as the database ID or a primary key
CAS	CAS No. of corresponding solvent
Name	Solvent name
D	Dispersion HSP, unit: $MPa^{1/2}$
P	Dipolar HSP, unit: $MPa^{1/2}$
H	Hydrogen bond HSP, unit: $MPa^{1/2}$
Mole_vol	Molar volume, unit: cm^3mol^{-1}
ims_idx	Index of immiscible entries in this database
Bp	Boiling point, unit: °C
mw	Molecular weight, unit: $gmol^{-1}$
viscosity	Viscosity of corresponding solvent, unit: Pa·s
vis_temp	Temperature at which the viscosity is measured, unit: °C
heat_of_vap	Molar heat of evaporation, unit: $kJmol^{-1}$
hov_temp	Temperature at which the heat_of_vap is measured, unit: °C
SMILES	Simplified molecular-input line-entry system of the corresponding solvent
alias	Common abbreviations of corresponding solvent
synonyms	Common synonyms of corresponding solvent

A snapshot of the *SolvPred* database is provided in Scheme S 1.

No.	CAS	Name	D	P	H	Mole_vol	ims_idx
1	75-07-0	Acetaldehyde	14.7	12.5	7.9	56.6	
2	64-19-7	Acetic acid	14.5	8	13.5	57.1	139;190;39
3	108-24-7	Acetic anhydride	16	11.7	10.2	94.5	40
4	67-64-1	Acetone	15.5	10.4	7	74	
5	75-05-8	Acetonitrile	15.3	18	6.1	52.6	139;47;136;240;190
6	98-86-2	Acetophenone	19.6	8.6	3.7	117.4	243
7	107-13-1	Acrylonitrile	16	12.8	6.8	67.1	243
8	107-18-6	Allyl alcohol	16.2	10.8	16.8	68.4	
9	628-63-7	Amyl acetate	15.8	3.3	6.1	148	243
10	62-53-3	Aniline	19.4	5.1	10.2	91.5	
11	100-66-3	Anisole	17.8	4.1	6.7	119.1	243
12	100-52-7	Benzaldehyde	19.4	7.4	5.3	101.5	
13	71-43-2	Benzene	18.4	0	2	89.4	243;14;27;69;74;104;116;120;135;214;220;233
14	108-46-3	1,3-Benzenediol	18	8.4	21	87.5	13;44;120
15	65-85-0	Benzoic acid	18.2	6.9	9.8	113.1	243

bp	mw	viscosity	vis_temp	heat_of_vap	hov_temp	SMILES	alias	synonyms
20.8	44.05	0.21	20	25.73	20.2	O=CC		acetic aldehyde;ethyl aldehyde
117	60.05	1.06	25	23.7	117.9	CC(=O)O		ethanoic acid;Ethylic acid;Vinegar acid
139	102.09	0.84	25	38.2	139.5	CC(=O)OC(=O)C		Acetyl acetate;Acetanhydride;Ethanoic anhydride
56.2	58.08	0.32	20	29.1	56.05	CC(=O)C		2-propanone
81.6	41.05	0.35	20	29.81	80	CC#N	ACN	Methyl cyanide;Cyanomethane
202	120.15	1.68	-1	49	-1	CC(=O)C1=CC=CC=C1		1-Phenylethanone;Methyl phenyl ketone;Acetylbenzene
77.2	53.06	0.34	25	32.6	25	C=CC#N		2-Propenenitrile;Polyacrylonitrile;Prop-2-enenitrile
96.9	58.08	1.22	25	40	97.4	C=CCO		2-Propen-1-ol;Vinylcarbinol
149.2	130.18	1.58	11	38.42	149.2	CCCCCOC(=O)C		Pentyl acetate;n-Amyl acetate
184	93.13	4.35	20	55.83	-1	C1=CC=C(C=C1)N		Benzenamine;Phenylamine;Aminobenzene
155.5	108.14	0.78	30	46.84	25	COC1=CC=CC=C1		Methoxybenzene;Methyl phenyl ether
178.7	106.12	1.32	25	42.5	179	C1=CC=C(C=C1)C=O		Benzoic aldehyde;Phenylmethanal
80.08	78.11	0.6	25	33.83	25	C1=CC=CC=C1		benzol

Scheme S 1 A snapshot of the SolvPred database. Any entry with "-1" means data missing.

The SolvPred database can be customized by users as needed. To do so, users can copy and edit the "`db_solv_pred_v2.json`" file, and add a new entry in the example format shown in Figure S 1. The built-in database for the command-line version can be found in the GitHub repository, within the main working directory at the same level with the "`solv_pred_main.py`" script; for the GUI version, the built-in databases and candidate lists are available in the "app > data" folder.

The "No." should continue from the final entry in the database – for example, if extending from the 249-solvent database, the No. of the next new solvent should start from "250". It is essential to upload the correct "CAS", "Name", "D", "P", "H" in the new solvent entry, as these are key properties to ensure a correct calculation. Depending on data availability, a solvent may lack reported data of other properties listed in the database, which can be specified as "-1". "ims_idx" entry needs a special formatting: if users know immiscible solvents of the new entry, users need to refer to the indices of the corresponding solvents in the built-in database and combine those as a string separated by ";", without spaces in between, as their "ims_idx". If immiscible information is unknown, the "ims_idx" can be either left as empty (None) or removed from the list of keys for this specific solvent.

```
{
  "No.": 248,
  "CAS": "108-20-3",
  "Name": "Isopropyl Ether",
  "D": 15.1,
  "P": 3.2,
  "H": 3.2,
  "Mole_vol": 140.9,
  "ims_idx": "89;243",
  "bp": 68.5,
  "mw": 102.17,
  "viscosity": 0.273,
  "vis_temp": -6,
  "heat_of_vap": 32.51,
  "hov_temp": -1,
  "SMILES": "CC(C)OC(C)C",
  "synonyms": "2-Isopropoxypropane"
},
```

Figure S 1 Example format of an individual solvent in the built-in database.

For users preferring a graphical interface, users can also consider editing the .csv or .xlsx versions of this database, exemplified on GitHub as "db_mis.xlsx" or "db_filtered.csv" for those with property filtering scores, followed by converting the csv to json format using `pandas`.

S1.1.2 Temperature correction

The thermal expansion correction is carried out using reference equations provided by HSPs user's guide book[2]:

$$\delta_D(T) = \delta_D(298)(1 - 1.25\alpha\Delta T), \qquad \textit{Eq. S 1}$$

$$\delta_P(T) = \delta_P(298)(1 - 0.5\alpha\Delta T), \qquad \textit{Eq. S 2}$$

$$\delta_H(T) = \delta_H(298)[1 - (0.00122 + 0.5\alpha)\Delta T], \qquad \textit{Eq. S 3}$$

where α is the thermal expansion coefficient (default: 0.0007/K) and ΔT is the temperature difference between working temperature and room temperature (298 K); all scalers are provided by the original HSPs user guidebook. The unit of temperature is Kelvin (K).

The HSP thermal expansion theory was initially determined by Hansen and Beerbower in 1971, by fitting semi-empirical temperature-dependent measurements of cohesive energies.[3–5] The equations here have been then developed and implemented in a commercial HSP software *HSPiP*.[5]

S1.1.3 Mathematical details of the *SolvPred* algorithm

The *SolvPred* algorithm is detailed in Scheme S 2.

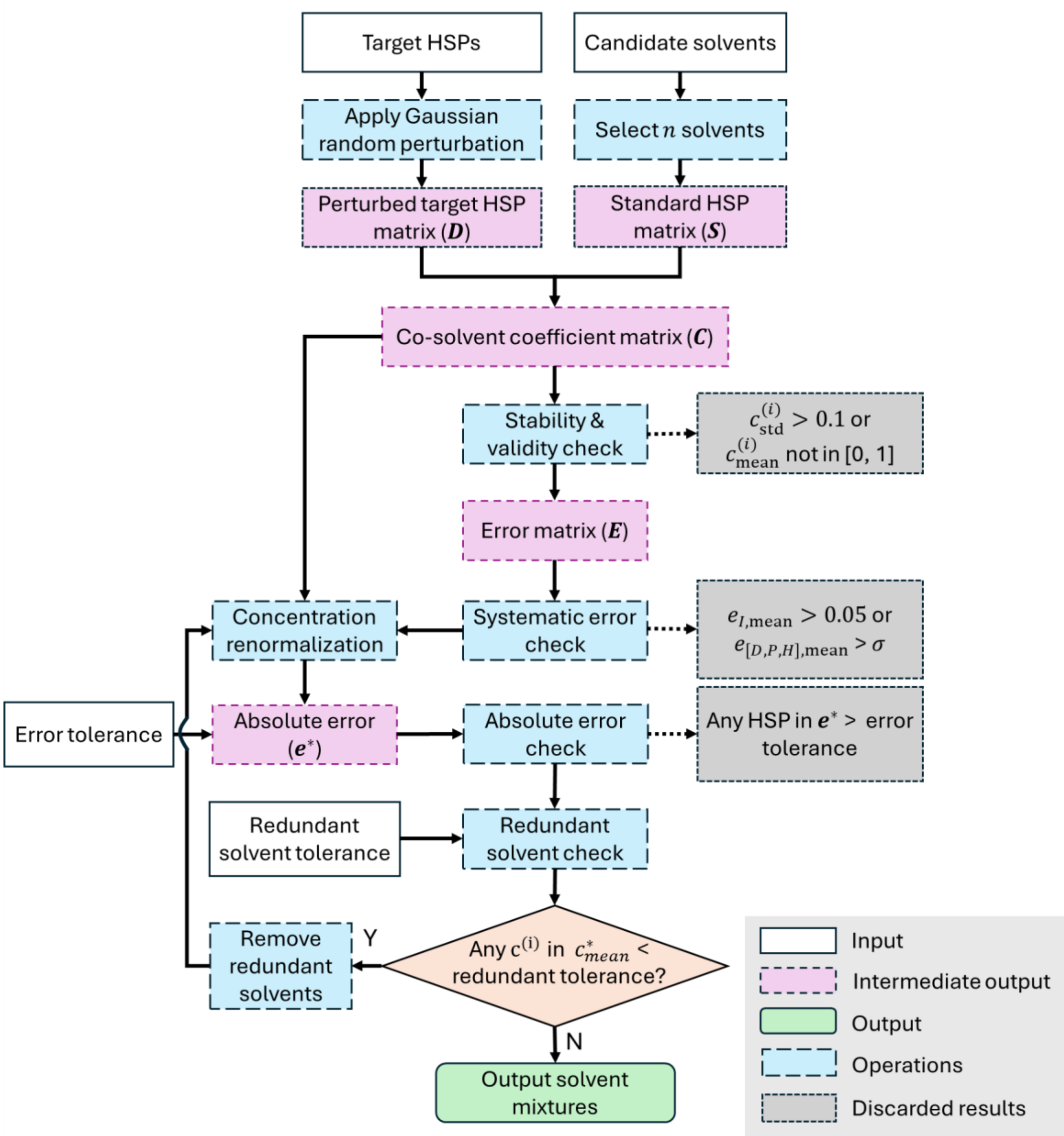

Scheme S 2 An overview of the core algorithm of SolvPred.

The target HSP vector is:

$$\boldsymbol{\delta}_{target} = [\delta_D \quad \delta_P \quad \delta_H]^T \; . \qquad \textit{Eq. S 4}$$

The solvent candidate HSPs matrix is:

$$\boldsymbol{\delta}_{cand} = [\boldsymbol{\delta}_{cand}^{(1)} \quad \boldsymbol{\delta}_{cand}^{(2)} \quad \dots \quad \boldsymbol{\delta}_{cand}^{(i)} \quad \dots \quad \boldsymbol{\delta}_{cand}^{(n)}]^T \; , \qquad \textit{Eq. S 5}$$

with $\boldsymbol{\delta}_{cand}^{(i)}$ being the HSPs of the ith solvent,

$$\boldsymbol{\delta}_{cand}^{(i)} = \left[\delta_D^{(i)} \quad \delta_P^{(i)} \quad \delta_H^{(i)}\right]^T. \qquad \textit{Eq. S 6}$$

Vector $\boldsymbol{c}$ is the coefficient vector:

$$\boldsymbol{c} = [c_1 \quad c_2 \quad \cdots \quad c_i \quad \cdots \quad c_n]^T. \qquad \textit{Eq. S 7}$$

A constraint is applied to vector $\boldsymbol{c}$ to ensure that the sum of co-solvent concentrations equals 1:

$$\sum_{i=1}^{n} c_i = 1. \qquad \textit{Eq. S 8}$$

To apply this constraint, a normalization factor, 1, is added as the final row in $\boldsymbol{\delta}_{\mathbf{target}}$ and in each of the $\boldsymbol{\delta}_{cand}^{(i)}$ vectors, yielding the updated target HSP vector,

$$\boldsymbol{d} = [\delta_D \quad \delta_P \quad \delta_H \quad 1]^T, \qquad \textit{Eq. S 9}$$

and the standard HSPs matrix,

$$\boldsymbol{S} = \begin{bmatrix} \delta_D^{(1)} & \delta_D^{(2)} & \cdots & \delta_D^{(i)} & \cdots & \delta_D^{(n)} \\ \delta_P^{(1)} & \delta_P^{(2)} & \cdots & \delta_P^{(i)} & \cdots & \delta_P^{(n)} \\ \delta_H^{(1)} & \delta_H^{(2)} & \cdots & \delta_H^{(i)} & \cdots & \delta_H^{(n)} \\ 1 & 1 & \cdots & 1 & \cdots & 1 \end{bmatrix}. \qquad \textit{Eq. S 10}$$

$\boldsymbol{c}$ can be solved by moving $\boldsymbol{S}$ to the right-hand side, followed by solving the left pseudoinverse of $\boldsymbol{S}$ *via* singular value decomposition (SVD) implemented in Python *scipy* package.[6]

$$\boldsymbol{Sc} = \boldsymbol{d}. \qquad \textit{Eq. S 11}$$

$$\boldsymbol{c} = \boldsymbol{S}^{-1}\boldsymbol{d}. \qquad \textit{Eq. S 12}$$

S1.1.4 Details of the perturbation strategy

Dynamic precision control is necessary because there are no universal thresholds of experimentally-operable dispensing precision: a Gilson pipette can typically achieve higher precision than a Pasteur pipette; larger-scale experiments (*e.g.*, over 50 ml) may tolerate less precise measurements whereas smaller-scale screening (*e.g.*, below 2 ml) is likely to be more sensitive to subtle volume differences and demands tighter control; even for the same measuring apparatus, the actual precision can vary between an experienced researcher and a beginner.

Taking a P1000 Gilson pipette as an example, the systematic error is typically between 3 to 8 µL. If we use this pipette to prepare 10 mL of a 10/90 (v/v) mixture, the dispensing error, for the 10% component, would be $\pm$ 0.003 to 0.008 mL. Approximating this as $\pm$ 0.01 mL, the corresponding precision threshold is 0.01/10*100% = 0.1%. For the same dispensing task performed with a Pasteur pipette, assuming *ca.* 20 drops per mL, the resolution will change to 50 µL by drop and yield 0.5% as the precision threshold instead.

As a general rule-of-thumb, we recommend using the ratio between the error of measuring apparatus and the scale of experiment to estimate the redundancy threshold.

Introducing a stochastic element has been previously proven effective in other inverse design problems. For example, in the evolution of autoencoder to variational autoencoder, the noise introduced intentionally to the input would allow the model to identify more robust latent representations and hyperparameters.[7] This idea has been adapted in *SolvPred,* repeating one-off predictions many times while adding statistical perturbation.

Random, repeated sampling around the target HSPs simulates the actual HSPs that are achievable by experiments. Results with a comparable level of standard deviation can be regarded as stable (c_a series in Scheme S 3); otherwise, the results are unstable (c_b series in Scheme S 3). This resembles the philosophy of perturbation theory, where describing a real state *via* finding the exact solution of the Schrödinger equation is challenging. A perturbing operator that is scaled by a factor between 0 and 1 is applied, allowing the algorithm to map a solvable state to the state of interest.

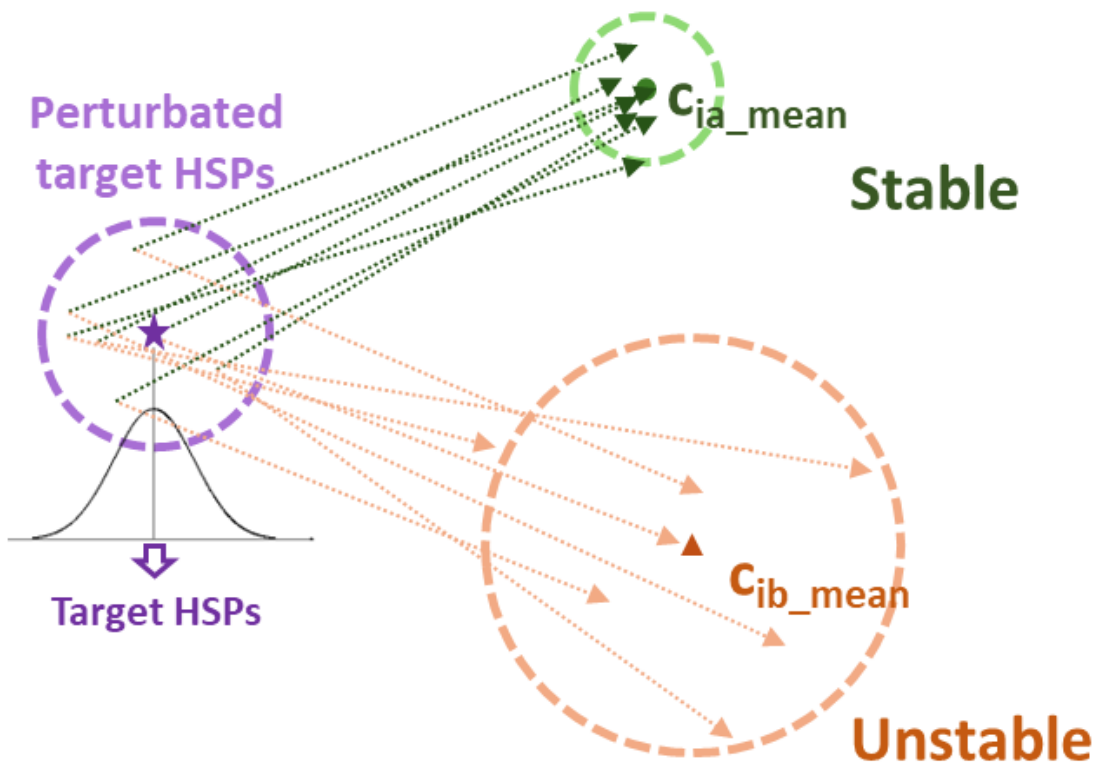

Scheme S 3 Illustration of using Gaussian-random perturbation to distinguish between stable (c_a series) and unstable results (c_b series).

To add controlled noise, each target HSP vector is perturbed using a series of Gaussian random numbers (GRNs, Eq. S 13. Eq. S 14). This constructs a batch of randomly sampled target HSPs, expanding $\boldsymbol{d}$ (Eq. S 9) into a perturbed target HSPs matrix, $\boldsymbol{D}$ (Eq. S 15), with each element centered on the original target HSPs but varied within a user-defined standard deviation.

Here, the Gaussian random number (GRN) follows the following form:

$$GRN = A\frac{1}{\sigma\sqrt{2\pi}}exp\left\{-\frac{(x-\mu)^2}{2\sigma^2}\right\}, \qquad Eq.\ S\ 13$$

where A is the scale factor of the Gaussian perturbation, representing the maximum deviation from the target HSPs (default 0.1); x is the target HSP; μ and σ correspond to mean and standard deviation of the Gaussian distribution, respectively. By default, the

values of μ and σ are 0 $MPa^{1/2}$ and 0.1 $MPa^{1/2}$, respectively. The GRN hyperparameters are adjustable in the *SolvPred* source code where needed.

Applying the default GRN allows a ± 0.1 fluctuation centred on the exact target, which updates all elements in the target HSPs vector into:

$$\delta^{*}_{D,P,H} = \delta_{D,P,H} + GRN. \qquad \textit{Eq. S 14}$$

This yields the target HSP matrix, $\boldsymbol{D}$:

$$\boldsymbol{D} = \begin{bmatrix} \delta_D^{*(1)} & \delta_D^{*(2)} & \dots & \delta_D^{*(j)} & \dots & \delta_D^{*(t)} \\ \delta_P^{*(1)} & \delta_P^{*(2)} & \dots & \delta_P^{*(j)} & \dots & \delta_P^{*(t)} \\ \delta_H^{*(1)} & \delta_H^{*(2)} & \dots & \delta_H^{*(j)} & \dots & \delta_H^{*(t)} \\ 1 & 1 & \dots & 1 & \dots & 1 \end{bmatrix}, \qquad \textit{Eq. S 15}$$

where $\delta^{*(j)}_{D,P,H}$ are the jth perturbed target HSPs (Eq. S 14); t denotes the total times of applied perturbation.

Coefficients, or concentrations, of solvent candidates, are now expanded from vector $\boldsymbol{c}$ (Eq. S 7) to a perturbed form, $\boldsymbol{C}$ (Eq. S 16), that calculates coefficients for each perturbed target HSP:

$$\boldsymbol{C} = \boldsymbol{S}^{-1}\boldsymbol{D} = \begin{bmatrix} c_{11} & c_{12} & \dots & c_{1j} & \dots & c_{1t} \\ c_{21} & c_{22} & \dots & c_{2j} & \dots & c_{2t} \\ \dots & \dots & \dots & \dots & \dots & \dots \\ c_{i1} & c_{i2} & \dots & c_{ij} & \dots & c_{it} \\ \dots & \dots & \dots & \dots & \dots & \dots \\ c_{n1} & c_{n2} & \dots & c_{nj} & \dots & c_{nt} \end{bmatrix}. \qquad \textit{Eq. S 16}$$

SolvPred then carries out a stability assessment by evaluating the average ($c^{(i)}_{\mathrm{mean}}$) and standard deviation ($c^{(i)}_{\mathrm{std}}$) of the resulting coefficient sets over all attempts of perturbation (Eq. S 17, Eq. S 18). For each solvent i, the standard deviation and mean of its concentration over all perturbations are:

$$c^{(i)}_{mean} = \frac{\sum_{j=1}^{t} c_{ij}}{t}. \qquad \textit{Eq. S 17}$$

$$c^{(i)}_{std} = \sqrt{\frac{\sum_{j=1}^{t} (c_{ij} - c^{(i)}_{mean})^2}{t}}, \qquad \textit{Eq. S 18}$$

The assumption is that stable predictions cluster within a well-defined region around a central point, whereas unstable predictions are scattered more broadly. A result is considered "stable" if its output variability matches the input perturbation level and its standard deviation is less than 10%; otherwise, it is filtered out. As such, any results with $c^{(i)}_{\mathrm{std}}$ larger than 0.1, equivalent to an over 10% deviation over repeated perturbations, are filtered out. These results are unlikely to yield stable predictions over repeated perturbations.

To maintain physical meaning, the average coefficient over all perturbation is also required to be between 0 and 1. Results beyond this region are physically invalid because solutions with co-solvents of less than 0% or over 100% exist in the mixture. These results either do not hold valid physical meaning or breach the 100% total concentration constraint. Therefore, any results involving $\mathrm{c}_{\mathrm{mean}}^{(\mathrm{i})}$ above 1 or below 0 are filtered out.

Next, the predicted coefficient matrix ($\boldsymbol{C}$) is multiplied back into the standard HSPs matrix ($\boldsymbol{S}$) to calculate the real HSPs for the predicted mixture. The real HSP matrix ($\boldsymbol{SC}$) is compared with the perturbed target HSP matrix ($\boldsymbol{D}$) to yield the error matrix ($\boldsymbol{E}$, Eq. S 19).

$$\boldsymbol{E} = \boldsymbol{SC} - \boldsymbol{D} = \begin{bmatrix} e_D^{(1)} & e_D^{(2)} & \cdots & e_D^{(j)} & \cdots & e_D^{(t)} \\ e_P^{(1)} & e_P^{(2)} & \cdots & e_P^{(j)} & \cdots & e_P^{(t)} \\ e_H^{(1)} & e_H^{(2)} & \cdots & e_H^{(j)} & \cdots & e_H^{(t)} \\ e_I^{(1)} & e_I^{(2)} & \cdots & e_I^{(j)} & \cdots & e_I^{(t)} \end{bmatrix}. \qquad Eq.\ S\ 19$$

The average ($\mathrm{e}_{[\mathrm{D,P,H,I}],mean}$) and standard deviation ($\mathrm{e}_{[\mathrm{D,P,H,I}],\mathrm{std}}$) of the error over all perturbations (Eq. S 20, Eq. S 21), are calculated to evaluate the invalid results. Both systematic and absolute errors are considered.

$$e_{[D,P,H,I],mean} = \frac{\sum_{j=1}^{t} e_{D,P,H,I}^{(j)}}{t}, \qquad Eq.\ S\ 20$$

$$e_{[D,P,H,I],std} = \sqrt{\frac{\sum_{j=1}^{t}\left(e_{D,P,H,I}^{(j)} - e_{[D,P,H,I],mean}\right)^2}{t}}. \qquad Eq.\ S\ 21$$

Systematic errors are evaluated based on the deviation from the normalization factor, reflected by the deviation of $e_{I,\mathrm{mean}}$ in Eq. S 20 from 1. Results with $e_{\mathrm{I,mean}}$ larger than 0.05, corresponding to a total average concentration of over 105% or below 95%, are removed. Results with $e_{[\mathrm{D,P,H}],\mathrm{mean}}$ over the standard deviation of the applied GRNs in Eq. S 13 are also filtered out, due to the mismatching of the output variability and the input perturbation level.

To get absolute errors, $c_{\mathrm{mean}}^{(i)}$ of results that have passed the previous validation are first renormalized, to ensure the average total concentration is 100% (Eq. S 22 to Eq. S 25). This is necessary because while the total coefficients within each perturbation are constrained to 100%, the perturbation may cause the total average coefficients to deviate from 100%, *i.e.*, while for the jth perturbation,

$$\sum_{i=1}^{n} c_j^{(i)} = 1, \qquad Eq.\ S\ 22$$

taking the average over all perturbations, there exists a deviation of the total average concentration from 1,

$$\sum_{i=1}^{n} c_{mean}^{(i)} \neq 1, \qquad Eq.\ S\ 23$$

The results that have passed the previous stability validation therefore need to be renormalized first for each solvent according to,

$$c_{mean}^{*(i)} = \frac{c_{mean}^{(i)}}{\sum_{i=1}^{n} c_{mean}^{(i)}}, \qquad Eq.\ S\ 24$$

constructing the renormalized coefficient vector

$$\boldsymbol{c}_{\boldsymbol{mean}}^{*} = [c_{mean}^{*(1)} \quad c_{mean}^{*(2)} \quad \cdots \quad c_{mean}^{*(i)} \quad \cdots \quad c_{mean}^{*(n)}]^{T}, \qquad Eq.\ S\ 25$$

for absolute error calculation and filtering.

The renormalized coefficient vector, $\boldsymbol{c}_{\boldsymbol{mean}}^{*}$ (Eq. S 25) is replaced back in Eq. S 11 to calculate the absolute error ($\boldsymbol{e}^{*}$) with respect to the target HSPs:

$$\boldsymbol{e}^{*} = |\boldsymbol{S}\boldsymbol{c}_{\boldsymbol{mean}}^{*} - \boldsymbol{d}| = [e_D^{*} \quad e_P^{*} \quad e_H^{*} \quad e_I^{*}]. \qquad Eq.\ S\ 26$$

Users can specify the error tolerance for each dimension of HSP. Results with errors exceeding the corresponding tolerance are removed.

Results after removing redundant solvents will repeat the renormalization–error filtering steps, to ensure that the final results have a total concentration of 100%, with errors remaining close enough to the user-defined error threshold.

S1.1.5 Details of properties filtering

In addition to the main calculation steps, *SolvPred* applies an advanced filtering option, allowing users to consider additional properties. In the current version, these properties include boiling points and miscibility, which have been registered in the *SolvPred* database.

In the current workflow, the boiling point of each co-solvent is fetched from the *SolvPred* database, followed by comparing with the working temperature specified at the beginning of this module. Any solvent with a boiling point below working temperature can be flagged, as it may cause evaporation during the application, thus introducing either inaccuracy or safety concerns. Users may keep those results and apply other experimental strategies, such as a condenser, to proceed with the original predictions.

The miscibility is evaluated by comparing immiscible solvent pairs in the built-in database logged in the “ims_idx” column shown in Scheme S 1. We compiled immiscible solvent information from *PubChem* and added those to the *SolvPred* database as an independent feature. By checking whether the database ID of any co-solvent exists in the immiscible solvent indices list, immiscible solvent pairs can be identified and flagged. For example, Entry 13 (benzene) is immiscible with Entry 243 (water), Entry 14 (1,3-benzenediol), Entry 27 (1,3-butanediol), Entry 69 (*o*-dichlorobenzene), Entry 74

(diethylene glycol), Entry 104 (ethanolamine) and Entry 11 (anisole). If any solvents are predicted in the same mixture group with benzene, the results will be flagged in the output log file.

However, we need to note that miscibility data are assumed to be measured at room temperature. Miscibility at elevated temperatures is subject to change, whereas reported data are limited. Therefore, the results only provide a guideline for practical applications. For the boiling point filtering, we also recognize that certain experimental setups, such as reactions done in a sealed pressure reactor, may not always require low boiling point solvents to be filtered. To address such variability, the indeterminate property filtering step is set to be optional. Results provided in this step have all been saved in a full log document, both before and after the filtering is carried out.

S1.2 Extendible solvent candidate filtering module

The workflow of the extendible solvent candidate filtering module is illustrated in Scheme S 4.

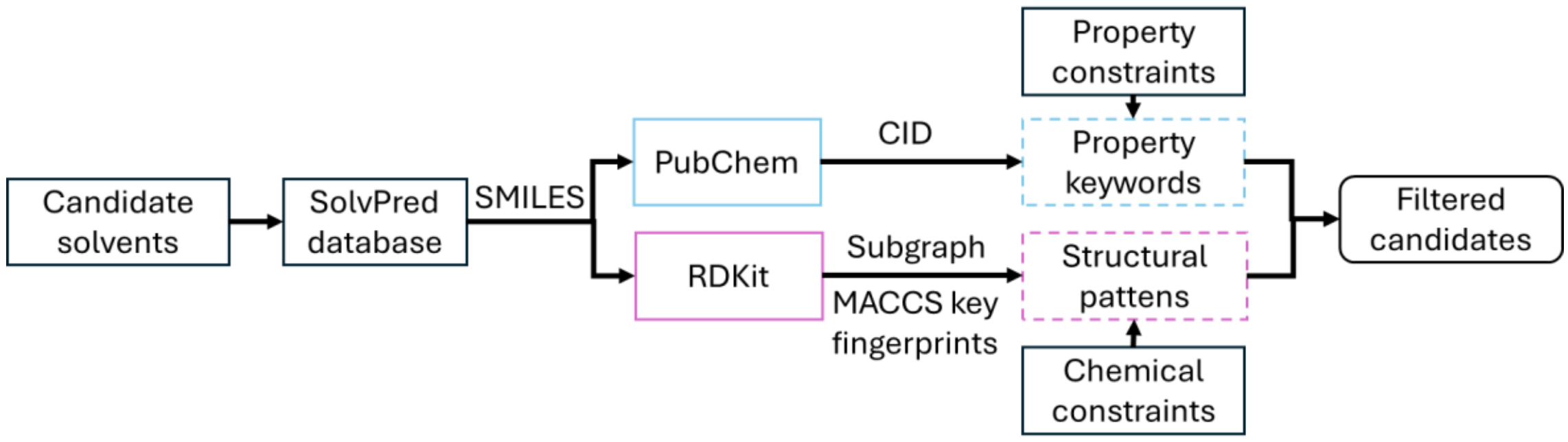

Scheme S 4 The workflow of the extendible solvent candidate filtering module.

Users begin by supplying an initial candidate set with solvent CAS No. The workflow then retrieves SMILES strings from the built-in *SolvPred* database for downstream processing. Two filtering branches, focusing on 1) the descriptive and physical information and 2) the chemical information, are available.

In the first branch, a compound ID (CID) is generated from each SMILES string via the *PubChem* web API. The CID is used to query metadata from *PubChem*.[2] This enables retrieval of safety keywords, physical property data (*e.g.*, viscosity, boiling point), along with other information relevant to solvent selection. Users can specify the feature of interest to extract target information, followed by specifying the keywords of concern as the "property constraints". Any candidates matching these constraints will be flagged and filtered. An example of filtering safety keywords is demonstrated in S1.2.1.

The second branch, detailed in S1.2.2, focuses on filtering by chemical knowledge. This branch employs structure-based filtering *via* fragment recognition and MACCS key fingerprints analysis to identify solvents with or without chemical patterns of interest.

Simple patterns, such as elemental composition, functional groups, aromaticity or proticity, can be assessed by checking the relevant MACCS key bits. *SolvPred* summarizes MACCS key positions of ten chemistry-sensitive solvent patterns, including the presence of halogens (fluorine, chlorine, bromine), phosphorus atoms, primary amines, secondary amines, hydroxyl groups, carbonyl groups, sulfinyl groups, and aromatic rings (Table S 3). Larger functionalities and connection motifs, such as broad classes of esters, ethers, nitriles, aliphatic and aromatic alcohols, linear alkanes, as well as specific moieties such as imidazole rings, nitro benzene rings, and pyridyl rings, have been evaluated using the *Fragments* method in *RDKit*.[8]

We included forty-four representative substructures in the template notebook detailed in Table S 4. These patterns are readily customizable by users, allowing them to apply chemical constraints to target problems.

In either branch, users can define filtering criteria independently. When multiple features are needed, the results filtered through independent parent features can be iteratively merged to form an intersection candidate set that satisfies higher-level constraints (S1.2.3). This design ensures flexibility and extendibility without overly complicating the candidate selection.

Filtering of candidates can be conducted by running "*solv_pred_cand_prep_by_safety.py*" and "*solv_pred_pre_filt_FG.py*", respectively, using auxiliary template notebooks "*candidate_prepare_by_safety_info.ipynb*" and "*candidate_prepare_by_FG.ipynb*". The former retrieves CID and associated compound data from *PubChem*. The latter extracts structural patterns, including molecular subgraphs and a molecular access system fingerprint vector (MACCS key fingerprints), using *RDKit*. Filtered candidates are saved in both *.csv* and *.json* formats, to feed into the main *SolvPred* algorithm.

To filter multiple features, individual features can be filtered separately using the relevant Jupyter notebook, followed by feeding the output files to "*Merge_candidate_set.ipynb*" to extract solvents at the intersection of such criteria. This step can be iteratively conducted as required by users.

Three example template notebooks: "*candidate_prepare_by_safety_info.ipynb*", "*candidate_prepare_by_FG.ipynb*", and "*Merge_candidate_set.ipynb*", along with example input/output documents, can be found in the "*ext_cand_filt*" folder in the *SolvPred* Github repository. These resources are summarized in Table S 2. A demonstration is given in the following sections.

Table S 2 Auxiliary scripts, template notebooks and example resources to support candidate filtering. All scripts are in the "ext_cand_filt" folder in SolvPred GitHub repository if not specified otherwise.

Filename	Explanation
solv_pred_cand_prep_by_safety.py	A template script for filtering solvent candidates by safety information

candidate_prepare_by_safety_info.ipynb	A template notebook for filtering solvent candidates by safety information
db_with_safe_info_reform.csv	An extended solvent database with pre-extracted safety keywords of each solvent
db_with_safe_info_classified (csv/json)	A solvent database including safety and environmental hazard filter columns
safe_green_candidates (csv/json)	An example filtered candidate list of solvents that have passed the safe and green solvent check
get_hazard_info.ipynb	A template notebook for parsing hazardous information or equivalent accessible properties from *PubChem* to query solvent safety information based on the GHS hazard classifications *via PubChem* API.
solv_pred_pre_filt_FG.py	A template script for filtering functional groups and substructures of solvent candidates
candidate_prepare_by_FG.ipynb	A template notebook for filtering functional groups and substructures of solvent candidates
Merge_candidate_set.ipynb	A template notebook for merging subsets selected by different parent features

S1.2.1 The descriptive and physical information filtering branch

Requirements and resources:

Packages required in this branch include *pandas*, *json*, *numpy* and *requests*.

A template Python script and an auxiliary notebook are provided in "*solv_pred_cand_prep_by_safety.py*" and "*candidate_prepare_by_safety_info.ipynb*". An extended solvent database with all pre-extracted safety keywords from *PubChem* is available in "*db_with_safe_info_reform.csv*" and this is given as an example to run the filtering functions in the template scripts.

In this branch, a CID is generated for each entry from its SMILES. Metadata of full information recorded for the corresponding compound is retrieved from *PubChem via PUG REST* and *PUG VIEW* APIs. Users can specify the feature of interest, for example, as "*Chemical Safety*", in the "*TOCHeading*" tag to extract target information. This retrieves all relevant safety keywords, covering "Flammable", "Irritant", "Health Hazard", "Corrosive", "Acute Toxic", "Environmental Hazard" and "Compressed Gas". Users then specify the keywords of concern as the "safety constraints", and any candidates

matching these constraints will be automatically flagged and filtered. Depending on the specific requirements, the "*TOCHeading*" tag can be adjusted to other PubChem features or extended to metadata linking to external databases (*e.g.*, "*ChEMBL ID*", "*DrugBank ID*", "*CCDC Number*", *etc*.)

To filter solvents by safety keywords, users can specify target keywords in an "*undesired feature list*". In the example shown in Scheme S 5, undesired features are listed in the "*concern_list*" variable, covering "*AcuteToxic (GHS06)*", "*EnvironmentalHazard (GHS09)*" and "*HealthHazard (GHS08)*". Solvents with any of these features are undesired candidates. To filter solvents without relevant safety concerns, run the "*get_cand_without_safety_concern*" function, where the "*full_data_dict*" argument is the input dataset, and the "concern" argument is the undesired feature list (*i.e.*, the "*concern_list*").

```
#Please modify your safety concern list, example:
concern_list = ["AcuteToxic", "EnvironmentalHazard", "HealthHazard"]
```

```
#Load the database with safety keywords
file_name = "db_with_safe_info_reform.csv"
```

```
db_safe_df = pd.read_csv(file_name)
db_safe_dict = db_safe_df.to_dict("records")
```

```
def get_cand_without_safety_concern(full_data_dict = db_safe_dict, concern = ["AcuteToxic", "EnvironmentalHazard"]):
    safe_subset = []
    for entry in full_data_dict:
        safe_concern_found = 0
        entry["concern_type"] = []
        this_safety_kw = entry["safety_kw"]
#          print(this_safety_kw)
        if this_safety_kw is not np.NAN:
            for kw in this_safety_kw[1:-1].replace("'", "").split(","):
                if kw in concern:
                    safe_concern_found = 1
                    entry["concern_type"].append(kw)

            if safe_concern_found == 0:
                entry["is_concern"] = "False"
                safe_subset.append(entry)
                entry["concern_type"] = -1
            else:
                entry["is_concern"] = "True"
        else:
            entry["is_concern"] = -1
            entry["concern_type"] = -1
            safe_subset.append(entry)

    return safe_subset, full_data_dict
```

```
safe_subset, db_filt = get_cand_without_safety_concern(full_data_dict = db_safe_dict, concern = concern_list)
```

Scheme S 5 Implementation of safety keywords filtering.

After the filtering is completed, users can selectively export results that classify each candidate based on whether it contains any concern types, or directly export the filtered candidate list (Scheme S 6).

```
#save subset and full data with safety classifided information

#specify your file reference, e.g., non_health_hazard
file_ref = "non_toxic_non_health_hazard_green"

#save full db with classified information
db_with_safe_info_classified = pd.DataFrame(db_filt)
db_with_safe_info_classified.to_csv(f"db_with_safe_info_classified_{file_ref}.csv", index = None)
db_with_safe_info_classified.to_json(f"db_with_safe_info_classified_{file_ref}.json", orient = "records")

#save db with candidate subset only
safe_subset_df = pd.DataFrame(safe_subset)
safe_and_green_candidates = pd.DataFrame()
safe_and_green_candidates["CAS"] = safe_subset_df["CAS"]
safe_and_green_candidates["Solvent"] = safe_subset_df["Name"]
# safe_and_green_candidates.head()
safe_and_green_candidates.to_csv(f"{file_ref}_candidates.csv", index = None)
safe_and_green_candidates.to_json(f"{file_ref}_candidates.json", orient ="records")
```

Scheme S 6 Exporting results of filtered candidates.

The classified results, serving as user references, extend the input candidate list by flagging any existing target safety keywords in the "*concern_type*" column, and scoring each entry with a binary tag in the "*is_concern*" column (Scheme S 7). If no hazard is found, the "*concern_type*" is denoted "-1", with "*is_concern*" labelled "*False*". Results fitting into this category pass the safety check and are saved as the filtered results for the next stage calculation.

safety_kw	is_concern	concern_type
['Flammable', 'Irritant', 'HealthHazard']	True	['HealthHazard']
['Flammable', 'Corrosive']	False	-1
['Flammable', 'Corrosive', 'Irritant']	False	-1
['Flammable', 'Irritant']	False	-1
['Flammable', 'Irritant']	False	-1
['Irritant']	False	-1
['Flammable', 'Corrosive', 'AcuteToxic', 'HealthHazard', 'EnvironmentalHazard']	True	['AcuteToxic', 'HealthHazard', 'EnvironmentalHazard']
['Flammable', 'AcuteToxic', 'EnvironmentalHazard']	True	['AcuteToxic', 'EnvironmentalHazard']

Scheme S 7 Classified candidates based on the safety keyword check. Results passing the safety keyword check are kept for the downstream applications.

Examples of output documents are given in "*db_with_safe_info_classified (csv/json)*" and "*safe_green_candidates (csv/json)*". The first is the database extended with safety information; the latter is the filtered candidates that have passed the safe and green solvent checks.

Users can follow the structure of the template notebook "*get_hazard_info.ipynb*" to extend filtering keywords to other accessible features on *PubChem*. The workflow is to get CID from SMILES, followed by pulling the full data record from *PubChem*. Users can specify the name of target sections to include, and iterate through the content to extract relevant information (Scheme S 8).

General workflow

```
sm = "CC1=CC=CC=C1" #toluene
cid = get_cid_by_smiles(sm)
print(f"cid:{cid}")
full_data = get_full_data_by_cid(cid)
full_data_json = json.loads(full_data)
safety_section = get_content_by_sec_head(full_data_json, sec_head = "Chemical Safety")
```

1. Get CID from SMILES

```
prolog = 'https://pubchem.ncbi.nlm.nih.gov/rest/pug'
def get_cid_by_smiles(sm, prolog = "https://pubchem.ncbi.nlm.nih.gov/rest/pug"):

    """
    Get CID first
    """
    get_cid_part_url = "/compound/smiles/cids/txt"
    url = prolog + get_cid_part_url
    struct = {'smiles': sm}
    res = requests.get(url, params = struct)
    cid = res.text
    return cid
```

2. Pull full data record

```
def get_full_data_by_cid(cid, prolog = "https://pubchem.ncbi.nlm.nih.gov/rest/pug_view/data/compound/", form = "/JSON"):
    url = "".join([prolog, str(cid).rstrip(), form])
#     print(url)
    res = requests.get(url)
    return res.text
```

3. Get content in target section

```
def get_content_by_sec_head(full_data_json, sec_head = "Chemical Safety"):
    if "Record" in full_data_json.keys():
        found_item = 0
        for item in full_data_json["Record"]["Section"]:
            if "TOCHeading" in item.keys():
                if item["TOCHeading"] == sec_head:
                    return(item)
                    found_item = 1
            else:
                return -1
        if found_item == 0:
            return -1
    else:
        return -1
```

4. Extract information

```
def get_safety_kw_from_safety_section(safety_section):
    if safety_section != -1:
        if "Information" in safety_section.keys():
            safety_info_list = safety_section["Information"]
            all_safety_value_dict = safety_info_list[0]["Value"]

            safety_kw_list = []
            if "StringWithMarkup" in all_safety_value_dict.keys():
                for kw_dict in all_safety_value_dict["StringWithMarkup"][0]["Markup"]:
                    safety_kw_list.append(kw_dict["Extra"])
                return safety_kw_list
            else:
                return -1
        else:
            return -1
    else:
        return -1
```

Scheme S 8 Example workflow for extracting safety keywords from PubChem. Same workflow is transferrable to other accessible features on PubChem.

In another example, if users need to extract the boiling point of a solvent, users can specify "*TOCHeading*" as "*Boiling Point*", followed by visiting the "*Value*" key in the "*Information*" section, which is expected to return "120" (Scheme S 9).

```
                "TOCHeading": "Boiling Point",
                "Description": "The temperature at which this compound changes state from liquid to gas at a given atmospheri
c pressure.",
                "URL": "https://chem.libretexts.org/Bookshelves/Organic_Chemistry/Organic_Chemistry_Lab_Techniques_(Nichols)/
06%3A_Miscellaneous_Techniques/6.02%3A_Boiling_Point",
                "Information": [
                  {
                    "ReferenceNumber": 14,
                    "Reference": [
                      "MSDS"
                    ],
                    "Value": {
                      "Number": [
                        120
                      ],
                      "Unit": "°C"
                    }
```


Scheme S 9 A snapshot of the "Boiling Point" section of PubChem.

S1.2.2 The chemical information filtering branch

Requirements and resources:

Packages required in this branch include *pandas*, *rdkit.Chem.Fragments* and *rdkit.Chem.MACCSkeys*.

A template Python script and a sample notebook are provided in "*solv_pred_pre_filt_FG.py*" and "*candidate_prepare_by_FG.ipynb*", showing how to filter solvent candidates by functional groups and substructures.

In the template script, the MACCS key digits of ten chemistry-sensitive solvent features have been complied (Table S 3). These patterns are defined in the "*FIND_STRUCT_BY_FP*" dictionary as shown in Scheme S 10.

Table S 3 Patterns used for the functional group filtering and corresponding MACCS key fingerprint digits.

Pattern	MACCS key digit
Contain Cl element	103
Contain F element	42
Contain Br element	46
Contain P element	29
Contain primary amine group	84
Contain secondary amine group	151
Contain hydroxyl group	139
Is aromatic	162
Contain carbonyl group	154
Contain sulfinyl group	60

```
FIND_STRUCT_BY_FP = {
    "Cl" : 103,
    "F" : 42,
    "Br" : 46,
    "P" : 29,
    "NH2" : 84,
    "NH" : 151,
    "OH" : 139,
    "Aromatic" : 162,
    "C=O" : 154,
    "S=O" : 60
}
```

Scheme S 10 Loading MACCS key digits of interest.

In addition to MACCS keys, the *RDKit* methods of 44 substructures (Table S 4) that commonly existing in solvents are defined in the "*FIND_GROUP*" dictionary (Scheme S 11). The substructures are visualized in Scheme S 12

Table S 4 Patterns used for the substructure filtering in SolvPred and corresponding RDKit methods.

Pattern	RDKit method
Aliphatic_carboxylic_acids	Fragments.fr_Al_COO
Aromatic_carboxylic_acids	Fragments.fr_Ar_COO
General_carboxylic_acids	Fragments.fr_COO
Aliphatic_OH	Fragments.fr_Al_OH
Aromatic_OH	Fragments.fr_Ar_OH
Aromatic_N	Fragments.fr_Ar_N
Aromatic_amines	Fragments.fr_Ar_NH
Tertiary_amines	Fragments.fr_NH0
Secondary_amines	Fragments.fr_NH1
Primary_amines	Fragments.fr_NH2
Hydroxylamine	Fragments.fr_N_O
Amides	Fragments.fr_amide
Imides	Fragments.fr_imide
Amidine	Fragments.fr_amidine
Aniline	Fragments.fr_aniline
Thio	Fragments.fr_SH
Aldehydes	Fragments.fr_aldehyde
Alkyl_carbamate	Fragments.fr_alkyl_carbamate
Alkyl_halide	Fragments.fr_alkyl_halide
Halogens	Fragments.fr_halogen
Aryl_methyl	Fragments.fr_aryl_methyl
Benzene_rings	Fragments.fr_benzene
Ester	Fragments.fr_ester
Ether	Fragments.fr_ether
Furan_rings	Fragments.fr_furan
Imidazole_rings	Fragments.fr_imidazole
Ketones	Fragments.fr_ketone
Lactones	Fragments.fr_lactone
Methoxy	Fragments.fr_methoxy

Nitriles	Fragments.fr_nitrile
Nitros	Fragments.fr_nitro
Nitro_benzene_rings	Fragments.fr_nitro_arom
Oxazole_rings	Fragments.fr_oxazole
Phenols	Fragments.fr_phenol
P_acids	Fragments.fr_phos_acid
P_esters	Fragments.fr_phos_ester
Primary_amides	Fragments.fr_priamide
Pyridine_rings	Fragments.fr_pyridine
Sulfide	Fragments.fr_sulfide
Sulfonamides	Fragments.fr_sulfonamd
Sulfone	Fragments.fr_sulfone
Thiazole_rings	Fragments.fr_thiazole
Linear_alkanes_over_4	Fragments.fr_unbrch_alkane
Urea	Fragments.fr_urea

```
FIND_GROUP = {
    "Aliphatic_carboxylic_acids":Fragments.fr_Al_COO,
    "Aromatic_carboxylic_acids" : Fragments.fr_Ar_COO,
    "General_carboxylic_acids": Fragments.fr_COO,
    "Aliphatic_OH":Fragments.fr_Al_OH,
    "Aromatic_OH" : Fragments.fr_Ar_OH,
    "Aromatic_N" : Fragments.fr_Ar_N,
    "Aromatic_amines" : Fragments.fr_Ar_NH,
    "Tertiary_amines" : Fragments.fr_NH0,
    "Secondary_amines" : Fragments.fr_NH1,
    "Primary_amines" : Fragments.fr_NH2,
    "Hydroxylamine" : Fragments.fr_N_O,
    "Amides" : Fragments.fr_amide,
    "Imides" : Fragments.fr_imide,
    "Amidine" : Fragments.fr_amidine,
    "Aniline" : Fragments.fr_aniline,
    "Thio" : Fragments.fr_SH,
    "Aldehydes" : Fragments.fr_aldehyde,
    "Alkyl_carbamate" : Fragments.fr_alkyl_carbamate,
    "Alkyl_halide" : Fragments.fr_alkyl_halide,
    "Halogens" : Fragments.fr_halogen,
    "Aryl_methyl" : Fragments.fr_aryl_methyl,
    "Benzene_rings" : Fragments.fr_benzene,
    "Ester" : Fragments.fr_ester,
    "Ether" : Fragments.fr_ether,
    "Furan_rings" : Fragments.fr_furan,
    "Imidazole_rings" : Fragments.fr_imidazole,
    "Ketones" : Fragments.fr_ketone,
    "Lactones" : Fragments.fr_lactone,
    "Methoxy" : Fragments.fr_methoxy,
    "Nitriles" : Fragments.fr_nitrile,
    "Nitros" : Fragments.fr_nitro,
    "Nitro_benzene_rings" : Fragments.fr_nitro_arom,
    "Oxazole_rings" : Fragments.fr_oxazole,
    "Phenols" : Fragments.fr_phenol,
    "P_acids" : Fragments.fr_phos_acid,
    "P_esters" : Fragments.fr_phos_ester,
    "Primary_amides" : Fragments.fr_priamide,
    "Pyridine_rings" : Fragments.fr_pyridine,
    "Sulfide" : Fragments.fr_sulfide,
    "Sulfonamides" : Fragments.fr_sulfonamd,
    "Sulfone" : Fragments.fr_sulfone,
    "Thiazole_rings" : Fragments.fr_thiazole,
    "Linear_alkanes_over_4" : Fragments.fr_unbrch_alkane,
    "Urea" : Fragments.fr_urea
}
```

Scheme S 11 Substructures used for pre-filtering of candidates with the Fragments method implemented in RDKit.

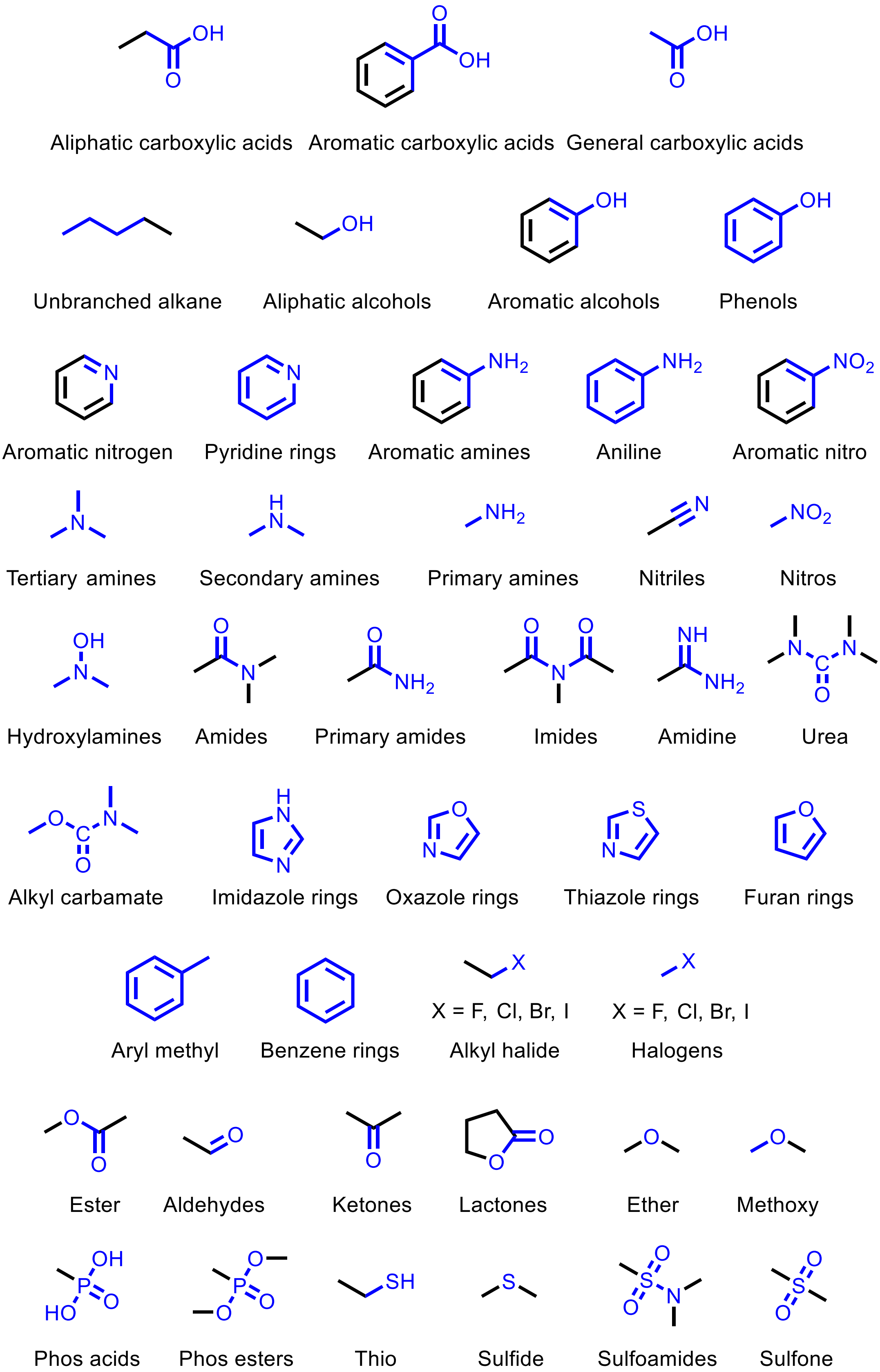

Scheme S 12 Example chemical schemes of substructures used for candidate filtering.

To check if a substructure or a functional group exists in an input SMILES string, users can run the "*find_FG*" function (Scheme S 13). The SMILES is loaded into the "*sm*" argument,

while the name of the structure is loaded into the "*FG*" argument. If a pattern exists, this function returns "*True*". The number of functional groups, if pre-defined in the "*FING_GROUP*" dictionary, can be calculated as a reference (Scheme S 14).

```
def find_FG(sm, FG = "Aliphatic_OH"):
    mol = Chem.MolFromSmiles(sm)

    if FG not in FIND_STRUCT_BY_FP.keys() and (FG not in FIND_GROUP.keys()):
        raise ValueError(f"Cannot find {FG}. Please check FIND_GROUP or FIND_STRUCT_BY_FP for all avilable groups.")

    if FG in FIND_STRUCT_BY_FP.keys():
        find_FG_res = find_struct_from_fp(mol, FG)
        return find_FG_res

    else:
        n_FG = FIND_GROUP[FG](mol)
        print(f"Number of {FG} in this molecule is: {n_FG}")

        if n_FG != 0:
            find_FG_res = {"FG" : FG, "FG_exist" : "True"}
        else:
            find_FG_res = {"FG" : FG, "FG_exist" : "False"}
    return find_FG_res
```

Scheme S 13 The "finding functional group" function.

```
sm = "CC1=CC=CC=C1"
FG = "Aromatic"
find_FG(sm, FG)

{'FG': 'Aromatic', 'FG_exist': 'True'}
```

```
sm = "CCCCN"
FG = "NH2"
find_FG(sm, FG)

{'FG': 'NH2', 'FG_exist': 'True'}
```

```
sm = "C(COCCOCCO)O"
FG = "Aliphatic_OH"
find_FG(sm, FG)

Number of Aliphatic_OH in this molecule is: 2

{'FG': 'Aliphatic_OH', 'FG_exist': 'True'}
```

```
sm = "CCCCN"
FG = "Primary_amines"
find_FG(sm, FG)

Number of Primary_amines in this molecule is: 1

{'FG': 'Primary_amines', 'FG_exist': 'True'}
```

Scheme S 14 Example output of finding different substructures from a given SMILES.

An example of using the "*solv_pred_pre_filt_FG.py*" script to run a functional group filtering task is given below (Scheme S 15):

Users need to first pass the initial candidate list in *json* format to the "*db_json_name*" variable, then specify the MACCS digit of the target functional group to filter - exemplified for "*alcohol*" in this case. This functional group is fed to the "*FG_to_chk*" variable. Existence of the target functional group is checked for each candidate.

Depending on whether a functional group needs to be kept or not, users can define a container list to collect valid results. For example, when solvents without hydroxyl groups are intended to be kept, a "*non_OH_subset*" list can be created to collect results that return "*False*" in the hydroxyl group checking process. In contrast, if solvents containing hydroxyl groups are intended to be kept, users can create an "*OH_subset*" list to collect results that return "*True*" in the same checking procedure. Valid results are then exported as both *csv* and *json* files for downstream processing.

1. Load input candidate set

```
db_json_name = "db_solv_pred_v2.json"
db_df = pd.read_json(db_json_name)
```

2. Specify functional group (FG) to check

```
alcohol = {
    "MACCS_dig" : 139,
    "FG" : "OH"
}
```

```
FG_to_chk = alcohol
```

3. Check existence of target FG in each candidate

```
# OH_subset = []
non_OH_subset = []

for entry in db_dict:
    entry_sm = entry["SMILES"]
    if type(entry_sm) is str:
        entry_MACCS = get_MACCS_from_sm(entry_sm)
        FG_dig = FG_to_chk["MACCS_dig"]
        if entry_MACCS[FG_dig] == str(1):
            entry[FG_to_chk["FG"]] = "True"
            # OH_subset.append(entry)
        else:
            entry[FG_to_chk["FG"]] = "False"
            non_OH_subset.append(entry)
    else:
        entry[FG_to_chk["FG"]] = -1
```

4. Results filtering and export

```
non_OH_db = pd.DataFrame.from_dict(data = non_OH_subset)
non_OH_db.to_csv("non_OH_subset.csv")
non_OH_cand = pd.DataFrame()
non_OH_cand["CAS"] = non_OH_db["CAS"]
non_OH_cand["Solvent"] = non_OH_db["Name"]
non_OH_cand.to_json("non_OH_candidates.json", orient = "records")
```

Scheme S 15 Example implementation of filtering of solvents that do not contain hydroxyl groups.

S1.2.3 Merging multiple features

To merge multiple features, users can run "*Merge_candidate_set.ipynb*". Results filtered from parent features are loaded as "*cand_1_filename*" and "*cand_2_filename*", respectively. The output shows the intersection of both input candidate sets. The output is saved as *csv* and *json* formats for future uses (Scheme S 16).

```
cand_1_filename = "non_chlorinated_candidates.csv"
cand_2_filename = "safe_green_candidates.csv"
```

```
intersect_cand(cand_1_filename, cand_2_filename)
```

```
Number of candidates in set 1 is: 218.
Number of candidates in set 2 is: 138.
Number of candidates in intersection is: 127.
Merging non_chlorinated and safe_green candidates...
saving results...
non_chlorinated_safe_green_candidates.csv saved.
non_chlorinated_safe_green_candidates.json saved.
```

Scheme S 16 Illustration of merging two candidate sets filtered by different parent features.

Using this method, a list of subsets with distinct structural features have been provided in the "*cand_subsets*" folder on GitHub repository, as listed in Table S 5. These subsets are ready to use as an alternative candidate list when running the *SolvPred* main program.

Table S 5 Example subsets filtered by distinct features in the "cand_subsets" folder.

Filename and number of entries (in brackets)	Explanation
solv_candidate_full (**248**)[a]	The full solvent set minus one solvent (naphtha) that does not carry a SMILES representation – this is because naphtha is a hydrocarbon mixture.
non_chlorinated_candidates (**217**)[a]	A subset of non-chlorinated solvents
safe_green_candidates (**137**) [a]	A subset of safe and green solvents
non_fluorinated_candidates (**236**)	A subset of non-fluorinated solvents
OH_candidates (**73**)	A subset of solvents with hydroxyl groups
non_OH_candidates (**175**)	A subset of solvents without any hydroxyl groups
non_chlorinated_safe_green_candidates (**127**)	A secondary subset merged from the non-chlorinated solvent subset and the safe and green solvent subset
non_OH_safe_green_candidates (**81**)	A secondary subset merged from the no-hydroxyl group solvent subset and the safe and green solvent subset
non_OH_non_chlorinated_candidates (**135**)	A secondary subset merged from the non-chlorinated solvent subset and the no-hydroxyl group solvent subset
OH_non_chlorinated_candidates (**72**)	A secondary subset merged from the non-chlorinated solvent subset and the hydroxyl group solvent subset
OH_safe_green_candidates (**56**)	A secondary subset merged from the non-chlorinated solvent subset and the safe and green solvent subset
non_OH_non_chlorinated_safe_green_candidates (**71**)	A tertiary subset merged from the non-chlorinated solvent subset, the no-hydroxyl group solvent

	subset, and the safe and green solvent subset.
OH_non_chlorinated_safe_green_candidates (**55**)	A tertiary subset merged from the non-chlorinated solvent subset, the hydroxyl group solvent subset, and the safe and green solvent subset.

[a] One solvent (naphtha) does not have SMILES information, therefore gets excluded from the filtering workflow as it can fail in parsing CID or RDKit descriptors. This solvent is kept in the built-in HSP database and this would not affect the inverse calculation based solely on HSPs.

S1.3 User interfaces and working examples

SolvPred provides both a command line-based user interface (S1.3.1) and an executable program with graphical user interface (S1.3.2). Both interfaces are based on the same core algorithm. Users can select the most convenient way, depending on their coding expertise and development needs.

S1.3.1 Command line-based user interface

To load this module, users need to run the main program, *solv_pred_main.py*, in terminal using the following command:

```
python solv_pred_main.py
```

Users can then expect to see the introduction page shown in Scheme S 17. This step automatically loads the built-in database and the default candidate list. Using the keyboard operations [v] and [q], users can visualize the current candidate list or quit the programme, respectively.

```
2023-01-22  10:15:54
SolvPredictor v 2.0
 (License: GPL-3.0)

Loading database and default candidate list...
Done.

Please follow the instruction to continue:
=========================
Step 1: Generate solvent candidate list:
 You can select to use the default list (recommend) or
manually input the CAS No. for each solvent.

Please select the method to construct solvent candidate
list:
 [d] - default (recommend - the default list can still
be edited in the following steps)
 [m] - manual
 [v] - visualise default solvent list
 [q] - quit
```

Scheme S 17 The introduction page of SolvPred.

The candidate list uses the format of "*CAS*" and "*Solvent*", loaded as a *json* file. An example default candidate list is provided in the "*default_solv_candidate.json*" file in the main directory. Users can modify the candidate solvents prior to loading by directly editing the *json* file, or by editing its excel counterpart, "*default_solv_candidate.xlsx*", followed by converting the files to the corresponding *json* format.

Where necessary, a candidate list with a different file name can be passed to the main program, by editing the source code of "*solv_pred_main.py*" for the function named "*solv_pred_main*", where the first argument, "*db*", is the name of database, and the second argument, "*default_candidate*", is the name of the candidate list. If users wish to upload a custom database or candidate lists, they can modify these arguments accordingly.

```
solv_pred_main(db: str = 'db_solv_pred_v2.json', default_candidate: str = 'solv_candidate_full.json')
```

For example, the *json* files generated by the extendible solvent candidate filtering module (Table S 5) are readily compatible with the candidate loading function in the main program. Users can save a copy of the candidate list in the same working directory of *solv_pred_main.py*, followed by modifying the "*default_candidate*" argument using the corresponding filename.

The example default candidate list includes common solvents that are typically available in research laboratories, with their HSPs widely distributed in the Hansen space (Scheme S 18).

```
v
The default solvent candidates include the following solvents:

{'CAS': '121-44-8', 'Solvent': 'Triethylamine'}
{'CAS': '109-66-0', 'Solvent': 'Pentane'}
{'CAS': '1330-20-7', 'Solvent': 'Xylene'}
{'CAS': '60-29-7', 'Solvent': ‘Diethyl ether'}
{'CAS': '109-99-9', 'Solvent': 'Tetrahydrofuran'}
{'CAS': '110-54-3', 'Solvent': 'Hexane'}
{'CAS': '79-01-6', 'Solvent': 'Trichloroethylene'}
{'CAS': '142-82-5', 'Solvent': 'Heptane'}
{'CAS': '110-82-7', 'Solvent': 'Cyclohexane'}
{'CAS': '108-88-3', 'Solvent': 'toluene'}
{'CAS': '71-43-2', 'Solvent': 'benzene'}
{'CAS': '108-90-7', 'Solvent': 'chlorobenzene'}
{'CAS': '75-09-2', 'Solvent': 'methylene dichloride'}
{'CAS': '75-65-0', 'Solvent': 't-butyl alcohol'}
{'CAS': '67-63-0', 'Solvent': '2-propanol'}
{'CAS': '141-78-6', 'Solvent': 'ethyl acetate'}
{'CAS': '123-91-1', 'Solvent': 'dioxane'}
{'CAS': '71-23-8', 'Solvent': '1-propanol'}
{'CAS': '67-64-1', 'Solvent': 'acetone'}
{'CAS': '110-86-1', 'Solvent': 'pyridine'}
{'CAS': '64-17-5', 'Solvent': 'ethyl alcohol'}
{'CAS': '68-12-2', 'Solvent': 'dimethylformamide'}
{'CAS': '67-56-1', 'Solvent': 'methanol'}
{'CAS': '108-95-2', 'Solvent': 'phenol'}
{'CAS': '75-05-8', 'Solvent': 'acetonitrile'}
{'CAS': '67-68-5', 'Solvent': 'dimethylsulfoxide'}
{'CAS': '74-87-3', 'Solvent': 'Methylcyclohexane'}
{'CAS': '67-66-3', 'Solvent': 'chloroform'}
{'CAS': '616-47-7', 'Solvent': 'NMI'}
{'CAS': '108-32-7', 'Solvent': 'Propylene carbonate'}
{'CAS': '91-22-5', 'Solvent': 'Quinoline'}
{'CAS': '108-39-4', 'Solvent': 'm-Cresol'}
{'CAS': '108-67-8', 'Solvent': 'Mesitylene'}
{'CAS': '872-50-4', 'Solvent': 'N-Methyl-2-pyrrolidone'}
{'CAS': '56-81-5', 'Solvent': 'Glycerol'}
{'CAS': '127-19-5', 'Solvent': 'N,N-dimethylacetamide'}
Please select the method to construct solvent candidate list:
 [d] - default (recommend - the default list can still be edited in the
following steps)
 [m] - manual
 [v] - visualise default solvent list
 [q] - quit
```

Scheme S 18 The example default candidate list.

Using keyboard operation [d], users can load the default candidate list, which can be modified further by adding or removing solvents. An example is shown in Scheme S 19 to remove a solvent with CAS No. 108-39-4 from the existing candidate pool.

```
d
You have selected to use the default solvent candidate list.
Solvent candidate list has been generated successfully.
Do you want to remove any solvent?

[y/n]: y

Please enter the CAS No. of solvent candidate 1 : 108-39-4
Please enter the CAS No. of solvent candidate 2 :
Have all the solvent candidates been added?
[y/n]: y

Do you want to remove any solvent?

[y/n]: n
Continue?
[y/n]: y
The following solvents will be removed:
['108-39-4']
Checking if any solvent is not on the candidate list...
Done.
 Checking if any solvent is not in the database...
Done.

Submit?
[y/n]: y
```

Scheme S 19 An example of loading default candidate list following by removing a solvent from the pool.

Users can also input solvent candidates manually, using keyboard operation [m], followed by entering the CAS number of each candidate. An example is shown in Scheme S 20.

```
m
Please submit CAS No. of solvents to be considered as candidates. Add one solvent at one time.
Press [enter] to continue.
Please enter the CAS No. of solvent candidate 1 : 121-44-8
Please enter the CAS No. of solvent candidate 2 : 109-66-0
Please enter the CAS No. of solvent candidate 3 : 109-99-9
Please enter the CAS No. of solvent candidate 4 :
Have all the solvent candidates been added?
[y/n]: y
Solvent candidate list has been generated successfully.
Do you want to remove any solvent?

[y/n]: n
Continue?
[y/n]: y
```

Scheme S 20 Manual mode [m] of loading solvent candidates.

Before confirming the final candidates, both manual and automated modes will lead to the same candidate-editing step shown in Scheme S 21. Using keyboard operation [a] directs users to add CAS No.; keyboard operation [rm] allows users to remove CAS No. of a specific candidate; keyboard operation [v] is for visualizing the current candidate list; keyboard operation [s] leads to the submission page and to the next step.

```
Submit?
[y/n]: n
Please select one of the following options:
[a] - add CAS
[rm] - remove CAS
[v] - visualise current candidate list
[s] - submit
[q] - quit
```

Scheme S 21 The candidate editing and submission step.

The confirmed candidates will be presented to users as shown in Scheme S 22.

```
The following solvents will be considered as candidates:
['67-66-3', '108-95-2', '108-32-7', '121-44-8', '110-86-1', '74-
87-3', '616-47-7', '1330-20-7', '75-05-8', '67-68-5', '141-78-6',
'75-09-2', '108-90-7', '142-82-5', '67-63-0', '60-29-7', '123-91-
1', '79-01-6', '68-12-2', '110-54-3', '109-99-9', '75-65-0', '872-
50-4', '127-19-5', '56-81-5', '108-67-8', '67-64-1', '110-82-7',
'71-23-8', '109-66-0', '91-22-5', '67-56-1', '108-88-3', '64-17-
5', '71-43-2']
Candidate list has been successfully generated:
['Chloroform', 'Phenol', 'Propylene carbonate', 'Triethylamine',
'Pyridine', 'Methyl chloride', '1-Methylimidazole', 'm-Xylene',
'Acetonitrile', 'Dimethyl sulfoxide', 'Ethyl acetate', 'Methylene
dichloride', 'Chlorobenzene', 'Heptane', '2-Propanol', 'Diethyl
ether', '1,4-Dioxane', 'Trichloroethylene', 'Dimethylformamide',
'Hexane', 'Tetrahydrofuran', 't-Butyl Alcohol', 'Methyl-2-
pyrrolidone', 'N,N-dimethylacetamide', 'Glycerol', 'Mesitylene',
'Acetone', 'Cyclohexane', '1-Propanol', 'Pentane', 'Quinoline',
'Methanol', 'Toluene', 'Ethanol', 'Benzene']
```

Scheme S 22 Confirmation page of solvent candidates.

SolvPred will then direct users to specify parameters for co-solvent calculations (Scheme S 23). Using keyboard operation [t] leads to the temperature correction; using keyboard operation [c] continues the calculation using 25 ℃ as the working temperature.

```
=========================

Step 2: Specify parameters.
 Please follow the instruction to specify the
 - Maximum number of solvents (n) to be included in each
combination (default = 2);
 - Highest acceptable error of HSP (tol_err) of the predicted
combination (default = 0.5);
 - Lowest acceptable concentration (tol_conc) of each predicted
solvent component (default = 0.01).
 - Target HSP (target D, P, H).
 Temperature (default = 25 degree C).

[t] - set a different temperature
[c] - continue as room temperature (25 degree c).
[q] - quit
```

Scheme S 23 Home page of specifying parameters for co-solvents calculation.

An example of applying a temperature correction is shown in Scheme S 24. The target temperature is specified in degrees Celsius. *SolvPred* will correct all HSPs in the database to the HSPs at the specified temperature, according to the thermal expansion relationship with HSPs at room temperature as explained in S1.1.2.

```
t
Please enter the target temperature (in degree C): 60
Temperature has been set as: 60
Applying temperature correction for standard HSP...
Database HSP temperature correction done.
```

Scheme S 24 Applying temperature correction.

Users then need to specify the number of solvents, target HSPs, tolerance of error and redundant solvent concentration. *SolvPred* offers a default mode [d] and a manual mode [m] to complete this step (Scheme S 25).

```
Set parameters:
[d] - use default settings for all parameters.
[m] - manually set parameters.
```

Scheme S 25 Home page of specifying parameters for the number of solvents, target HSPs, error tolerance and redundant solvent threshold.

By default, the number of solvents (n), error tolerance of δ_D (*tol_err_d*), error tolerance of δ_P (*tol_err_p*), error tolerance of δ_H (*tol_err_h*), and tolerance of concentration (*tol_conc*) are 2, 0.5 $MPa^{1/2}$, 0.5 $MPa^{1/2}$, 0.5 $MPa^{1/2}$, 0.01, respectively. This allows for the prediction of binary solvent mixtures with prediction errors above 0.5 for each HSP, and solvents below 1% in a mixture, to be omitted.

```
d
Default parameters will be applied.
[n, tol_err_d, tol_err_p, tol_err_h, tol_conc] : [2, 0.5, 0.5,
0.5, 0.01]
```

Scheme S 26 Default parameters of the number of solvents (n), error tolerance of δ_D (tol_err_d), error tolerance of δ_P (tol_err_p), error tolerance of δ_H (tol_err_h), and tolerance of concentration (tol_conc).

Alternatively, users can specify the above parameters *via* manual mode as shown in Scheme S 27. The number of solvents needs to be a positive integer above or equal to 2 (Scheme S 27a). The error tolerance of each HSP dimension can be specified separately (Scheme S 27b), with each unit as $MPa^{1/2}$. The tolerance of concentration is a float ranging between 0 to 1, corresponding to 0% and 100%, respectively (Scheme S 27c). All parameters are summarized for users for confirmation before proceeding to the next stage (Scheme S 27d).

(a)

```
m
Please specify parameters:
n is the maximum number of solvents involved in each prediction.
Must be a positive integer >= 2. (recommend to be 2 or 3)
Please specify n: 2
```

(b)

```
tolerance of error is the highest acceptable absolute error of HSP
from the predicted solvent mixture.
 Must be a positive float.

Please specify tolerance of error for dispersion term (tol_err_D):
0.5
Please specify tolerance of error for dipolar term (tol_err_P):
0.2
Please specify tolerance of error for hydrogen bond term
(tol_err_H): 0.3
```

(c)

```
lowest acceptable concentration is the threshold of predicted
solvent concentration below which will be filtered out.
Must be a float between 0 and 1.
Please specify lowest acceptable concentration: 0.02
```

(d)

```
n, tol_err, tol_conc have been specified as:
[2, 0.5, 0.2, 0.3, 0.02]
Continue?
[y/n]:
```

Scheme S 27 Manual mode of parameter specification for (a) the number of solvents, (b) tolerance of error for each HSP dimension, (c) tolerance of redundant solvent concentration. (d) The confirmation step of all parameters.

Users then need to specify the target HSPs following the instruction shown in Scheme S 28. Each HSP has units of $MPa^{1/2}$.

```
target d, p, h are target HSPs to be achieved.
 Must be non-negative float.
Please specify the target of dispersion term (target D): 15
Please specify the target of dipolar term (target P): 2
Please specify the target of hydrogen bond term (target H): 3
target d, target p, target h have been specified as:
['15', '2', '3']
Continue?
[y/n]: y
```

Scheme S 28 Specifying target HSPs step.

According to the target HSPs, *SolvPred* will do a feasibility check, to ensure that the target HSPs are achievable. Given all concentrations of co-solvents must be positive, the target HSPs can only be achieved in the region connected by HSPs of co-solvent candidates. In the case when an unrealistic target is provided, a correction message will be presented to users, redirecting users to either select a reasonable target based on the candidates available, or adding more candidates that cover a broader range of HSPs.

For example, in the case shown in Scheme S 29, an overly high δ_D (36 $MPa^{1/2}$) has been specified. This value falls beyond all possible δ_D that can be achieved by combining any solvent in the candidate set, which only covers the region between 14.06 $MPa^{1/2}$ to 19.40 $MPa^{1/2}$ in this specific case. This target δ_D will be flagged, with the suggestion interval of target δ_D presented for reference.

```
target d, p, h are target HSPs to be achieved.
 Must be non-negative float.
Please specify the target of dispersion term (target D): 36
Please specify the target of dipolar term (target P): 2
Please specify the target of hydrogen bond term (target H): 3
target d, target p, target h have been specified as:
['36', '2', '3']
Continue?
[y/n]: y

Validating parameters...
Validating n...
Validation of n is done.
Validating target HSP...
Target D is not achievable.
Target D must be in the interval of 14.062668321565182 to
19.396783891814046
Target P validation done.
Target H validation done.
```

Scheme S 29 An example of when target HSPs fall beyond the predictable region connected by all candidates.

SolvPred will direct users to the correction part, where users can either add more solvents to the candidate pool to expand the predictable HSPs interval (using keyboard operation [a]), or reset the target HSPs to the recommended interval based on the existing candidates (using keyboard operation [r]).

In the example given here, adding solvents cannot improve the situation because no solvents in the database have a large-enough δ_D compared to the target. Restricting the target δ_D to the region between 14.06 MPa$^{1/2}$ to 19.40 MPa$^{1/2}$ is more feasible. As demonstrated in Scheme S 30, the new target is validated to be achievable using the existing candidate set, successfully proceeding to the next stage.

```
Please choose to reset the required parameters or add more solvent
candidates.
 [r] - reset
 [a] - add
 [q] - quit

r
Please reset target HSP.
target d, p, h are target HSPs to be achieved.
 Must be non-negative float.
Please specify the target of dispersion term (target D): 18
Please specify the target of dipolar term (target P): 3
Please specify the target of hydrogen bond term (target H): 2
target d, target p, target h have been specified as:
['18', '3', '2']
Continue?
[y/n]: y

Validating parameters...
Validating n...
Validation of n is done.
Validating target HSP...
Target D validation done.
Target P validation done.
Target H validation done.
Parameter selection done.
```

Scheme S 30 An example of correcting target HSPs to a feasible range.

With all parameters ready, the calculation will begin. Successful completion will yield the full calculation log file (“*calc_log_bsc_chk_time.json*”), valid results log file (“*calc_log_vld_time.txt*”) and results that have passed all filtering steps (“*log_success_time.txt*”). All metadata are saved in these documents for users’ reference.

Once the calculation is complete, users will be asked whether to proceed with the optional advanced filtering step (Scheme S 31). The advanced filtering step yields a full calculation log file (“*calc_log_adv_all_time.txt*”) and an organized output report (“*log_adv_filt_success_time.txt*”). The file path of this output is presented to users at the end of the program.

```
Generating perturbated target HSP matrix...
Done.

2023-01-22  10:15:54
SolvPredictor v 2.0
 (License: GPL-3.0)

=========================
Step 4: Advanced filtration (optional)
 This step will further include the consideration of solvent
properties according to the condition set up.
 In this version, miscibility and boiling point will be evaluated.
 Data are based on PubChem.
 Please note, there could be some limitation of this function due
to data availability. Check README for more discussion.

Continue advanced filtration?

[y/n]:y
2023-01-23  07:50:28
SolvPredictor v 2.0
 (License: GPL-3.0)

Please check:
C:\Users\...\hsp_toolkit_solv_pred_v_2.0\log\log_adv_filt_success_
01232023075028.txt for calculation log.
```

Scheme S 31 The interface of the calculation has completed and the advanced filtering step.

In the output report, the first section summarizes the input parameters, candidates, and the path of the calculation log (Scheme S 32).

```
==============================
SolvPredictor v 2.0
 (License: GPL-3.0)

10:15:54
2023-01-22
==============================

Target D /MPa^(1/2):
18

Target P /MPa^(1/2):
3

Target H /MPa^(1/2):
2

Temperature /degree c:
60.0

Number of candidates in each combination (n):
2

Tolerance of error for D /MPa^(1/2):
0.5

Tolerance of error for P /MPa^(1/2):
0.2

Tolerance of error for H /MPa^(1/2):
0.3

Lowest concentration limit:
0.02

Candidate cas:
['67-66-3', '108-95-2', '108-32-7', '121-44-8', '110-86-1', '74-87-3', '616-47-7', '1330-
20-7', '75-05-8', '67-68-5', '141-78-6', '75-09-2', '108-90-7', '142-82-5', '67-63-0',
'60-29-7', '123-91-1', '79-01-6', '68-12-2', '110-54-3', '109-99-9', '75-65-0', '872-50-
4', '127-19-5', '56-81-5', '108-67-8', '67-64-1', '110-82-7', '71-23-8', '109-66-0', '91-
22-5', '67-56-1', '108-88-3', '64-17-5', '71-43-2']

Candidate solvents:
['Chloroform', 'Phenol', 'Propylene carbonate', 'Triethylamine', 'Pyridine', 'Methyl
chloride', '1-Methylimidazole', 'm-Xylene', 'Acetonitrile', 'Dimethyl sulfoxide', 'Ethyl
acetate', 'Methylene dichloride', 'Chlorobenzene', 'Heptane', '2-Propanol', 'Diethyl
ether', '1,4-Dioxane', 'Trichloroethylene', 'Dimethylformamide', 'Hexane',
'Tetrahydrofuran', 't-Butyl Alcohol', 'Methyl-2-pyrrolidone', 'N,N-dimethylacetamide',
'Glycerol', 'Mesitylene', 'Acetone', 'Cyclohexane', '1-Propanol', 'Pentane', 'Quinoline',
'Methanol', 'Toluene', 'Ethanol', 'Benzene']

Calculation log path:
C:\Users\...\hsp_toolkit_solv_pred_v_2.0\log\calc_log_bsc_chk_01222023101554.json
```

Scheme S 32 The first section of the output report, including the target HSPs, error tolerance, concentration tolerance, solvent candidates and the file path of the log of calculation.

The second section summarizes the results, including the co-solvents, concentrations, calculated HSPs, along with the error of prediction. Example results obtained before applying the advanced filtering step are shown in Scheme S 33.

```
==============================
Results:
==============================

Group 1 :

solvent 1 : Propylene carbonate (108-32-7)
concentration 1 :  9.47%

solvent 2 : Toluene (108-88-3)
concentration 2 :  90.53%

calculated D /MPa^(1/2): 17.640788087782173
calculated P /MPa^(1/2): 2.9357929244636503
calculated H /MPa^(1/2): 2.0812967786641057
error of D /MPa^(1/2): -0.3592119122178268
error of P /MPa^(1/2): -0.06420707553634974
error of H /MPa^(1/2): 0.08129677866410567


********
Group 2 :

solvent 1 : Propylene carbonate (108-32-7)
concentration 1 :  16.91%

solvent 2 : Benzene (71-43-2)
concentration 2 :  83.09%

calculated D /MPa^(1/2): 18.10739983538574
calculated P /MPa^(1/2): 3.0062706660797662
calculated H /MPa^(1/2): 2.2291355259840344
error of D /MPa^(1/2): 0.10739983538573838
error of P /MPa^(1/2): 0.0062706660797662295
error of H /MPa^(1/2): 0.22913552598403442
```

Scheme S 33 The second section (the result part) of the output report. Each entry includes the co-solvent, concentrations, calculated HSPs and prediction errors.

Users can check the full calculation log file to trace back calculation details, including the results of each validation step.

The data structure of the calculation log is shown in Scheme S 34a. Here, "*idx*" stores the index of each prediction; "*cas_comb*" includes all CAS numbers of co-solvents involved in the corresponding prediction; "*err*" saves the average and standard deviation of the calculated error from target HSPs and the deviation from 1 (the normalization factor) for the last element; "*calc_hsp*" are the actual calculated HSP values; "*quality*" describes the quality of the results. This can be an error message if the results are invalid. "Validity" (*True* or *False*) summarizes the validity of the results.

An example of a statistically unstable result is shown in Scheme S 34b, where the "quality" tag indicates the results as "*UnstableResults*", and the "*validity*" is identified as "*False*". This solvent combination will be filtered accordingly.

(a)

```
[{
  "idx" : index,
  "cas_comb" : [cas_1, cas_2, ..., cas_n],
  "conc" : [[[average of conc for solvent 1], [std of conc for solvent 1]], ..., [conc info for
solvent n]],
  "err" : [[[average error of D], [std of error of D], [average error of P], [std of error of P]],
[[average error of H], [std of error of H], [average of error from 1], [std of error from 1]]],
  "calc_hsp": [[calculated D], [calculated P], [calculated H], [calculated "1"]],
  "quality": "summary of data quality, can be an error message",
  "validity": "True or False"

}]
```

(b)

```
{
  "idx": 0, "cas_comb": ["67-66-3", "108-95-2"],
  "conc": [[[1.4414674981149098], [-0.39069270146057705]], [[0.013704739565376992],
[0.012101233403607914]]],
  "err": [[[0.06547142600175924], [-0.8699042557516072], [0.2748449378482521], [0.05077479665433273]],
[[0.007840492471920472], [0.09953634088029777], [0.03144069036004137], [0.004752218301362077]]],
  "calc_hsp": [[18.063888149221956], [2.137121558765653], [2.26698643610238], [1.0507747966543328]],
  "quality": "UnstableResult",
  "validity": "False"
  }
```

Scheme S 34 (a) The data structure of the full calculation log file and (b) an example of a statistically unstable result. The error message is shown in the “quality” tag and “validity” is identified “False”.

For results that have passed the advanced filtering step, the output report further includes the boiling point and miscibility, as highlighted in Scheme S 35.

```
Group 1 :

solvent 1 : Propylene carbonate (108-32-7)
concentration 1 :  9.47%

bp /degree C: 241.6

miscibility check:
No recorded miscibility issue or no solubility record. Manual
check is recommended.

solvent 2 : Toluene (108-88-3)
concentration 2 :  90.53%

bp /degree C: 110.6

miscibility check:
No recorded miscibility issue. Manual check is recommended if
temperature has been varied.

calculated D /MPa^(1/2): 17.640788087782173
calculated P /MPa^(1/2): 2.9357929244636503
calculated H /MPa^(1/2): 2.0812967786641057
error of D /MPa^(1/2): -0.3592119122178268
error of P /MPa^(1/2): -0.06420707553634974
error of H /MPa^(1/2): 0.08129677866410567
```

Scheme S 35 Example results in the output report after passing the advanced filtering steps. The red frames highlight the boiling point and miscibility information that are used for advanced property filtering.

The full calculation log after the advanced filtering step includes the corresponding solvent properties. An example entry of the calculation log is shown in Scheme S 36. Information underlined in blue is related to miscibility validation, where the ID of each co-solvent (No.) is compared with the ID of all solvents that are recorded as immiscible listed in the “*imd_idx*” key. Any immiscible co-solvents are saved in the “*ims_solvent_in_comb*” key. If no co-solvents are found immiscible, the “*ims_validity*” key will be labelled as “*true*”. For boiling point validation, the boiling point recorded in the

"*bp*" key is compared to the working temperature. If the boiling point is above or equal to working temperature, "*bp_chk_msg*" will be recorded accordingly, with "*bp_validity*" identified as "*true*". Information related to boiling point validation is underlined in green in Scheme S 36.


```
{
  "group": 4,
  "full_detail":
  [
    {
      "CAS": "67-68-5",
      "No.": 93,
      "Name": "Dimethyl sulfoxide",
      "ims_idx": "139;81;244;47;136;240;190;106",
      "bp": 189,
      "ori_idx": 285,
      "ims_chk_msg": "No recorded miscibility issue. Manual check
is recommended if temperature has been varied.",
      "ims_solvent_in_comb": null,
      "ims_validity": true,
      "bp_chk_msg": "Above or equal to set temp",
      "bp_validity": true,
      "conc": 0.17370185847668593,
      "calc_d": 17.524490650842214,
      "calc_p": 2.8140266478430767,
      "calc_h": 2.1462982015123093,
      "err_d": -0.475509349157786,
      "err_p": -0.18597335215692334,
      "err_h": 0.14629820151230932
    },
    {
      "CAS": "108-67-8",
      "No.": 149,
      "Name": "Mesitylene",
      "ims_idx": "243",
      "bp": 164.7,
      "ori_idx": 285,
      "ims_chk_msg": "No recorded miscibility issue. Manual check
is recommended if temperature has been varied.",
      "ims_solvent_in_comb": null,
      "ims_validity": true,
      "bp_chk_msg": "Above or equal to set temp",
      "bp_validity": true,
      "conc": 0.826298141523314,
      "calc_d": 17.524490650842214,
      "calc_p": 2.8140266478430767,
      "calc_h": 2.1462982015123093,
      "err_d": -0.475509349157786,
      "err_p": -0.18597335215692334,
      "err_h": 0.14629820151230932
    }
  ]
}
```


Scheme S 36 An example entry of full calculation log of a predicted solvent combination after advanced filtering. Information underlined in blue is related to miscibility. Information underlined in green is related to boiling point.

The metadata of all calculation steps are saved as intermediate outputs for users' reference and summarized in Table S 6. The corresponding files are provided in the "*example_output*" folder in the *SolvPred* GitHub repository.

Table S 6 Output and metadata of SolvPred calculation.

Filename prefix	Explanation
calc_log_bsc_chk_ (json)	The full calculation log before advanced filtering

calc_log_vld_ (txt)	Results with validation information
log_success_ (txt)	Results that have passed all validation and error check steps
calc_log_adv_all_ (txt)	The full calculation log after advanced filtering
log_adv_filt_success_ (txt)	Results that have passed the advanced filtering steps

S1.3.2 Graphical user interface of *SolvPred*

SolvPred also includes a graphical user interface (GUI) for users who prefer an executable program.

Due to the file size limit on GitHub, the executable of this program cannot be directly uploaded on GitHub. To launch the GUI application, users need to first create the environment in the installation directory of *SolvPred App* where the repository is cloned:

```
conda env create --file environment.yml
```

Then they need to run the following line to load the executable program:

```
python run_app.py
```

This will launch the executable file "SolvPredApp.exe". A welcome page will appear (Scheme S 37).

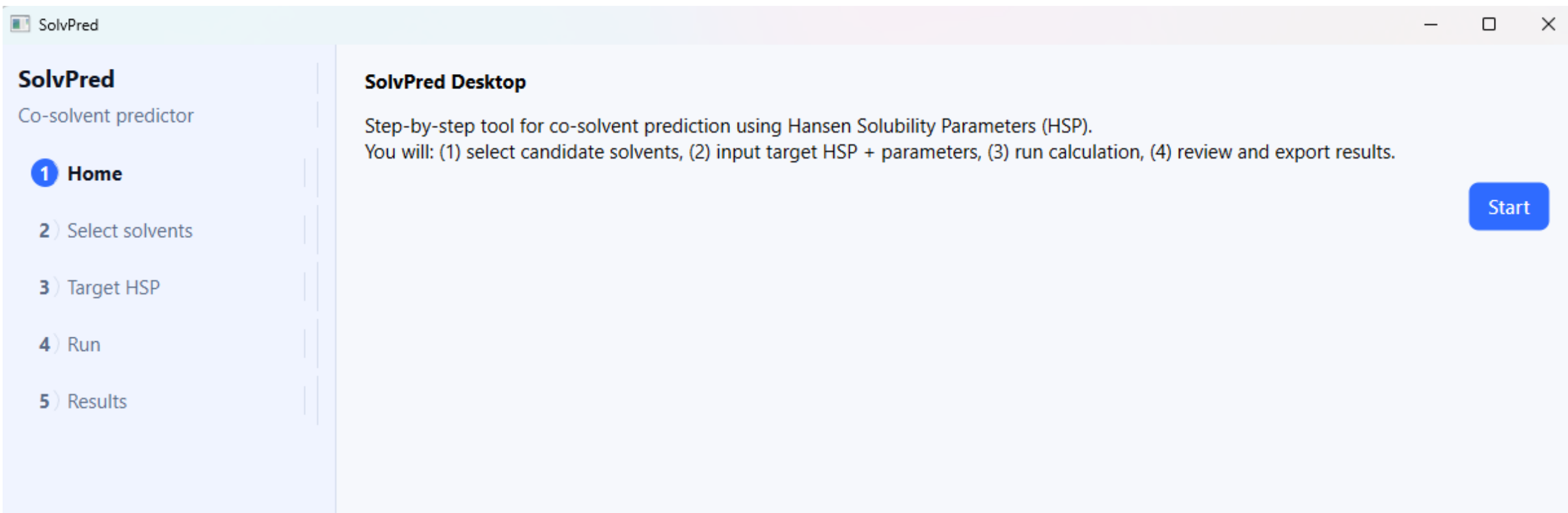

Scheme S 37 Welcome page of the SolvPred GUI.

Users need to click on "Start" to proceed and will be directed to the next solvent candidate selection page (Scheme S 38).

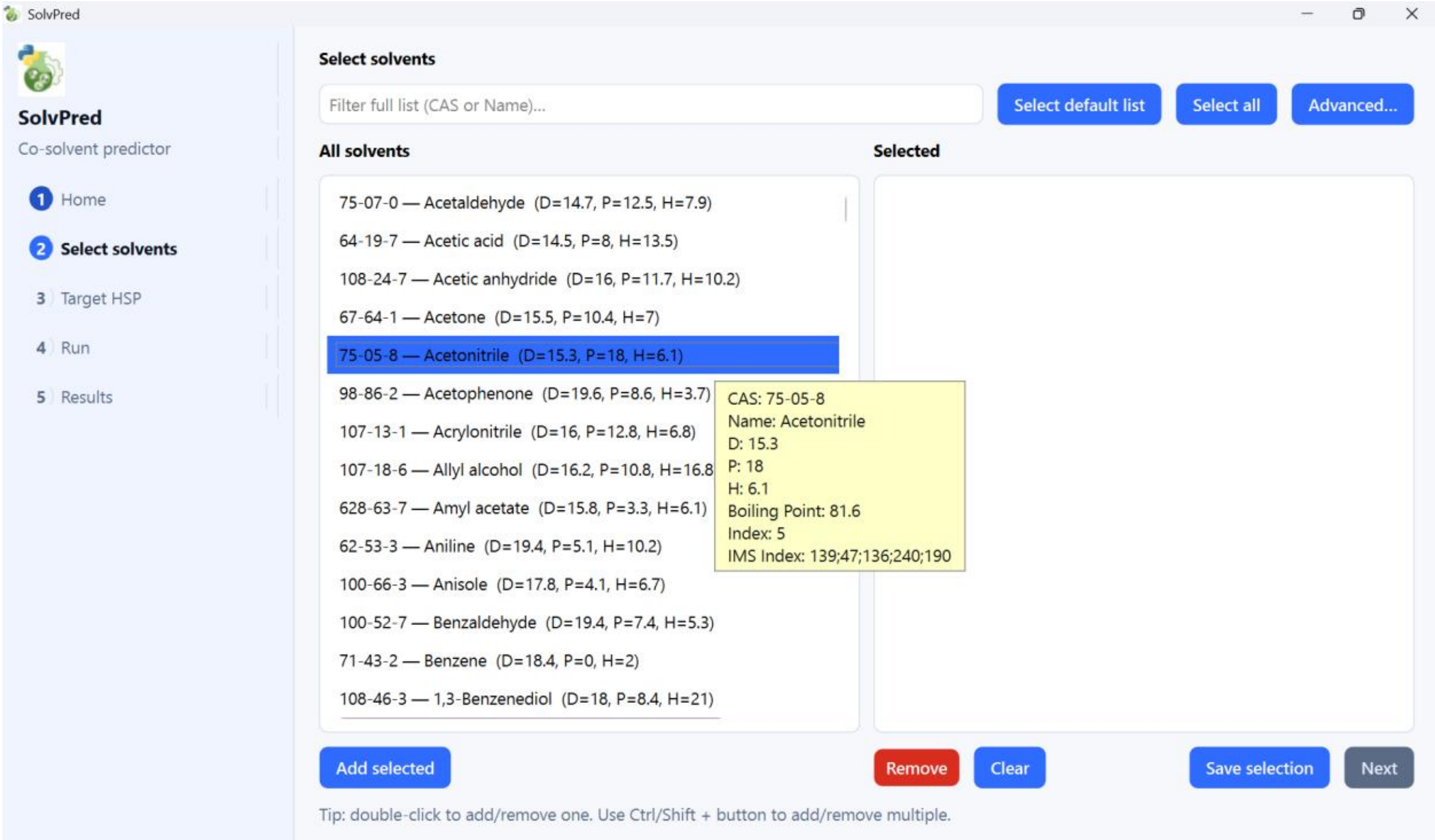

Scheme S 38 A snapshot of the candidate selection page of SolvPred GUI.

The candidate selection page contains two major panels, "All solvents" and "Selected". "All solvents" lists all known solvents to the software as recorded in its default database, while "Selected" demonstrates which of these solvents the user has selected to be candidates for consideration in later calculations.

There are three methods to add solvent candidates.

The first method is to click the "Select default list" button, which will load the default solvent database stored in *"SolvPred_App"* > *"app"* > *"data"* > *"default_solv_candidate.json"* (Scheme S 39).

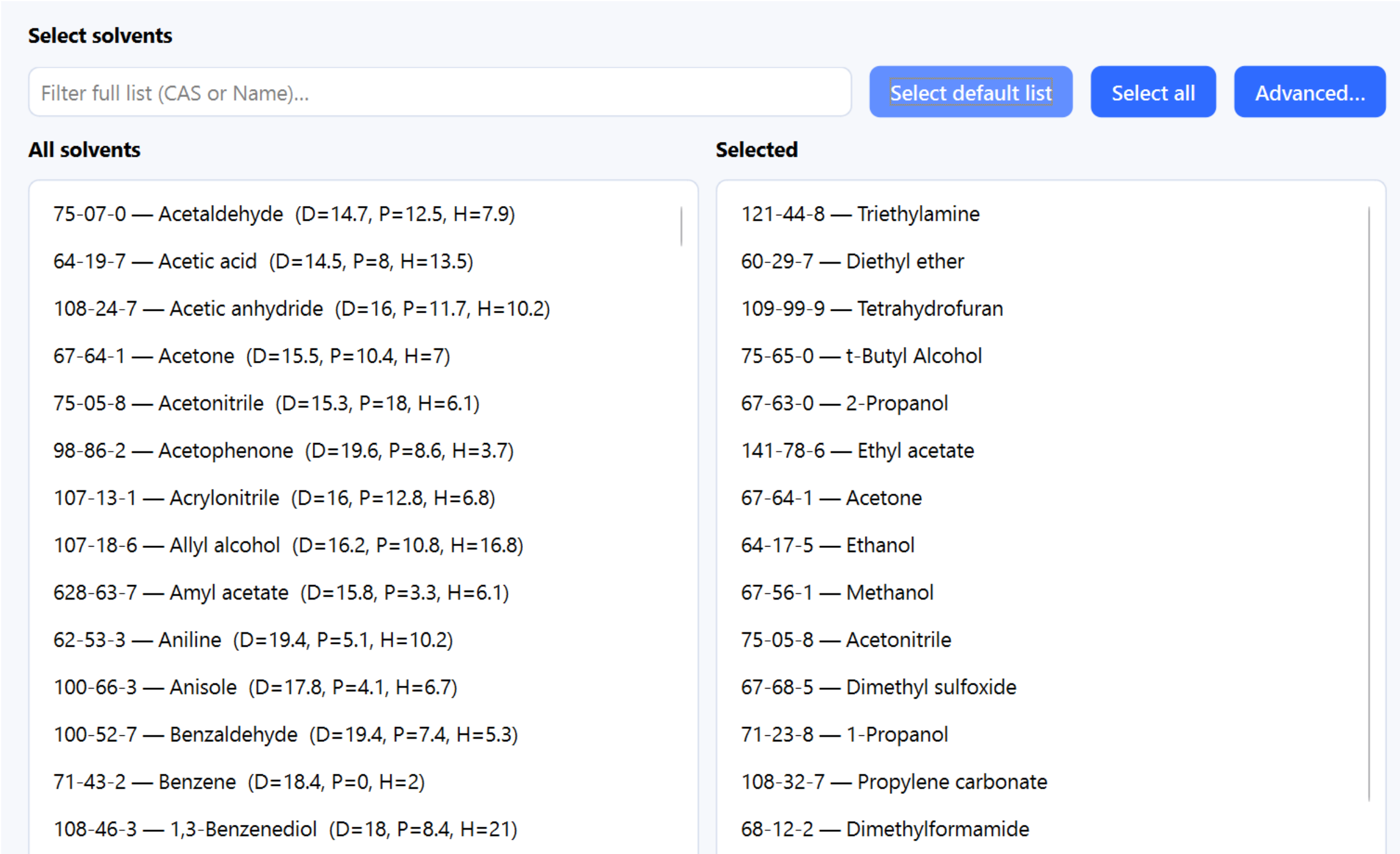

Scheme S 39 Load the default candidate list.

Another way to add a solvent into the selection is to double click on the solvent in the "*All solvents*" list or search CAS No. or to enter part of or the entire CAS number or solvent name in the search box. The *SolvPred* app will filter the entries in the "*All solvents*" panel and display only those that contain the keywords searched (Scheme S 40).

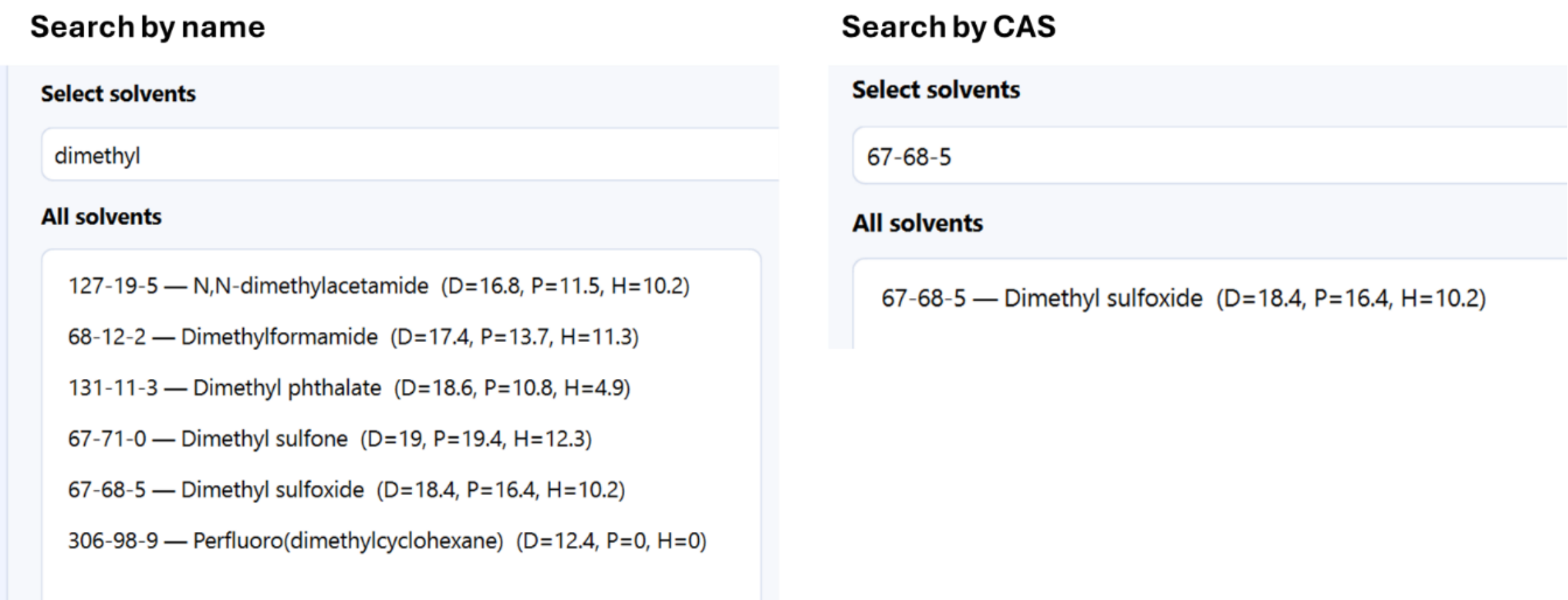

Scheme S 40 An example snapshot of searching solvents by name (left) or CAS (right).

Users can also directly select multiple solvents from the "All solvents" list using keyboard operations *ctrl* or *shift*, and click the "*Add selected*" button to add candidates in a batch (Scheme S 41).

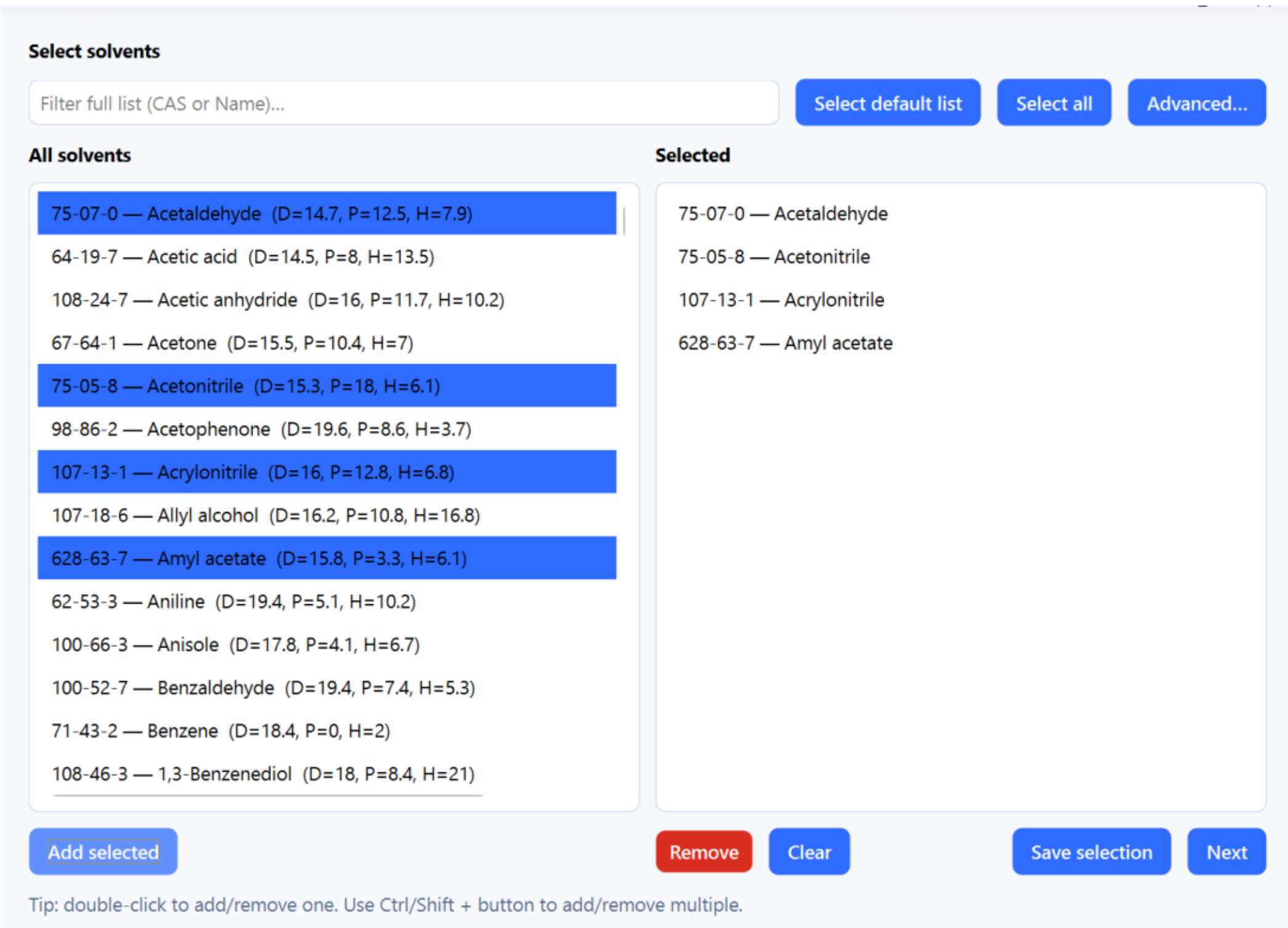

Scheme S 41 An example snapshot of adding multiple candidates in a batch.

Detailed information about the solvent, such as boiling points and immiscible solvent indices, will appear in a floating box when users hover the mouse over the name/line (Scheme S 42).

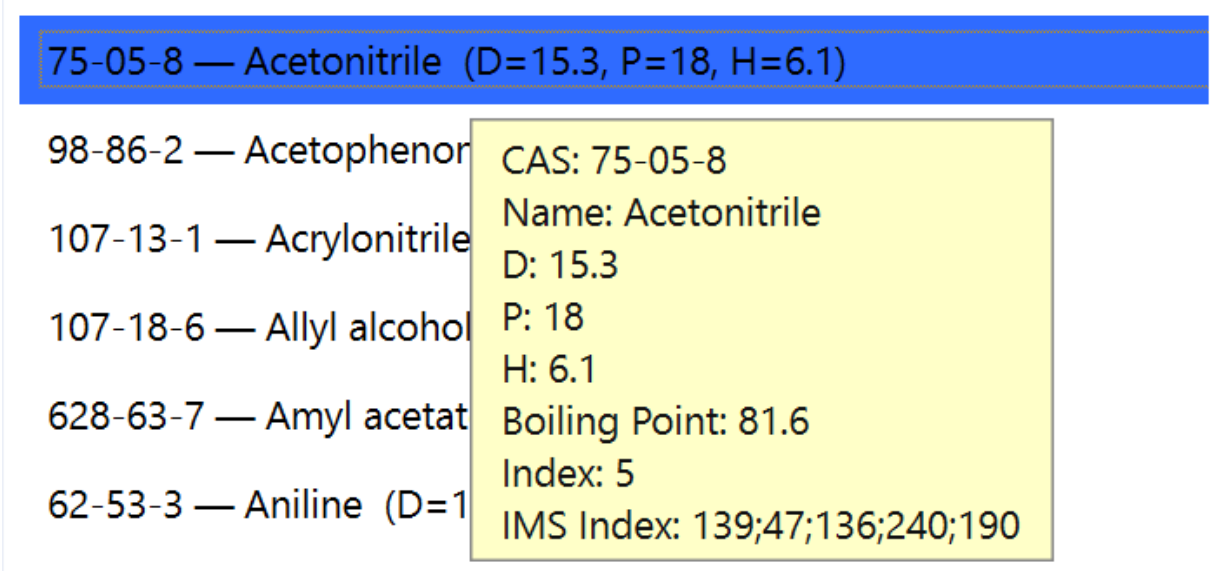

Scheme S 42 An example snapshot that displays full information of each solvent candidate by hovering the cursor upon a solvent.

Solvents can also be removed from the "*Selected*" list using the "*Remove*" button if needed (Scheme S 43).

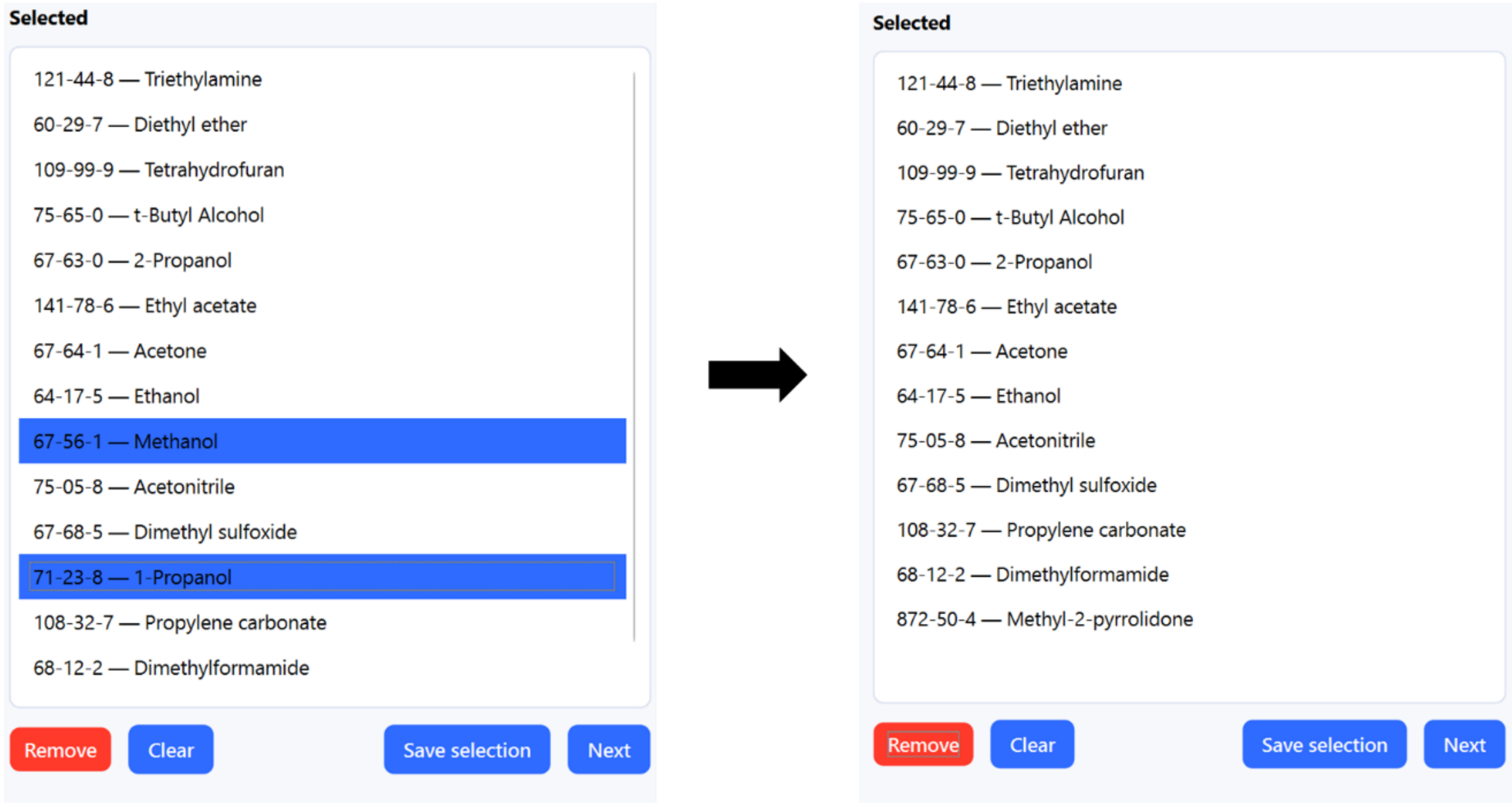

Scheme S 43 An example of removing solvents from the selected list.

The "*Save selection*" button will save the current solvent list in the "*Selected*" panel so users can load the same solvent selection from files in the future (Scheme S 44) *via* the "*Load solvents from file*" in the "*Advanced*" option (Scheme S 45).

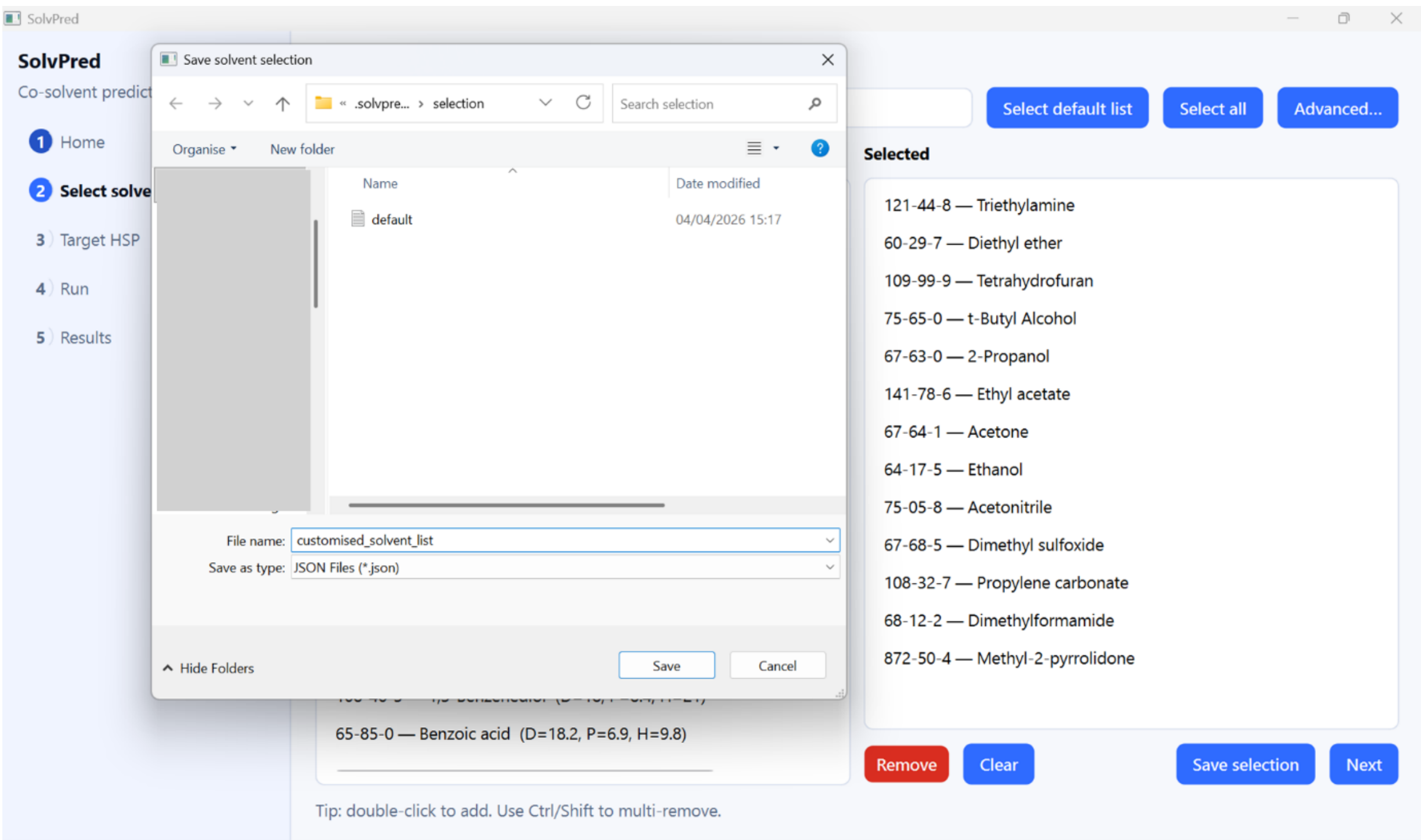

Scheme S 44 An example snapshot to save selected solvents as a "customized_solvent_list" json file for future use.

The *SolvPred* app allows users to set up advanced candidate selection by clicking the "*Advanced*" button. The advanced mode allows users to add custom solvents, load solvents from *json* files, load custom solvent databases or manage existing user database (Scheme S 45).

The "*add custom solvent*" button allows users to add solvents that are not presented in the database. The "*Load solvents from file*" option will load a solvent selection list from an input file. The "*Load user database from file*" option could be useful when users constuct their own solvent database that may contain many of their own custom solvents. The "*Manage user database*" button provides a functionality to allow user add, edit, or remove solvents from the database file as they selected.

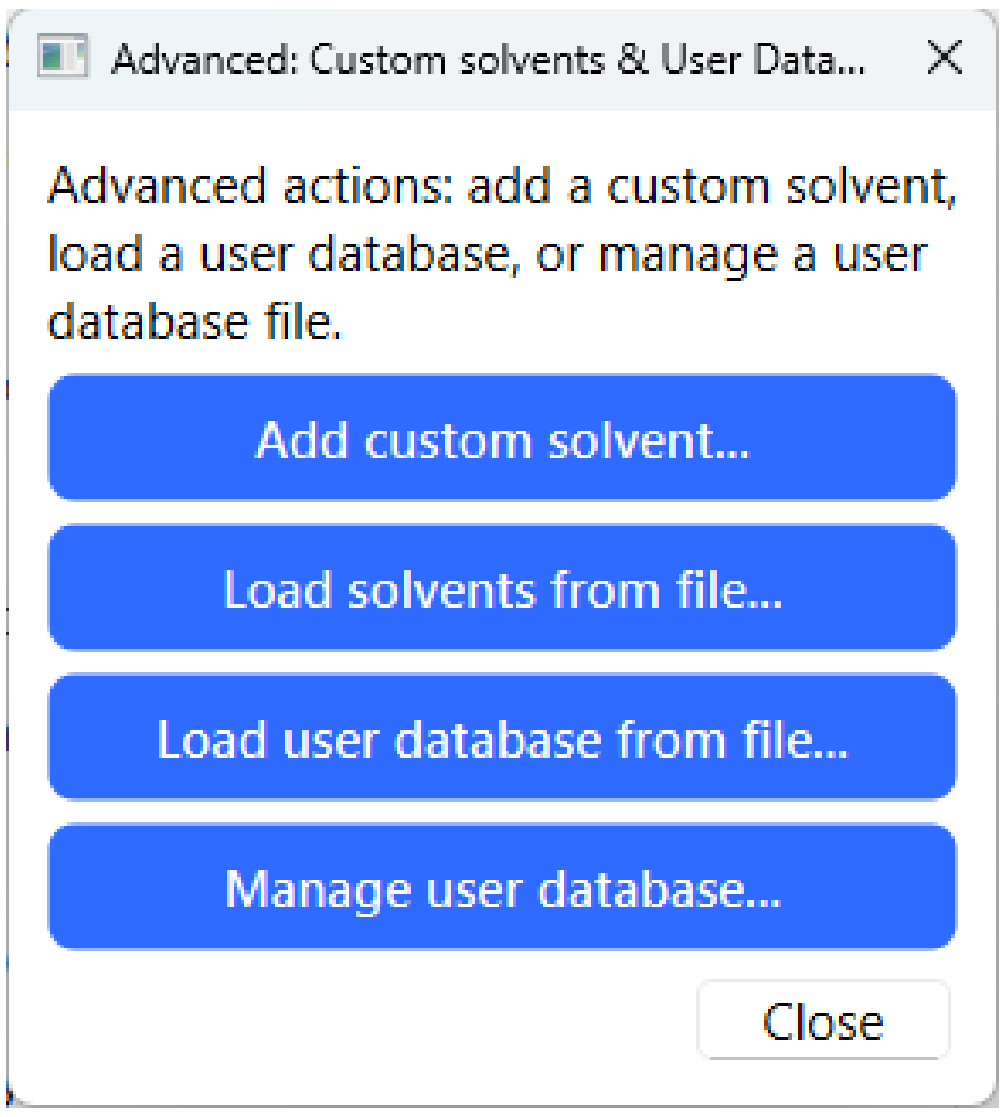

Scheme S 45 A snapshot of the "Advanced" candidate selection option.

Once the solvent selection step is completed, users may enter the target and parameter setup page, the "Target HSP" page, by clicking the "Next" button (Scheme S 46).

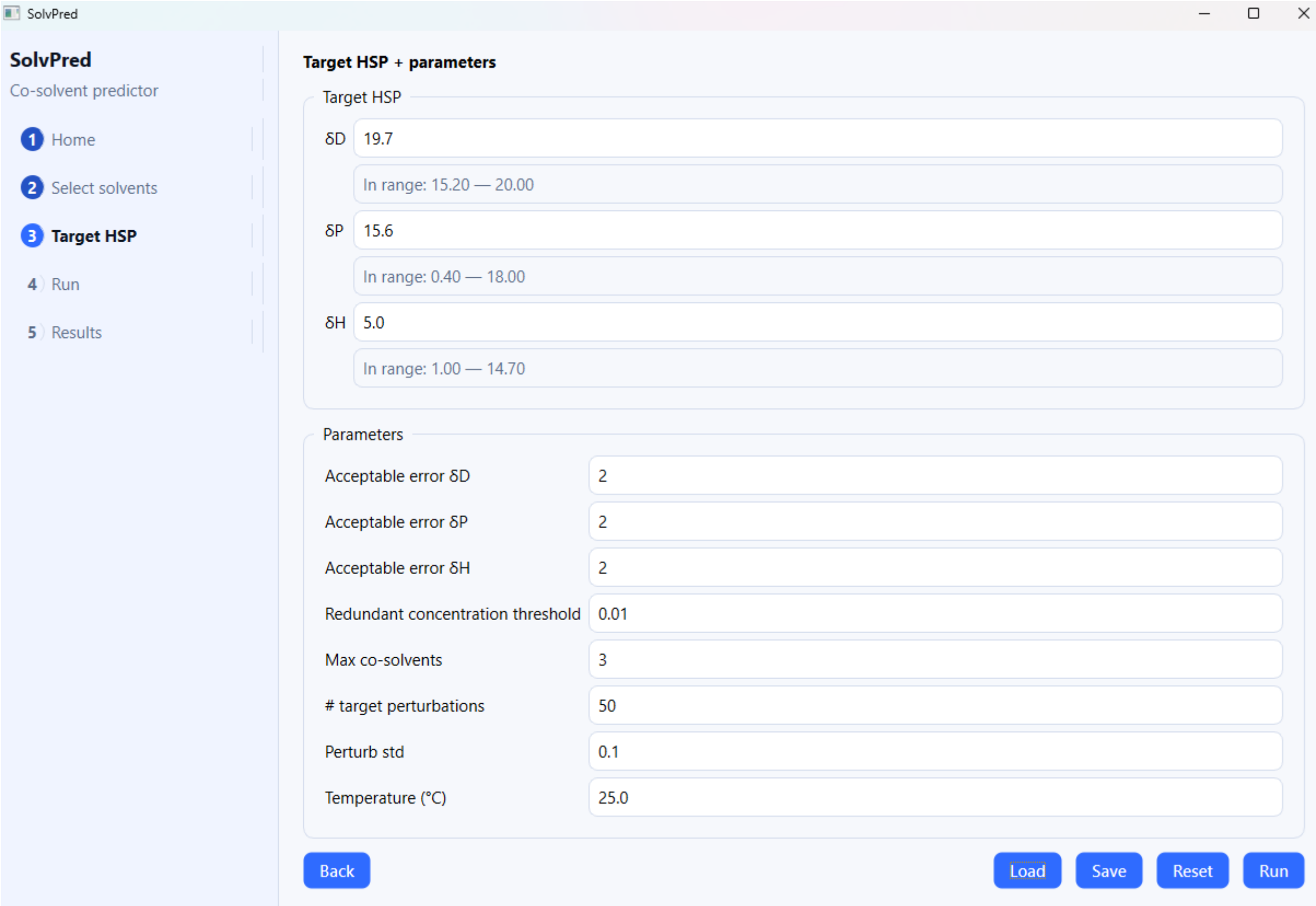

Scheme S 46 The snapshot of the target parameter setting up page.

On the "Target HSP" page, users can adjust target HSP values as well as other hyperparameters required in the *SolvPred* algorithm. For example, the three acceptable error parameters determine how close the calculated HSP has to be to the target HSP to be counted as a valid result. The max co-solvents parameter determines the maximum number of co-solvent included in the solvent mixture. The temperature determines the operating temperature and *SolvPred* will apply a temperature correction according to Eq. S 1 to Eq. S 3.

The "*Save*" and "*Load*" buttons allow users to save the current parameters set into a *json* file or to load these from an additional *json* file (Scheme S 47). The "*Reset*" button will reset the current set of parameters to its default values. The "*Back*" button will lead users back to the last step if users desire to further adjust solvent seletion.

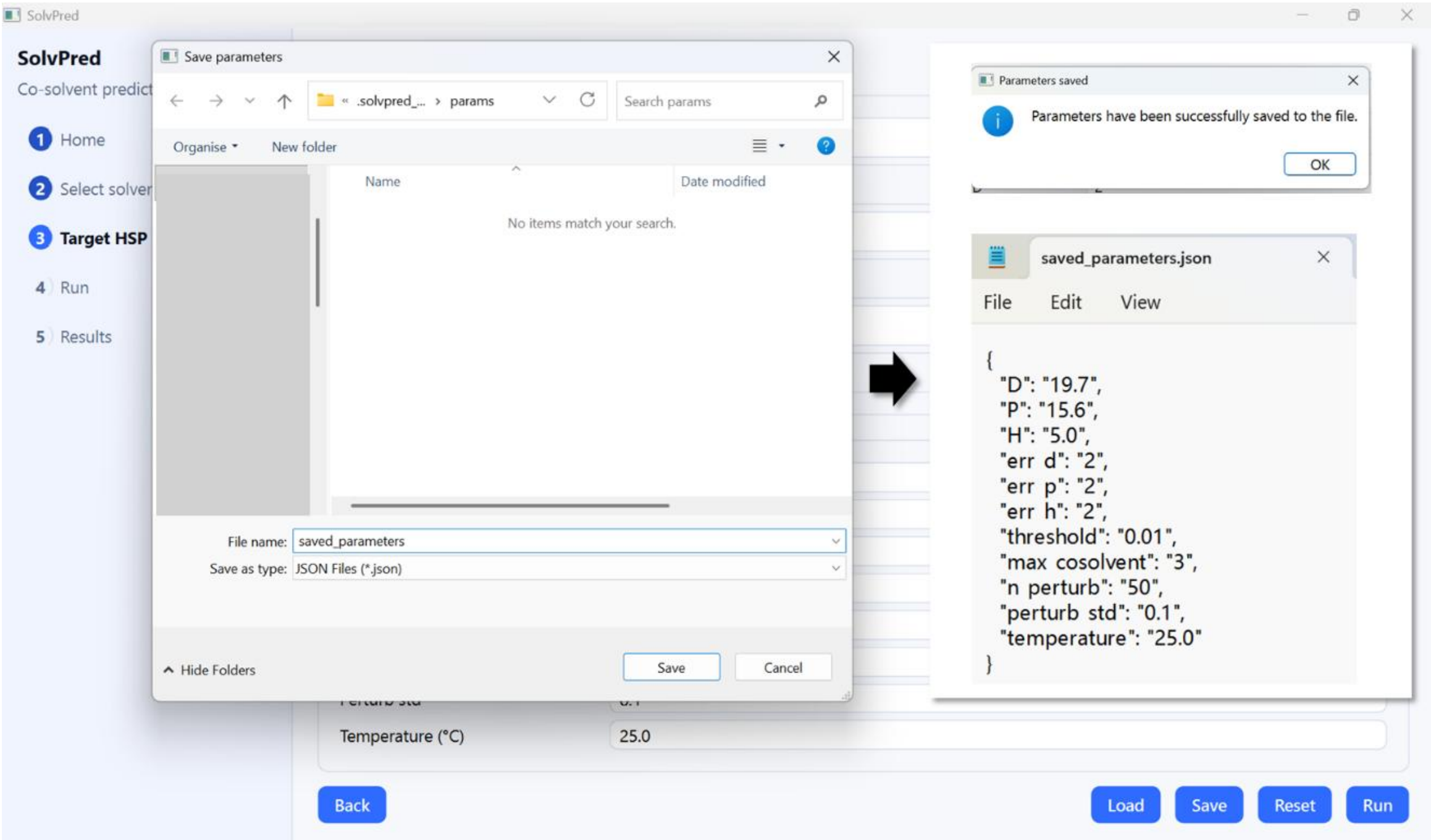

Scheme S 47 A snapshot of saving parameters as a json file.

The *SolvPred* app also provides an HSP target range hint below each target parameter entry. These ranges are automatically calculated based on the solvent selection made in the previous step. Should any of the target parameters falls beyond the given range, it is impossible to find a solution for a solvent mixture with their overall HSPs matching the target (Scheme S 48). This is due to the linear additivity of each candidate HSP based on their concentration and the physical constraint on the concentration, *i.e.* that it should never be larger than 1. As a result, the overall HSP from solvent mixtures should only appear within the realm of a polyhedron constructed by each candicate solvent as vertices.

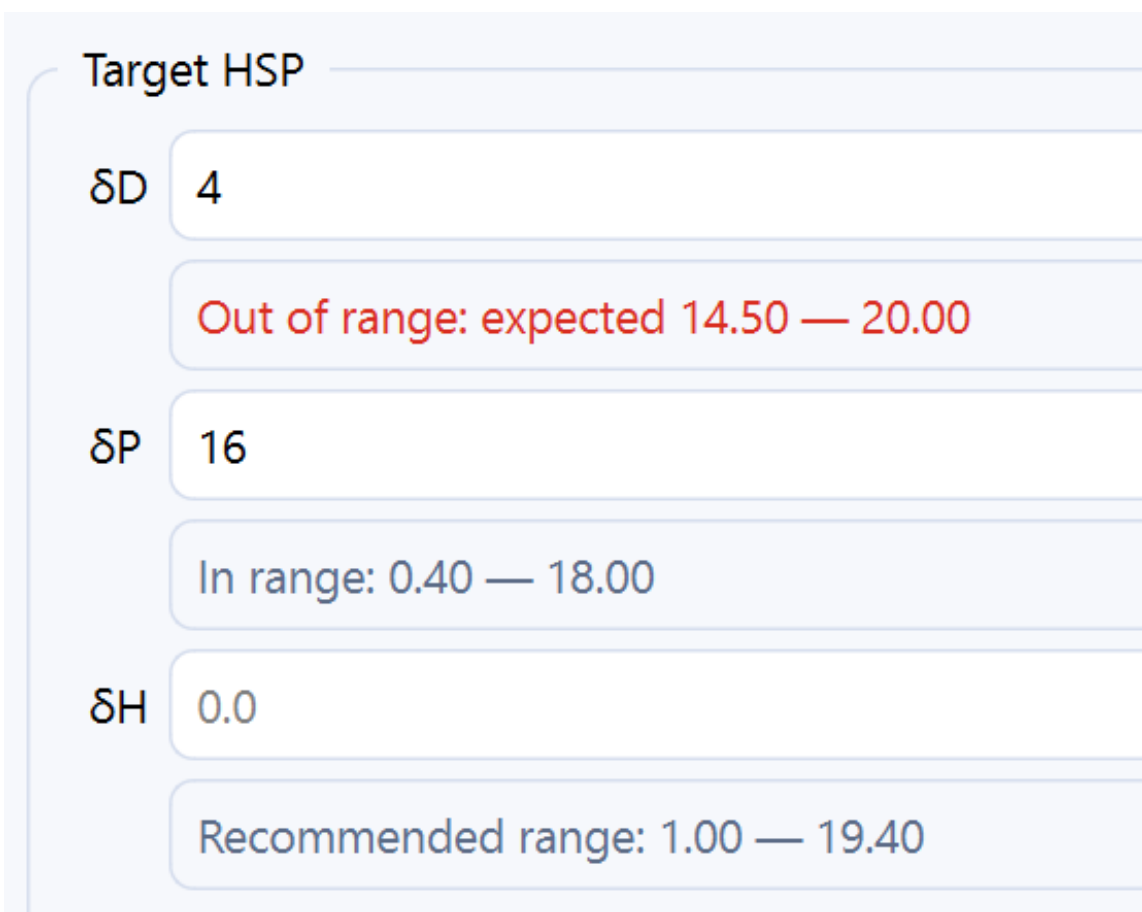

Scheme S 48 A snapshot of example achievable target HSP hints for each HSP dimension. Recommended range will display by default according to the selected solvents. A warning message will display if the input value exceeds the achievable range of target HSPs.

After all parameters are set, users can proceed to the run page by clicking the "*Run*" button.

The next page is the "*Run calculation*" page, where users can select filters prior to the main calculation. The filter functions provided at this stage examine the solvent selection based on the parameters from the target HSP page and the filter functionalities. The "*Check Boiling Point*" filter will only keep solvents whose boiling point is below the temperature from the target HSP setting, and the "*Check Miscibility*" filter will keep results for cases where all co-solvents are expected to be miscible. The text at the bottom of the filter window suggests the total number of solvents being selected and the number of remaining solvents after this step (Scheme S 49).

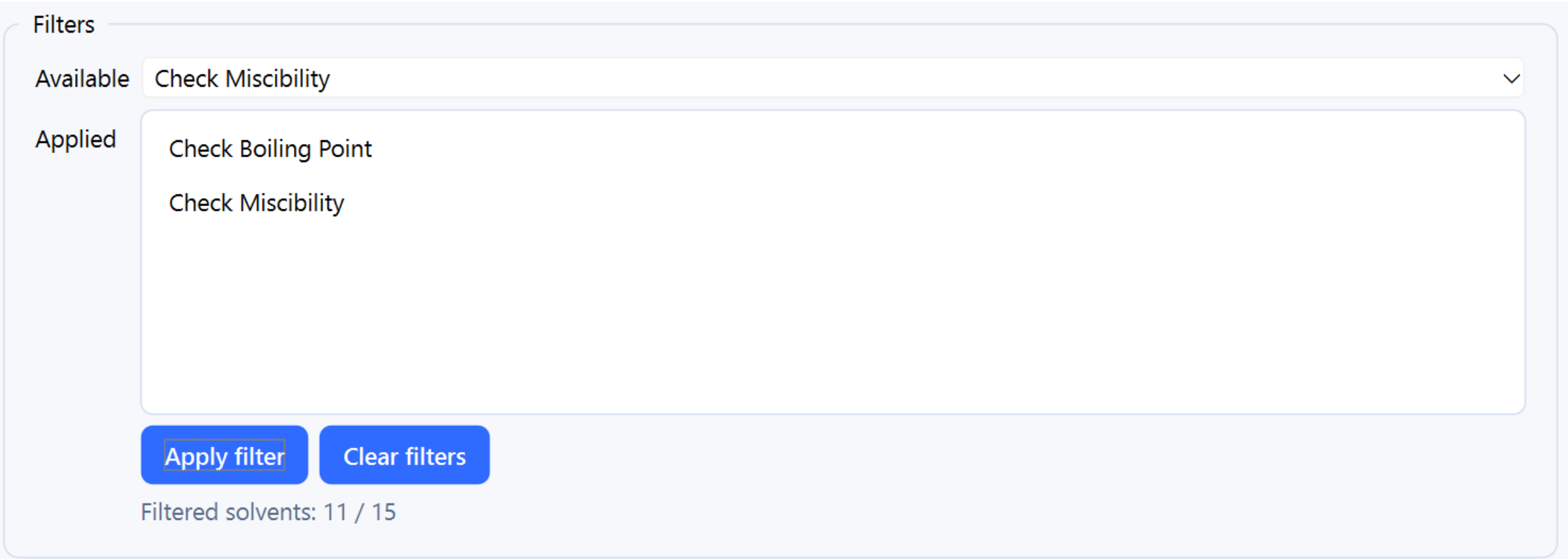

Scheme S 49 The "Run calculation" page and steps to apply filters to check boiling points and miscibility.

Once the filters have been applied, users can click the "*Run*" button. *SolvPred* will then run the calculations and summarize results on the "*Results*" page (Scheme S 50). The solvent mixture results are presented as a table. At the bottom of the table, users can filter results based on solvents and the number of solvents.

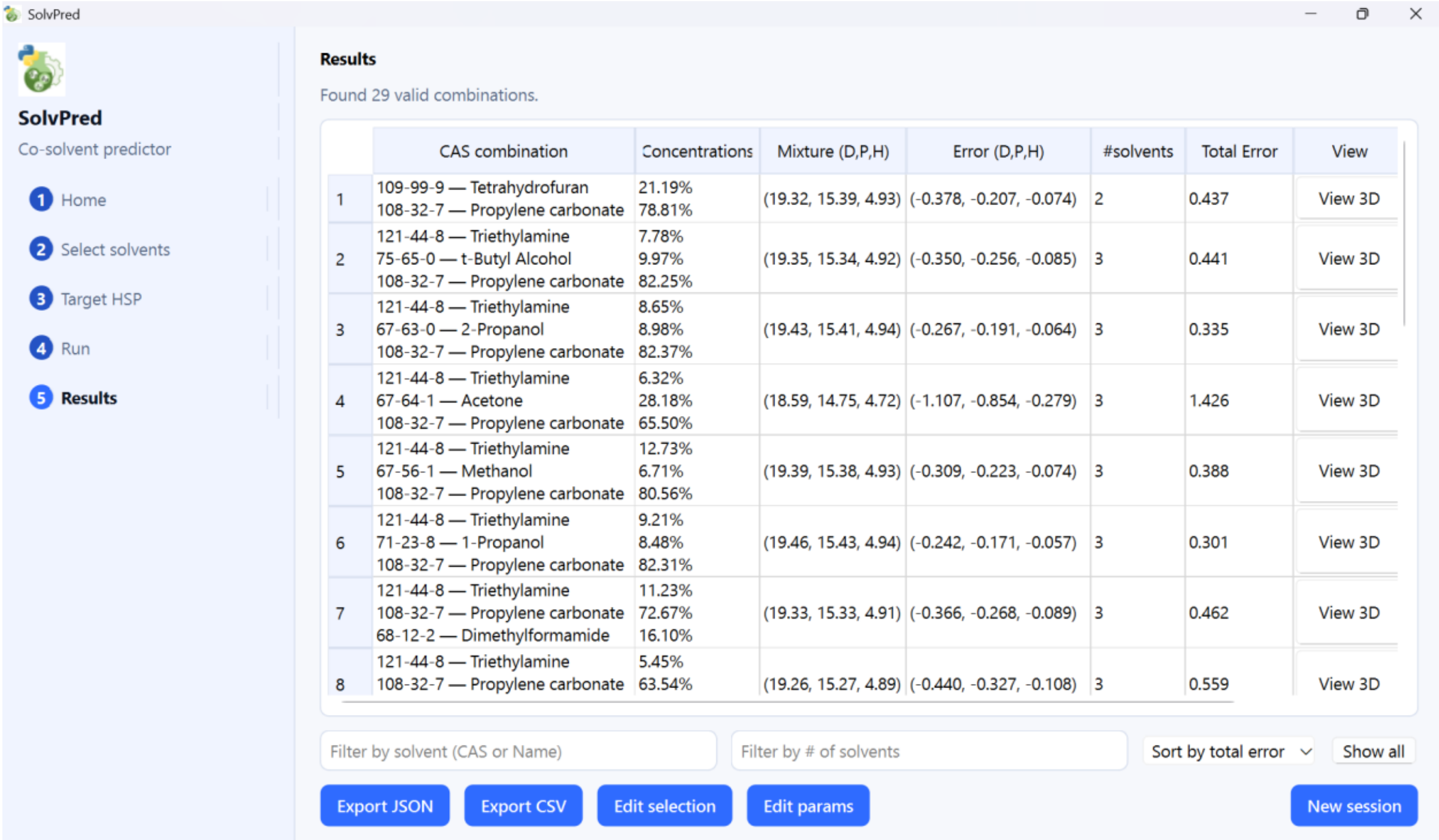

Scheme S 50 The snapshot of the "Results" page.

For example, if users only want solvent combinations that contains triethylamine (TEA), users can type either the full name or the corresponding CAS number of TEA in the "filter by solvent" text box. The filter also allows users to specify the number of co-solvents in the mixture after calculation. This can be fewer than the initial maximum number of co-solvents users specified at the beginning, due to the removal of potentially redundant solvents. Users can also sort the results table by either total HSP error or partial error of a particular HSP component (Scheme S 51).

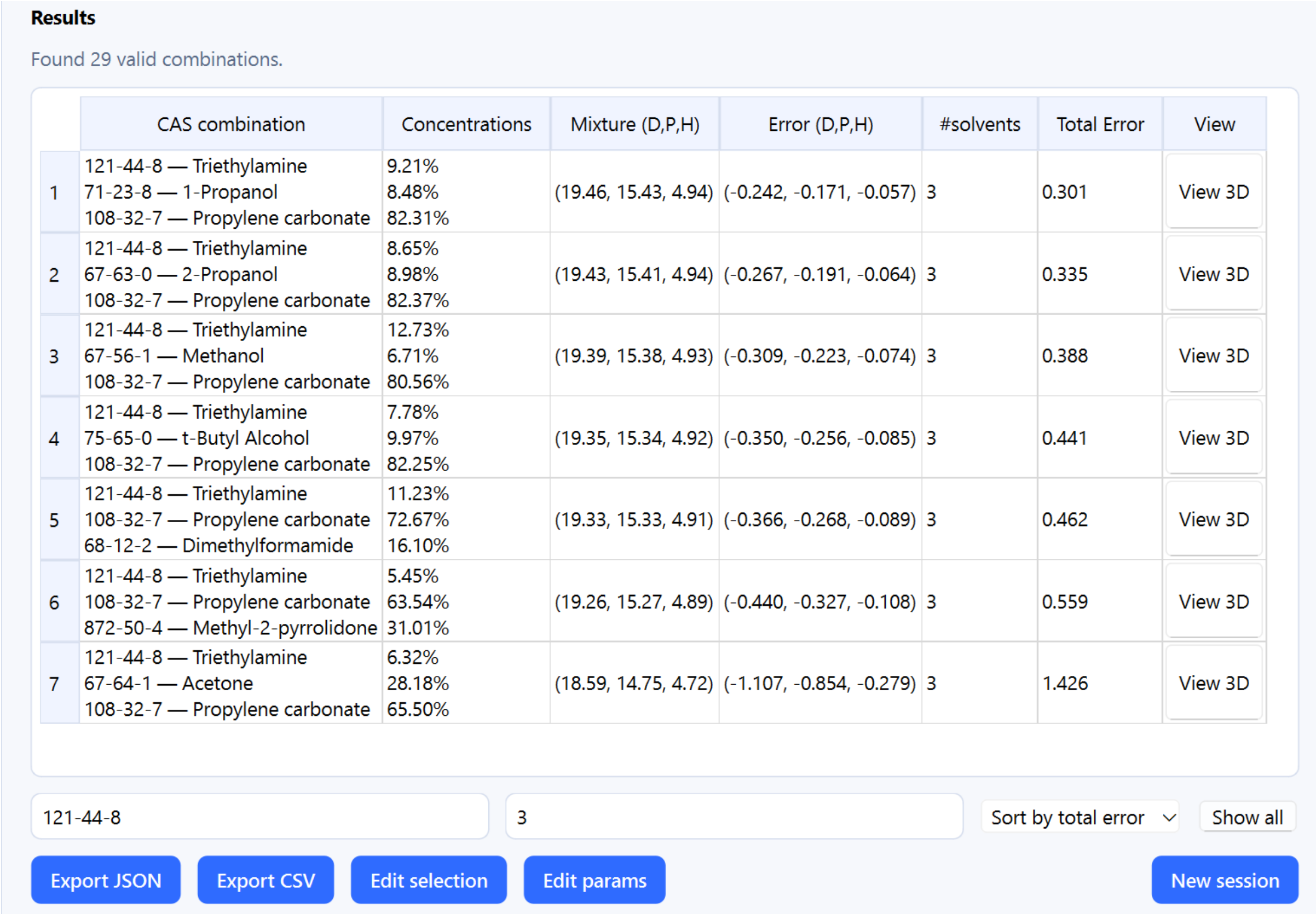

	CAS combination	Concentrations	Mixture (D,P,H)	Error (D,P,H)	#solvents	Total Error	View
1	121-44-8 — Triethylamine<br>71-23-8 — 1-Propanol<br>108-32-7 — Propylene carbonate	9.21%<br>8.48%<br>82.31%	(19.46, 15.43, 4.94)	(-0.242, -0.171, -0.057)	3	0.301	View 3D
2	121-44-8 — Triethylamine<br>67-63-0 — 2-Propanol<br>108-32-7 — Propylene carbonate	8.65%<br>8.98%<br>82.37%	(19.43, 15.41, 4.94)	(-0.267, -0.191, -0.064)	3	0.335	View 3D
3	121-44-8 — Triethylamine<br>67-56-1 — Methanol<br>108-32-7 — Propylene carbonate	12.73%<br>6.71%<br>80.56%	(19.39, 15.38, 4.93)	(-0.309, -0.223, -0.074)	3	0.388	View 3D
4	121-44-8 — Triethylamine<br>75-65-0 — t-Butyl Alcohol<br>108-32-7 — Propylene carbonate	7.78%<br>9.97%<br>82.25%	(19.35, 15.34, 4.92)	(-0.350, -0.256, -0.085)	3	0.441	View 3D
5	121-44-8 — Triethylamine<br>108-32-7 — Propylene carbonate<br>68-12-2 — Dimethylformamide	11.23%<br>72.67%<br>16.10%	(19.33, 15.33, 4.91)	(-0.366, -0.268, -0.089)	3	0.462	View 3D
6	121-44-8 — Triethylamine<br>108-32-7 — Propylene carbonate<br>872-50-4 — Methyl-2-pyrrolidone	5.45%<br>63.54%<br>31.01%	(19.26, 15.27, 4.89)	(-0.440, -0.327, -0.108)	3	0.559	View 3D
7	121-44-8 — Triethylamine<br>67-64-1 — Acetone<br>108-32-7 — Propylene carbonate	6.32%<br>28.18%<br>65.50%	(18.59, 14.75, 4.72)	(-1.107, -0.854, -0.279)	3	1.426	View 3D

Scheme S 51 An example of filtering results by CAS No., number of solvents (3) and sort the results by total error.

Hovering the cursor on each entry will again display the full property information and HSPs of each solvent, and clicking the "*View 3D*" button on each entry will display an interactive 3D plot of Hansen space (Scheme S 52). Users can save the 3D plot by clicking the "*Save PNG*" button.

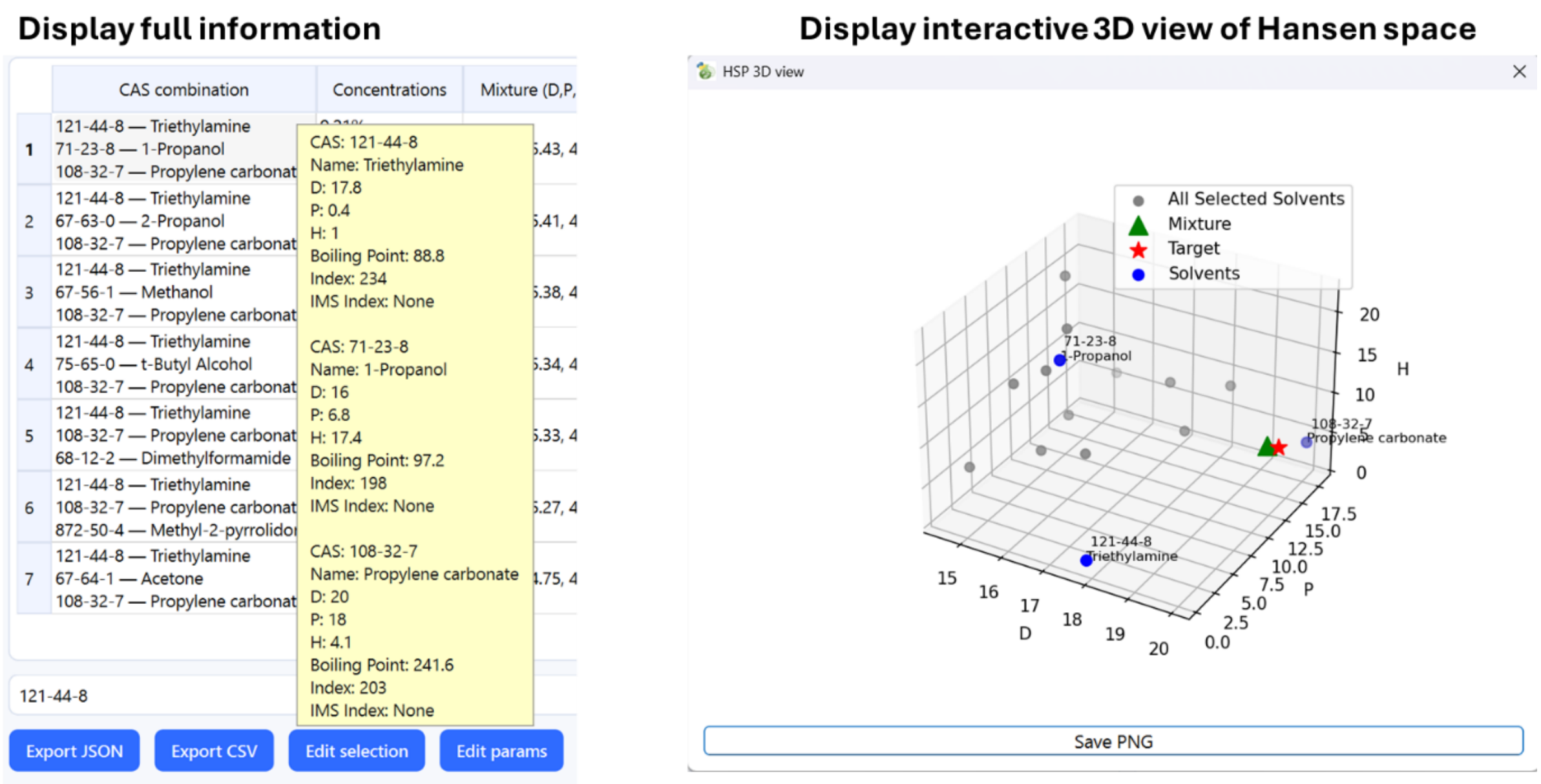

Scheme S 52 Display full information of each co-solvent within each group by hovering the cursor on the corresponding entry (left). Display an interactive 3D plot of Hansen space by clicking the "3D View" option for each entry.

Users can save the results table and full metadata into *json* or *csv* files by clicking the "*Export JSON*" and "*Export CSV*" buttons, respectively. The results (Scheme S 53) are typically stored under "*SolvPred_App*" > "*dist*" directory and can be modified by users as needed.

group	cas	name	concentration_pct	mix_D	mix_P	mix_H	err_D	err_P	err_H	n_solvents	total_error
1	121-44-8	Triethylamine	9.21	19.4581	15.4294	4.9428	-0.2419	-0.1706	-0.0572	3	0.3015
1	71-23-8	1-Propanol	8.48	19.4581	15.4294	4.9428	-0.2419	-0.1706	-0.0572	3	0.3015
1	108-32-7	Propylene carbonate	82.31	19.4581	15.4294	4.9428	-0.2419	-0.1706	-0.0572	3	0.3015
2	121-44-8	Triethylamine	8.65	19.4326	15.4092	4.9363	-0.2674	-0.1908	-0.0637	3	0.3346
2	67-63-0	2-Propanol	8.98	19.4326	15.4092	4.9363	-0.2674	-0.1908	-0.0637	3	0.3346
2	108-32-7	Propylene carbonate	82.37	19.4326	15.4092	4.9363	-0.2674	-0.1908	-0.0637	3	0.3346
3	121-44-8	Triethylamine	12.73	19.3912	15.3765	4.9258	-0.3088	-0.2235	-0.0742	3	0.3883
3	67-56-1	Methanol	6.71	19.3912	15.3765	4.9258	-0.3088	-0.2235	-0.0742	3	0.3883
3	108-32-7	Propylene carbonate	80.56	19.3912	15.3765	4.9258	-0.3088	-0.2235	-0.0742	3	0.3883
4	121-44-8	Triethylamine	7.78	19.3503	15.3442	4.9153	-0.3497	-0.2558	-0.0847	3	0.4415
4	75-65-0	t-Butyl Alcohol	9.97	19.3503	15.3442	4.9153	-0.3497	-0.2558	-0.0847	3	0.4415
4	108-32-7	Propylene carbonate	82.25	19.3503	15.3442	4.9153	-0.3497	-0.2558	-0.0847	3	0.4415
5	121-44-8	Triethylamine	11.23	19.3343	15.3316	4.9113	-0.3657	-0.2684	-0.0887	3	0.4622
5	108-32-7	Propylene carbonate	72.67	19.3343	15.3316	4.9113	-0.3657	-0.2684	-0.0887	3	0.4622
5	68-12-2	Dimethylformamide	16.1	19.3343	15.3316	4.9113	-0.3657	-0.2684	-0.0887	3	0.4622
6	121-44-8	Triethylamine	5.45	19.2598	15.2727	4.8923	-0.4402	-0.3273	-0.1077	3	0.559
6	108-32-7	Propylene carbonate	63.54	19.2598	15.2727	4.8923	-0.4402	-0.3273	-0.1077	3	0.559
6	872-50-4	Methyl-2-pyrrolidone	31.01	19.2598	15.2727	4.8923	-0.4402	-0.3273	-0.1077	3	0.559
7	121-44-8	Triethylamine	6.32	18.5929	14.7459	4.7212	-1.1071	-0.8541	-0.2788	3	1.4257
7	67-64-1	Acetone	28.18	18.5929	14.7459	4.7212	-1.1071	-0.8541	-0.2788	3	1.4257
7	108-32-7	Propylene carbonate	65.5	18.5929	14.7459	4.7212	-1.1071	-0.8541	-0.2788	3	1.4257

Scheme S 53 A snapshot of metadata saved via "Export csv" option.

The "*Edit selection*" or the "*Edit params*" button will lead users back to the solvent selection page or the target HSP page, respectively, if users wish to make some adjustments to the current results. Alternatively, users can restart the whole process by clicking the "New session" button.

S1.4 Computing performance and sensitivity tests

To evaluate the computing performance of the *SolvPred* algorithm, the memory usage and runtime were recorded for calculations of binary (n=2), ternary (n=3), quaternary (n=4) and n=5 cases for different numbers of solvent candidates. Full solvent database was applied as candidates for the n=2 and n=3 cases to provide a baseline runtime estimation.

Computing performance test was done with memory profiling tools `memory_profiler` (v 0.61.0) and `psrecord` (v 1.4). The memory and runtime were recorded by running the following commands in `Anaconda prompt`:

```
pip install memory_profiler psrecord

mprof run python solv_pred_main.py

mprof plot --output solvpred_memory.png
```

`Matplotlib` (v 3.5.3) is required to be installed to run the previous command.

To get the peak memory, the following command was used:

```
mprof peak
```

Runtime was used as the main indicator to describe the computing performance, which was done on a Windows 11 personal laptop (16 GB RAM, 128 MB Graphic card, 12th Gen Intel(R) Core(TM) i7-1260P processor).

In *SolvPred*, computing performance is determined by the size of the candidate pool, the number of co-solvents, the target – how well it is centered by the candidates, which subsequently affects the size of successfully predicted results and the computing cost for the advanced filtering step.

The computing can be broadly classified into three stages: 1) the initialization step, with *SolvPred* importing required packages, loading databases and setting up calculation parameters; 2) the main calculation step, when *SolvPred* calculates possible candidate combinations, followed by conducting the pseudoinverse calculation of the candidate matrix and redundant solvent filtering, renormalization and error filtering, along with writing results; 3) the advanced filtering step and final results writing.

Stage 2 is expected to be the most intensive stage, with runtime growing according to a binomial coefficient associated with the total number of solvent candidates (N) and the target number of solvents in the mixture (n),

$$C(N,n) = \frac{N!}{n!(N-n)!}. \qquad Eq.\ S\ 27$$

For each combination, solving the pseudoinverse of the $\boldsymbol{\delta_{cand}}$ matrix using the singular value decomposition method implemented in `scipy.linalg` can lead to another $O(n^3)$ complexity. The redundant solvent filtering, renormalization and error filtering steps can each lead to an $O(n)$ complexity, depending on how many iterations are executed that may vary from case to case. When n is significantly smaller than N, the major computing cost arises from $N!$.

Computing cost of Stage 3 depends on the number of successfully predicted results. For each predicted group, boiling point checking follows an $O(n)$ complexity, *i.e.*, by looping over all n solvents and querying their boiling points. The miscibility checking leads to an $O(n^2)$ complexity, by first looping over all n solvents in a predicted mixture group, followed by querying the immiscible solvent index to compare with the indices of the rest of solvents in the same group. The total number of predicted groups is further affected by how well the target is surrounded by candidates: a target closer to the center of the convex hull formed by the candidates is more likely to yield more successful results.

It is also worth noting that, when n exceeds 3, the solvent candidate matrix, $\boldsymbol{\delta_{cand}}$, with the dimension of 3 x n, becomes increasingly flat. The difference between n and 3 leads to null space dimension - if solvent candidates with comparable HSPs exist, they will introduce collinearity, making the pseudoinverse of $\boldsymbol{\delta_{cand}}$ more challenging to solve.

Therefore, the candidates for high-level solvent mixtures need to scatter as diverse as possible in the Hansen space.

Guided runtime is provided for different scenarios here, including using full 249-solvent database as candidates for 2 and 3 co-solvent mixtures, and using two smaller subsets (15 and 35 candidates) for 2-5 co-solvent mixtures with target HSPs well- or poorly surrounded by the candidates. Finer resolutions and error thresholds and redundant solvent thresholds were also studied, but they demonstrated limited impact comparably.

Computing performance using all 249 solvents as candidates was first tested. The error of each HSP dimension was set as 2.0 MPa$^{1/2}$; the redundant solvent threshold was set as 1%; the target HSPs were set as δ_D = 19.7 MPa$^{1/2}$, δ_P = 15.6 MPa$^{1/2}$, and δ_H = 11.2 MPa$^{1/2}$, respectively – same as the HSPs of NMI. The results are summarized in Table S 7 and illustrated in Figure S 2. Computing for binary solvents finished in 16 s. The total runtime grew to 90 min for ternary solvents. Stage 2 appeared as the dominant step as expected, lasting for 12 s for binary solvents and 48 min for ternary solvents. Stage 1 remained stable for 0.44 s for both systems.

Table S 7 Details of memory usage and runtime of the calculation steps of SolvPred for n=2 and 3 using 249 full solvent list. All errors = 2.0 MPa$^{1/2}$; redundant solvent threshold = 1%; target HSPs: δ_D = 19.7 MPa$^{1/2}$, δ_P = 15.6 MPa$^{1/2}$, and δ_H = 11.2 MPa$^{1/2}$.

<table>
<tr><th>Stage</th><th>Description</th><th>Number of solvents (n)</th><th>Peak memory/MB</th><th>Runtime/s[a]</th></tr>
<tr><td rowspan="2">1</td><td rowspan="2">Initialization; users setting calculation parameters and defining candidates</td><td>2</td><td>36.34</td><td>0.44</td></tr>
<tr><td>3</td><td>31.24</td><td>0.44</td></tr>
<tr><td rowspan="2">2</td><td rowspan="2">SolvPred calculating solvent combinations, conducting matrix pseudoinverse calculations, redundant solvent filtering, renormalization and error filtering, and writing results</td><td>2</td><td>202.83</td><td>12.22</td></tr>
<tr><td>3</td><td>9,618.32</td><td>2,877.25</td></tr>
<tr><td rowspan="2">3</td><td rowspan="2">SolvPred carrying out advanced filtering and writing results</td><td>2</td><td>169.84</td><td>3.63</td></tr>
<tr><td>3</td><td>5,836.49</td><td>2,464.46</td></tr>
</table>

[a] Total runtime would also be affected by the setting up time varied by users

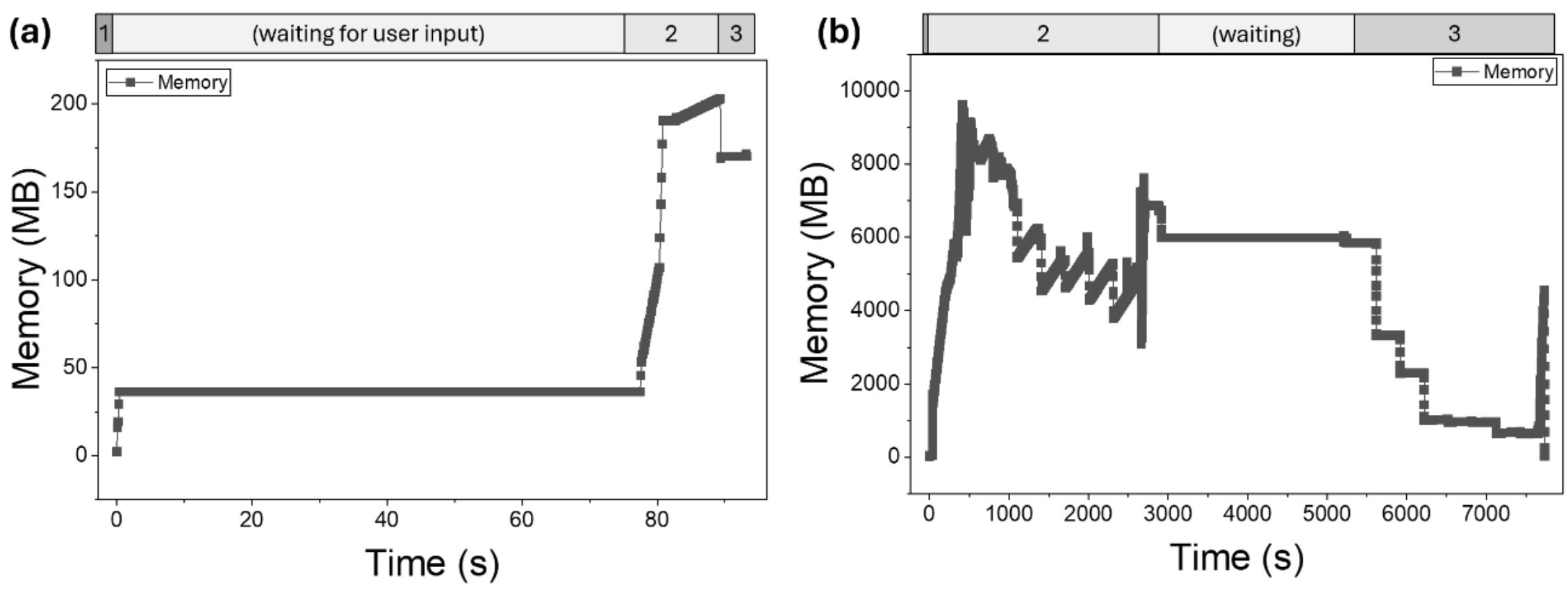

Figure S 2 Memory usage and runtime profiles of the calculation steps of SolvPred for (a) n=2 and (b) n=3 using 249 full solvent list. All errors = 2.0 MPa$^{1/2}$; redundant solvent threshold = 1%; target HSPs: δ_D = 19.7 MPa$^{1/2}$, δ_P = 15.6 MPa$^{1/2}$, and δ_H = 11.2 MPa$^{1/2}$.

Reducing the size of candidates can cut down the runtime significantly. Two representative candidate lists provided by *SolvPred* in the `candidate_pool` folder, covering 15 (Table S 8) and 35 (Table S 9) common lab solvents that distributed well in the Hansen space, were tested.

Table S 8 Candidate list of 15 solvents provided by SolvPred

CAS	Solvent
121-44-8	Triethylamine
60-29-7	Diethyl ether
109-99-9	Tetrahydrofuran
75-65-0	t-butyl alcohol
67-63-0	2-propanol
141-78-6	ethyl acetate
123-91-1	dioxane
67-64-1	acetone
110-86-1	pyridine
64-17-5	ethyl alcohol
67-56-1	methanol
75-05-8	acetonitrile
67-68-5	dimethylsulfoxide
872-50-4	N-Methyl-2-pyrrolidone(NMP)
127-19-5	N,N-dimethylacetamide

Table S 9 Candidate list of 35 solvents provided by SolvPred

CAS	Solvent
121-44-8	Triethylamine
109-66-0	Pentane
1330-20-7	Xylene
60-29-7	Diethylether
109-99-9	Tetrahydrofuran

110-54-3	Hexane
79-01-6	Trichloroethylene
142-82-5	Heptane
110-82-7	Cyclohexane
108-88-3	toluene
71-43-2	benzene
108-90-7	chlorobenzene
75-09-2	methylene dichloride
75-65-0	t-butyl alcohol
67-63-0	2-propanol
141-78-6	ethyl acetate
123-91-1	dioxane
71-23-8	1-propanol
67-64-1	acetone
110-86-1	pyridine
64-17-5	ethyl alcohol
68-12-2	dimethylformamide
67-56-1	methanol
108-95-2	phenol
75-05-8	acetonitrile
67-68-5	dimethylsulfoxide
74-87-3	Methylcyclohexane
67-66-3	chloroform
108-32-7	Propylene carbonate
91-22-5	Quinoline
108-39-4	m-Cresol
108-67-8	Mesitylene
872-50-4	N-Methyl-2-pyrrolidone
56-81-5	Glycerol
127-19-5	N,N-dimethylacetamide

With same setup, using 15 solvents as candidates can yield ternary mixtures successfully within 1 s, comparable to the case of binary mixtures (Table S 10). Using 35 candidates yield ternary mixtures within 12 s, and quaternary mixtures within 25 s.

Table S 10 Details of memory usage and runtime of the calculation steps of SolvPred for n=2 to 4 using 15 and 35 candidates. All errors = 2.0 MPa$^{1/2}$; redundant solvent threshold = 1%; target HSPs: δ_D = 19.7 MPa$^{1/2}$, δ_P = 15.6 MPa$^{1/2}$, and δ_H = 11.2 MPa$^{1/2}$.

Number of candidates (N)	Stage	Number of solvents (n)	Peak memory/MB	Runtime/s
15	2	2	37.53	0.34
		3	39.39	0.22
	3	2	37.53	<0.01
		3	39.39	<0.01

35	2	2	41.58	0.65
		3	76.87	5.48
		4	408.33	23.21
	3	2	41.58	<0.01
		3	69.92	6.31
		4	296.07	2.44

Whilst quaternary mixtures were also attempted to be calculated using 15 candidates at the given target, results failed in stability tests; same happened to 5-solvent mixtures using 35 candidates. This was because this specific target HSPs were close to the termini of the convex hull, especially the δ_D term was approaching the limit (Figure S 3). This made the calculation in this example more sensitive and reduced the number of successful predictions. The number of successful results in each calculation are listed in Table S 11.

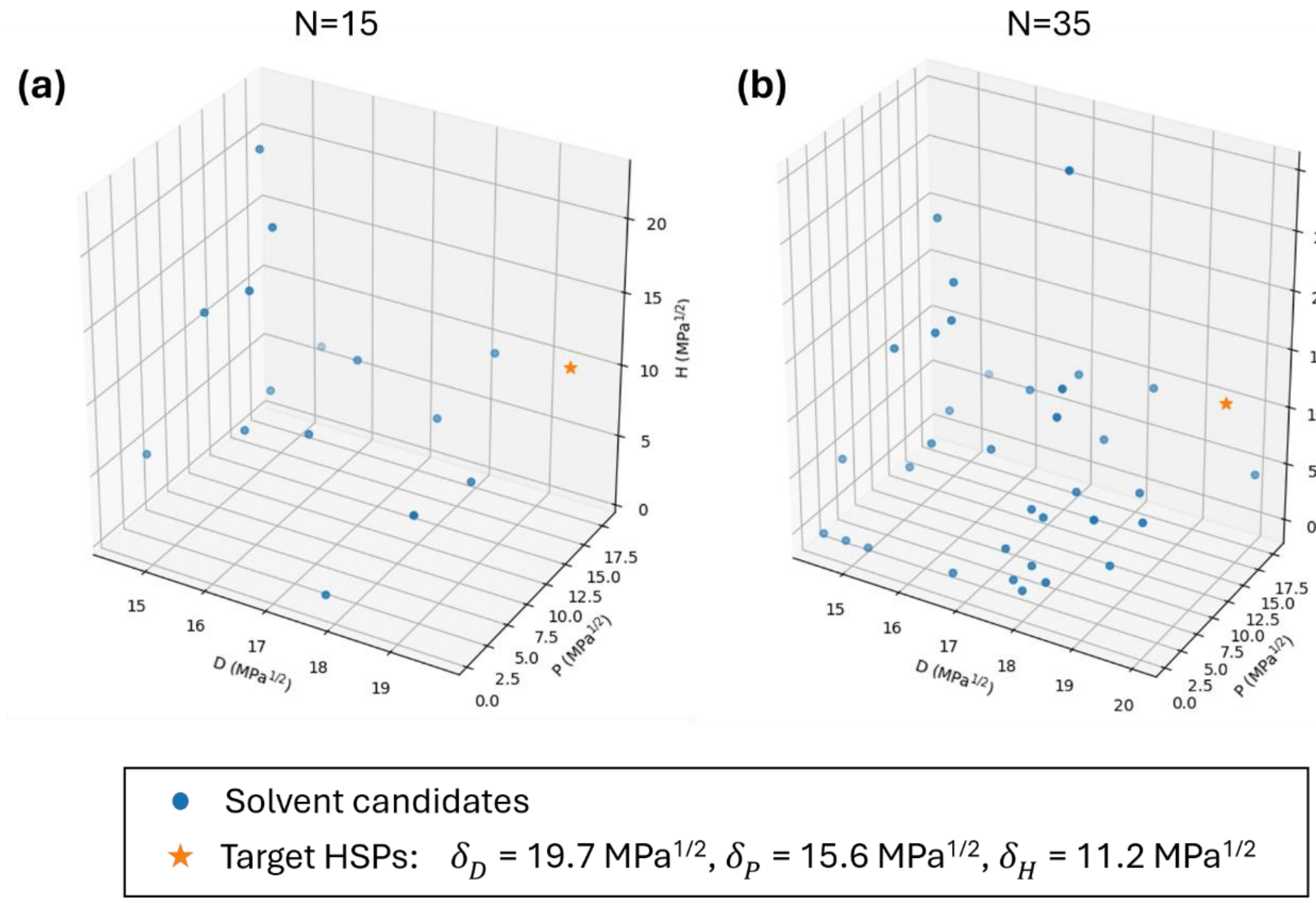

Figure S 3 Distribution of candidate solvents in the Hansen space with respect to the target (star). (a) 15 candidates; (b) 35 candidates. Target HSPs: δ_D = 19.7 MPa$^{1/2}$, δ_P = 15.6 MPa$^{1/2}$, and δ_H = 11.2 MPa$^{1/2}$.

Table S 11 Number of successful results of SolvPred for n=2 to 5 using 15, 35 and 249 candidates (for n=2 and 3 only). All errors = 2.0 MPa$^{1/2}$; redundant solvent threshold = 1%; target HSPs: δ_D = 19.7 MPa$^{1/2}$, δ_P = 15.6 MPa$^{1/2}$, and δ_H = 11.2 MPa$^{1/2}$.

Number of candidates (N)	Number of solvents (n)	Successful results after main calculation	Successful results after advanced filtering
15	2	12	12

	3	25	24
	4	0	0
	5	0	0
35	2	32	31
	3	204	201
	4	1	1
	5	0	0
249	2	737	719
	3	41,948	40,655

To evaluate the computing performance in the case of a target that is close to the center of a given candidate set, a new set of target HSPs were set as δ_D = 16.0 MPa$^{1/2}$, δ_P = 10.0 MPa$^{1/2}$, and δ_H = 10.0 MPa$^{1/2}$, respectively, according to the average of each dimension of HSPs in the 15- and 35-candidate sets. The error of each HSP dimension remained as 2.0 MPa$^{1/2}$; the redundant solvent threshold was set as 1%. The relative position of the new target with respect to candidate solvents is shown in Figure S 4.

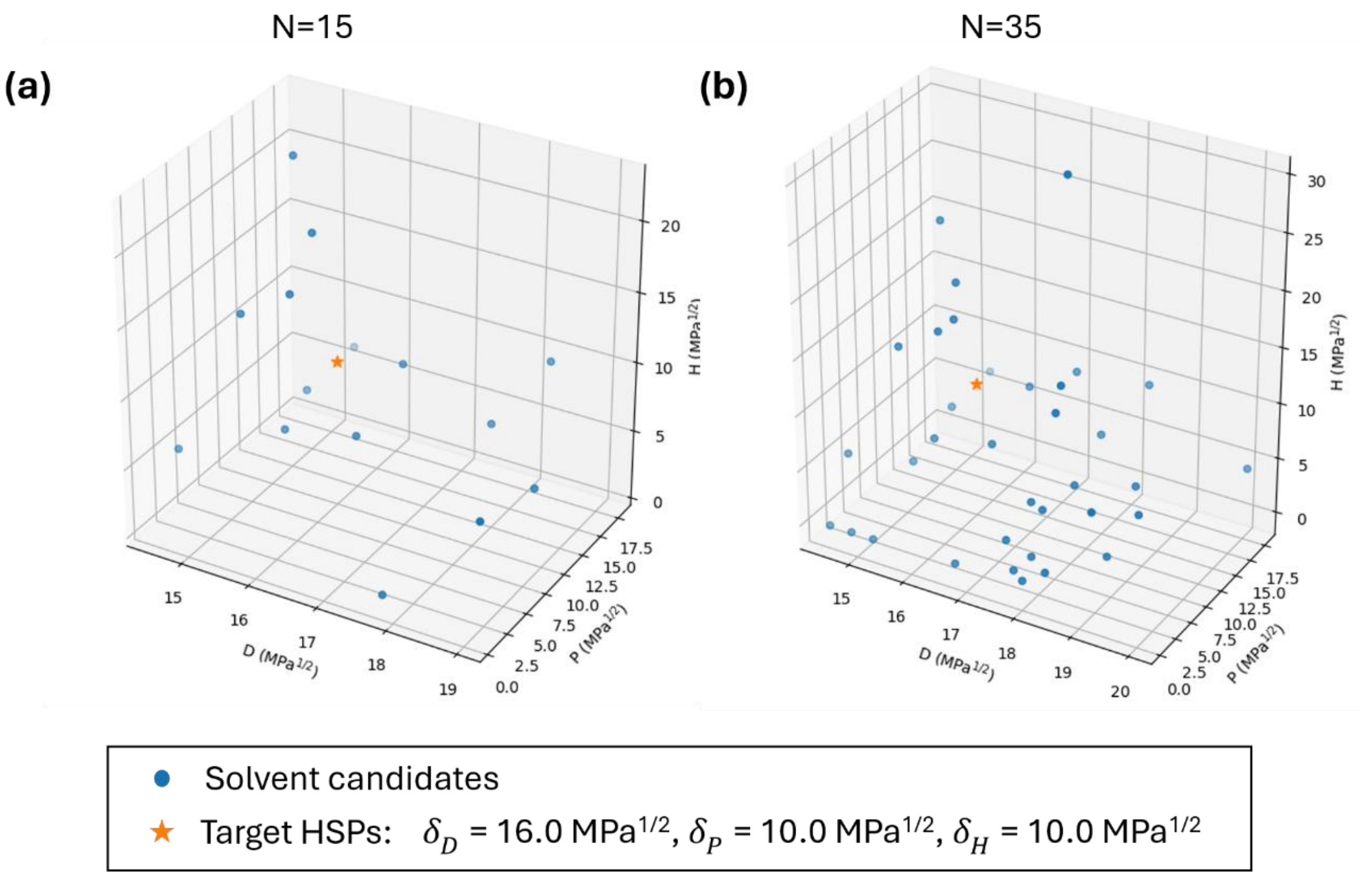

Figure S 4 Distribution of candidate solvents in the Hansen space with respect to the target (star). (a) 15 candidates; (b) 35 candidates. Target HSPs: δ_D = 16.0 MPa$^{1/2}$, δ_P = 10.0 MPa$^{1/2}$, and δ_H = 10.0 MPa$^{1/2}$.

With the new target now being closer to the center of candidates, n=2 to 5 all successfully yielded results (Table S 12). Calculations for the 5-solvent mixtures finished within 2 s using 15 candidate solvents, and within 3 minutes using 35 candidate solvents. Notably, when using the full solvent list as candidates, the memory usage for binary and ternary mixtures remained comparable with the case of previous target, but because the number of results increased (Table S 12), the runtime for Stage 3, especially for ternary mixtures,

increased accordingly. These results confirm that the overall runtime is affected by the relative position of the target HSPs and candidate solvents.

Table S 12 Details of memory usage and runtime of the calculation steps of SolvPred for n=2 to 5 using 15, 35 and 249 (for n=2 and 3 only) candidates. All errors = 2.0 MPa$^{1/2}$; redundant solvent threshold = 1%; target HSPs: δ_D = 16.0 MPa$^{1/2}$, δ_P = 10.0 MPa$^{1/2}$, and δ_H = 10.0 MPa$^{1/2}$.

Number of candidates (N)	Stage	Number of solvents (n)	Peak memory/MB	Runtime/s
15	2	2	38.73	0.22
		3	40.72	0.22
		4	53.78	0.77
		5	57.19	1.44
	3	2	38.73	<0.01
		3	40.72	<0.01
		4	53.14	0.11
		5	55.69	0.33
35	2	2	41.52	0.44
		3	75.00	2.88
		4	404.34	24.29
		5	2,560.54	167.89
	3	2	41.52	<0.01
		3	70.48	0.33
		4	303.02	1.32
		5	1,856.23	9.64
249	2	2	197.75	12.42
		3	8,602.20	4,416.07
	3	2	168.15	2.10
		3	5,772.05	31,045.40

Table S 13 Number of successful results of SolvPred for n=2 to 5 using 15, 35 and 249 candidates (for n=2 and 3 only). All errors = 2.0 MPa$^{1/2}$; redundant solvent threshold = 1%; target HSPs: δ_D = 16.0 MPa$^{1/2}$, δ_P = 10.0 MPa$^{1/2}$, and δ_H = 10.0 MPa$^{1/2}$.

Number of candidates (N)	Number of solvents (n)	Successful results after main calculation	Successful results after advanced filtering
15	2	19	19
	3	131	130
	4	159	159
	5	714	714
35	2	71	68
	3	792	753
	4	1,603	1,433
	5	17,232	15,113

249	2	3,607	3,445
	3	249,546	227,187

Using ternary mixtures, 35 solvent candidates and δ_D = 16.0 MPa$^{1/2}$, δ_P = 10.0 MPa$^{1/2}$, δ_H = 10.0 MPa$^{1/2}$, a sensitivity test was conducted to study the computing performance when hyperparameters, including the error of each HSP dimension and the redundant solvent threshold, are tuned to a finer resolution. The results suggest that the memory usage and runtime are independent of error or redundant solvent resolution, but the number of successful results can decrease when a tighter error criterion is applied or when a higher redundant solvent threshold is applied (Table S 14).

Table S 14 Details of memory usage and runtime of the calculation steps of SolvPred for n=3 using 35 candidates and different errors or redundant solvent thresholds, and resulting number of predictions. Target HSPs: δ_D = 16.0 MPa$^{1/2}$, δ_P = 10.0 MPa$^{1/2}$, and δ_H = 10.0 MPa$^{1/2}$.

Error for δ_D /MPa$^{1/2}$	Error for δ_P /MPa$^{1/2}$	Error for δ_H /MPa$^{1/2}$	Redundant solvent threshold/%	Stage	Peak memory /MB	Runtime/s	Successful results after main calculation	Successful results after advanced filtering
2.0	2.0	2.0	1.0	2	75.00	2.88	792	753
				3	70.48	0.33		
1.0	1.0	1.0	1.0	2	82.41	2.87	446	414
				3	75.35	0.11		
0.1	0.1	0.1	1.0	2	76.07	2.78	35	32
				3	76.07	<0.01		
2.0	2.0	2.0	0.1	2	73.67	2.76	808	768
				3	68.37	0.22		
1.0	1.0	1.0	0.1	2	75.05	2.76	449	417
				3	68.97	0.11		
0.1	0.1	0.1	0.1	2	74.15	2.85	36	33
				3	74.15	<0.01		
2.0	2.0	2.0	0.01	2	73.98	2.74	801	762
				3	69.12	0.44		
1.0	1.0	1.0	0.01	2	73.40	2.66	445	413
				3	68.59	0.22		
0.1	0.1	0.1	0.01	2	73.96	2.65	36	33
				3	67.31	0.11		

The sensitivity of the error of individual HSPs dimensions was then studied using 0.1% as redundant solvent threshold. Errors of three HSP dimensions were permutated among 2.0 MPa$^{1/2}$, 1.0 MPa$^{1/2}$, and 0.1 MPa$^{1/2}$. The outcomes were ranked by the number of successful results, listed in Table S 15. The most sensitive dimension was found as δ_D, as restricting its error to 0.1 MPa$^{1/2}$ yielded the least number of results. δ_P and δ_H showed a comparable sensitivity when δ_D was held consistent. This is because the scale of δ_D in

the candidate pool is narrower than δ_P and δ_H. In the full solvent database, δ_D ranges between 9.6 MPa$^{1/2}$ to 22.6 MPa$^{1/2}$, whereas δ_P and δ_H range from 0.0 MPa$^{1/2}$ to 52.0 MPa$^{1/2}$ and 0.0 MPa$^{1/2}$ to 54.0 MPa$^{1/2}$, respectively.

Table S 15 Sensitivity tests of different errors of HSP dimensions of SolvPred. Fixed conditions: 35 candidates, redundant solvent thresholds=0.1%. Target HSPs: δ_D = 16.0 MPa$^{1/2}$, δ_P = 10.0 MPa$^{1/2}$, and δ_H = 10.0 MPa$^{1/2}$.

Error for δ_D /MPa$^{1/2}$	Error for δ_P /MPa$^{1/2}$	Error for δ_H /MPa$^{1/2}$	Successful results after main calculation	Successful results after advanced filtering
2.0	2.0	2.0	808	768
2.0	1.0	2.0	743	703
2.0	1.0	1.0	735	696
2.0	2.0	1.0	729	690
1.0	1.0	2.0	463	429
1.0	1.0	1.0	449	417
1.0	2.0	2.0	448	416
1.0	2.0	1.0	445	413
2.0	1.0	0.1	58	54
2.0	2.0	0.1	57	54
2.0	0.1	2.0	57	54
2.0	0.1	1.0	57	54
1.0	0.1	2.0	57	54
1.0	0.1	1.0	57	54
1.0	1.0	0.1	56	52
1.0	0.1	0.1	55	51
2.0	0.1	0.1	53	49
1.0	2.0	0.1	53	48
0.1	2.0	2.0	37	34
0.1	2.0	0.1	37	34
0.1	1.0	1.0	37	34
0.1	0.1	1.0	37	34
0.1	1.0	2.0	36	33
0.1	1.0	0.1	36	33
0.1	0.1	2.0	36	33
0.1	0.1	0.1	36	33
0.1	2.0	1.0	35	32

S2 Supporting information for experimental case study

S2.1 Chemicals

Chemicals used for the synthesis and characterization of **pPI-1** in the case study are listed in Table S 16. All chemicals were used as received.

*Table S 16 Details of chemicals used for the synthesis and characterization of **pPI-1**.*

Name	Assay	Suppliers
1-Methylimidazole	99%	Sigma-Aldrich
Perylene-3,4,9,10- tetracarboxylic dianhydride	97%	Sigma-Aldrich
Melamine	99%	Thermo Scientific
Methanol	≥ 99.9%	Sigma-Aldrich
Propylene carbonate	99.7%	Sigma-Aldrich
Triethylamine	≥ 99%	Fisher Chemical
Dimethyl sulfoxide	≥ 99.9%	Thermo Scientific
Dimethylformamide	99%	Fisher Chemical
tert-Butyl alcohol	≥ 99.5%	Sigma-Aldrich
Acetone	≥ 99.5%	Sigma-Aldrich
N-methyl-2-pyrrolidone	≥ 99.5%	BioSolv
Zinc acetate	99.9%	Thermo Scientific
Barium sulfate	99%	Merck

S2.2 Synthesis of **pPI-1** in solvents with varied δ_H

pPI-1 was synthesized and characterized according to a literature-reported procedure.[9,10] The solvents used for the synthesis of **pPI-1** adopted a single solvent control group, 1-methylimidazole (NMI), and were modified to ternary mixtures that retained the same δ_D (19.7 MPa$^{1/2}$) and δ_P (15.6 MPa$^{1/2}$) as NMI, with δ_H finely controlled from 5 MPa$^{1/2}$ to 9 MPa$^{1/2}$ at a 2 MPa$^{1/2}$ interval. s

pPI-1 was formed *via* a polyimide condensation reaction of PTCDA (0.76 mmol) and melamine (0.38 mmol), with 40 mL 1-methylimidazole or mixed solvents, and zinc acetate ($Zn(OAc)_2$) (0.96 mmol) as a catalyst. The reaction in ternary solvent mixtures was conducted at 110 °C, with a condenser attached. Solvent mixtures used for the optimization of this reaction were predicted using the *SolvPred* workflow. The product was purified by Soxhlet extractions to ensure the complete removal of starting materials, catalysts and oligomers.

The general synthesis procedure in the case study was:

A dried round-bottomed flask equipped with mechanical stirring and a reflux condenser was charged with 1-methylimidazole (NMI) or mixed solvents (40 ml), perylene-3,4,9,10-

tetracarboxylic dianhydride (PTCDA) (297 mg, 0.76 mmol), melamine (47.9 mg, 0.38 mmol), and zinc acetate ($Zn(OAc)_2$) (211.28 mg, 0.96 mmol) as catalyst. The reaction mixture was stirred and heated at 110 °C for 72 hours. After cooling to room temperature, methanol (MeOH) (50 ml) was added and the precipitate was collected and washed with additional DMF and MeOH, and acetone (50 ml x 3, each). The product was dried under vacuum at 80 °C overnight, yielding the polymer product.

In the control group where a single solvent, 1-methylimidazole, was applied, the reaction yielded an average of 33% products at 180 ℃, and 16% at 110 ℃. All yields are reported as separated yields of polymers.

The proposed mechanism is provided in Scheme S 54.

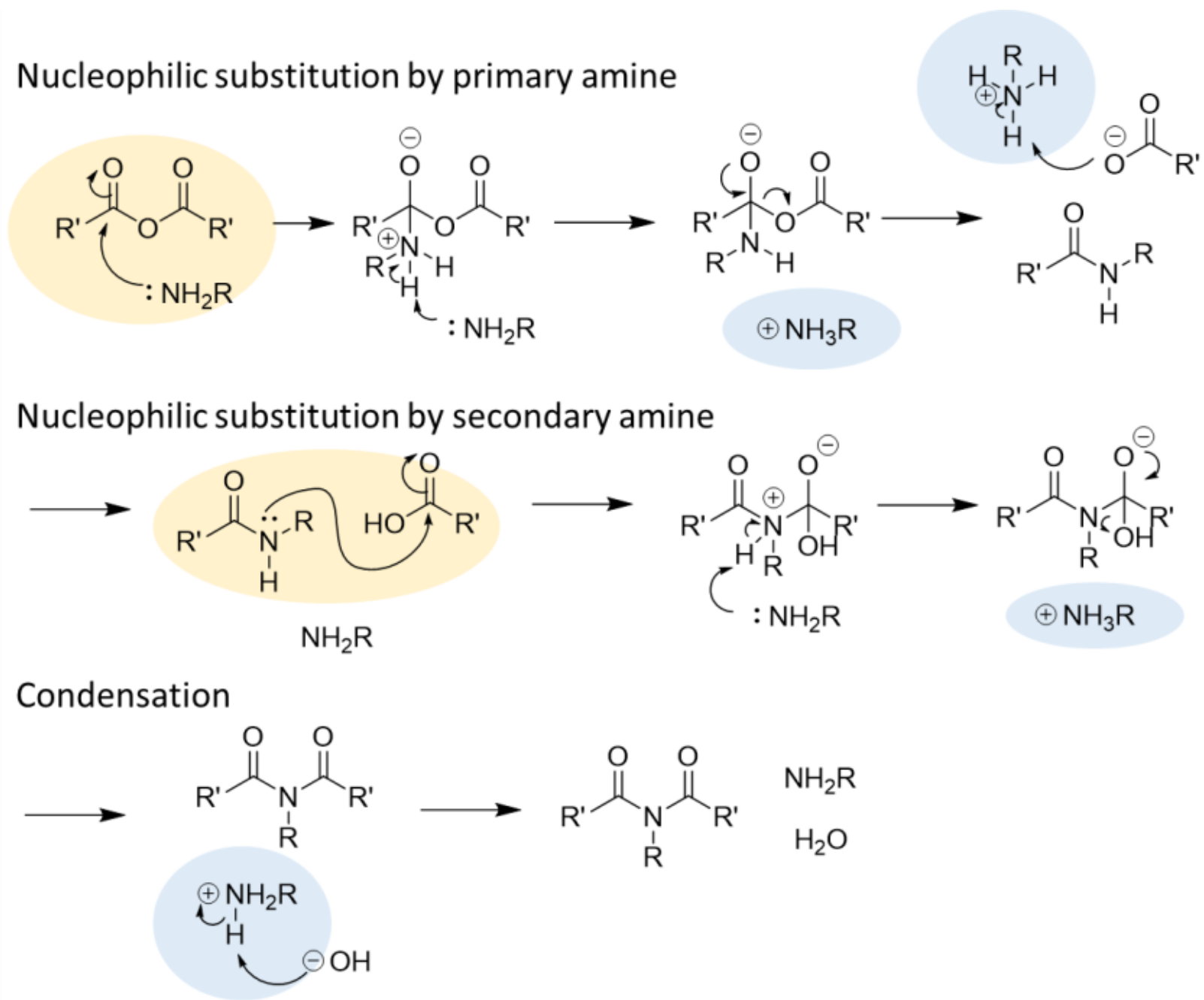

Scheme S 54 The proposed mechanism of the polyimide condensation reaction. Steps highlighted in yellow are nucleophilic attack. Steps highlighted in blue are procedures involving ammonium intermediates.

<u>*Solvent prediction methods:*</u>

For the mixed solvent groups, the mixed solvents were prepared from a combination of three solvents in different ratios to regulate δ_H of their combined Hansen Solubility Parameters. Ternary solvent mixtures were constructed from the following candidate list: propylene carbonate (PC), triethylamine (TEA), dimethyl sulfoxide (DMSO), dimethylformamide (DMF), *tert*-butyl alcohol (*t*BuOH), methyl-2-pyrrolidone (NMP), acetonitrile (ACN), with their HSPs, dielectric constants (ε) and boiling points (b.p.) listed in Table S 17. These solvents were selected for their moderately high boiling points, good chemical compatibility with reaction species, and the majority are polar solvents that are

expected to facilitate the nucleophilic attack involved in this reaction, except for TEA that contributes to the basicity of the solvent environment.

Table S 17 HSPs, dielectric constants and boiling points of solvent candidates used for the co-solvent prediction for pPI-1 synthesis.

Solvent	δ_D /MPa$^{1/2}$	δ_P /MPa$^{1/2}$	δ_H /MPa$^{1/2}$	ε	B.p. /℃
1-Methylimidazole (NMI)	19.7	15.6	11.2	33	198
Dimethylsulfoxide (DMSO)	18.4	16.4	10.2	47	189
Dimethylformamide (DMF)	17.4	13.7	11.3	38	153
Propylene carbonate (PC)	20.0	18.0	4.1	64	242
Methyl-2-pyrrolidone (NMP)	18.0	12.3	7.2	33	202
Acetonitrile (ACN)	15.3	18.0	6.1	38	82
tert-butyl alcohol (*t*BuOH)	15.2	5.1	14.7	11	82
Triethylamine (TEA)	17.8	0.4	1.0	2.4	88

The co-solvent mixtures were predicted using the *SolvPred* workflow with the following parameters: number of co-solvents: 3, tolerance of error for each HSP: 2.0 MPa$^{1/2}$, tolerance of redundant solvents: 0.01. Target δ_H were set as 9.0 MPa$^{1/2}$, 7.0 MPa$^{1/2}$ and 5.0 MPa$^{1/2}$. The target HSPs, predicted combinations and resulting yields are listed in Table 1. Target δ_D and target δ_P are 19.7 MPa$^{1/2}$ and 15.6 MPa$^{1/2}$, respectively, in all cases.

Ternary solvent experiments without TEA being a co-solvent:

Two control groups of δ_H set to 9.0 MPa$^{1/2}$ (Group 9N) and 7.0 MPa$^{1/2}$ (Group 7N) were carried out without the presence of TEA (Table S 18). Reactions did not form pure isolatable polymeric products in either of these solvent mixtures. Note that no mixtures could be predicted when δ_H equals to 5.0 MPa$^{1/2}$ if TEA is excluded, due to a lack of low polar co-solvent.

Table S 18 Target HSPs, predicted mixtures of constructed by solvents without TEA and reaction outcome (separated yield), All reactions were carried out at 110 ℃.

Entry	Target δ_H/ MPa$^{1/2}$	Solvent 1	Solvent 2	Solvent 3	δ_D /MPa$^{1/2}$[a]	δ_P /MPa$^{1/2}$[a]	δ_H /MPa$^{1/2}$[a]	Yield/ %[b]
9N	9.0	48% PC	14% *t*BuOH	38% DMF	18.3	14.5	8.3	NC
7N	7.0	76% PC	24% *t*BuOH	-	18.8	14.9	6.6	NC

[a] The systematic variation (from 0.2 MPa1/2 to 0.9 MPa1/2) in actual δD and δP arises from the error specified (2.0 MPa1/2). The actual HSPs predicted by *SolvPred* fall within the user-specified errors [b] The yield% refers to the isolated yield of pure polymeric products – the reactions in 9N, 7N proceed

to form impure mixtures of oligomeric species stacked with starting reagent PTCDA, hence denoted "NC" here for "not clean", where no valid yield was obtained due to such impurities. Each experiment has been independently performed twice.

A breakdown of calculations for each entry is provided below:

Entry 9A:

Table S 19 A breakdown of HSPs calculation of mixtures (Entry 9A).

Solvent	δ_D /MPa$^{1/2}$	δ_P /MPa$^{1/2}$	δ_H /MPa$^{1/2}$	c%
solvent 1 = PC	20.0	18.0	4.1	11%
solvent 2 = TEA	17.8	0.4	1.0	12%
solvent 3 = DMSO	18.4	16.4	10.2	77%
**total**	18.5	14.7	8.4	

δ_D = 11% * 20.0 + 12% * 17.8 + 77% * 18.4 = 18.5 MPa$^{1/2}$

δ_P = 11% * 18.0 + 12% * 0.4 + 77% * 16.4 = 14.7 MPa$^{1/2}$

δ_H = 11% * 4.1 + 12% * 1.0 + 77% * 10.2 = 8.4 MPa$^{1/2}$

Entry 9B:

Table S 20 A breakdown of HSPs calculation of mixtures (Entry 9B).

Solvent	δ_D /MPa$^{1/2}$	δ_P /MPa$^{1/2}$	δ_H /MPa$^{1/2}$	c%
solvent 1 = PC	20.0	18.0	4.1	34%
solvent 2 = TEA	17.8	0.4	1.0	5%
solvent 3 = DMF	17.4	13.7	11.3	61%
**total**	18.3	14.5	8.3	

δ_D = 34% * 20.0 + 5% * 17.8 + 61% * 17.4 = 18.3 MPa$^{1/2}$

δ_P = 34% * 18.0 + 5% * 0.4 + 61% * 13.7 = 14.5 MPa$^{1/2}$

δ_H = 34% * 4.1 + 5% * 1.0 + 61% * 11.3 = 8.3 MPa$^{1/2}$

Entry 7A:

Table S 21 A breakdown of HSPs calculation of mixtures (Entry 7A).

Solvent	δ_D /MPa$^{1/2}$	δ_P /MPa$^{1/2}$	δ_H /MPa$^{1/2}$	c%
solvent 1 = PC	20.0	18.0	4.1	38%
solvent 2 = TEA	17.8	0.4	1.0	13%

solvent 3 = DMSO	18.4	16.4	10.2	49%
**total**	18.9	15.0	6.7	

δ_D = 38% * 20.0 + 13% * 17.8 + 49% * 18.4 = 18.9 MPa$^{1/2}$

δ_P = 38% * 18.0 + 13% * 0.4 + 49% * 16.4 = 15.0 MPa$^{1/2}$

δ_H = 38% * 4.1 + 13% * 1.0 + 49% * 10.2 = 6.7 MPa$^{1/2}$

Entry 7B:

Table S 22 A breakdown of HSPs calculation of mixtures (Entry 7B).

Solvent	δ_D /MPa$^{1/2}$	δ_P /MPa$^{1/2}$	δ_H /MPa$^{1/2}$	c%
solvent 1 = PC	20.0	18.0	4.1	53%
solvent 2 = TEA	17.8	0.4	1.0	8%
solvent 3 = DMF	17.4	13.7	11.3	39%
**total**	18.8	14.9	6.7	

δ_D = 53% * 20.0 + 8% * 17.8 + 39% * 17.4 = 18.8 MPa$^{1/2}$

δ_P = 53% * 18.0 + 8% * 0.4 + 39% * 13.7 = 14.9 MPa$^{1/2}$

δ_H = 53% * 4.1 + 8% * 1.0 + 39% * 11.3 = 6.7 MPa$^{1/2}$

Entry 5A:

Table S 23 A breakdown of HSPs calculation of mixtures (Entry 5A).

Solvent	δ_D /MPa$^{1/2}$	δ_P /MPa$^{1/2}$	δ_H /MPa$^{1/2}$	c%
solvent 1 = PC	20.0	18.0	4.1	67%
solvent 2 = TEA	17.8	0.4	1.0	13%
solvent 3 = DMSO	18.4	16.4	10.2	20%
**total**	19.4	15.4	4.9	

δ_D = 67% * 20.0 + 13% * 17.8 + 20% * 18.4 = 19.4 MPa$^{1/2}$

δ_P = 67% * 18.0 + 13% * 0.4 + 20% * 16.4 = 15.4 MPa$^{1/2}$

δ_H = 67% * 4.1 + 13% * 1.0 + 20% * 10.2 = 4.9 MPa$^{1/2}$

Entry 5B:

Table S 24 A breakdown of HSPs calculation of mixtures (Entry 5B).

Solvent	δ_D /MPa$^{1/2}$	δ_P /MPa$^{1/2}$	δ_H /MPa$^{1/2}$	c%
solvent 1 = PC	20.0	18.0	4.1	73%

solvent 2 = TEA	17.8	0.4	1.0	11%
solvent 3 = DMF	17.4	13.7	11.3	16%
**total**	19.3	15.4	4.9	

δ_D = 73% * 20.0 + 11% * 17.8 + 16% * 17.4 = 19.3 MPa$^{1/2}$

δ_P = 73% * 18.0 + 11% * 0.4 + 16% * 13.7 = 15.3 MPa$^{1/2}$

δ_H = 73% * 4.1 + 11% * 1.0 + 16% * 11.3 = 4.9 MPa$^{1/2}$

Entry 5C:

Table S 25 A breakdown of HSPs calculation of mixtures (Entry 5C).

Solvent	δ_D /MPa$^{1/2}$	δ_P /MPa$^{1/2}$	δ_H /MPa$^{1/2}$	c%
solvent 1 = PC	20.0	18.0	4.1	64%
solvent 2 = TEA	17.8	0.4	1.0	5%
solvent 3 = NMP	18.0	12.3	7.2	31%
**total**	19.3	15.4	4.9	

δ_D = 64% * 20.0 + 5% * 17.8 + 31% * 18.0 = 19.3 MPa$^{1/2}$

δ_P = 64% * 18.0 + 5% * 0.4 + 31% * 12.3 = 15.3 MPa$^{1/2}$

δ_H = 64% * 4.1 + 5% * 1.0 + 31% * 7.2 = 4.9 MPa$^{1/2}$

Entry 9N:

Table S 26 A breakdown of HSPs calculation of mixtures (Entry 9N).

Solvent	δ_D /MPa$^{1/2}$	δ_P /MPa$^{1/2}$	δ_H /MPa$^{1/2}$	c%
solvent 1 = PC	20.0	18.0	4.1	48%
solvent 2 = *t*BuOH	15.2	5.1	14.7	14%
solvent 3 = DMF	17.4	13.7	11.3	38%
**total**	18.3	14.5	8.3	

δ_D = 48% * 20.0 + 14% * 15.2 + 38% * 17.4 = 18.3 MPa$^{1/2}$

δ_P = 48% * 18.0 + 14% * 5.1 + 38% * 13.7 = 14.5 MPa$^{1/2}$

δ_H = 48% * 4.1 + 14% * 14.7 + 38% * 11.3 = 8.3 MPa$^{1/2}$

Entry 7N:

Table S 27 A breakdown of HSPs calculation of mixtures (Entry 7N).

Solvent	δ_D /MPa$^{1/2}$	δ_P /MPa$^{1/2}$	δ_H /MPa$^{1/2}$	c%
solvent 1 = PC	20.0	18.0	4.1	76%
solvent 2 = *t*BuOH	15.2	5.1	14.7	24%
**total**	18.8	14.9	6.6	

$$\delta_D = 76\% * 20.0 + 24\% * 15.2 = 18.8 \text{ MPa}^{1/2}$$

$$\delta_P = 76\% * 18.0 + 24\% * 5.1 = 14.9 \text{ MPa}^{1/2}$$

$$\delta_H = 76\% * 4.1 + 24\% * 14.7 = 6.6 \text{ MPa}^{1/2}$$

The distribution of yield versus HSPs of predicted solvent mixtures

While a systematic variation presents in the δ_D and δ_P dimensions, ranging from 0.2 MPa$^{1/2}$ to 0.9 MPa$^{1/2}$ that fell within the error thresholds, these data points cluster most closely in the δ_D - δ_P space (Figure S 5a) compared to the δ_P - δ_H (Figure S 5b) and δ_D - δ_H space (Figure S 5c). Therefore, δ_H is still the primary parameter which changes here.

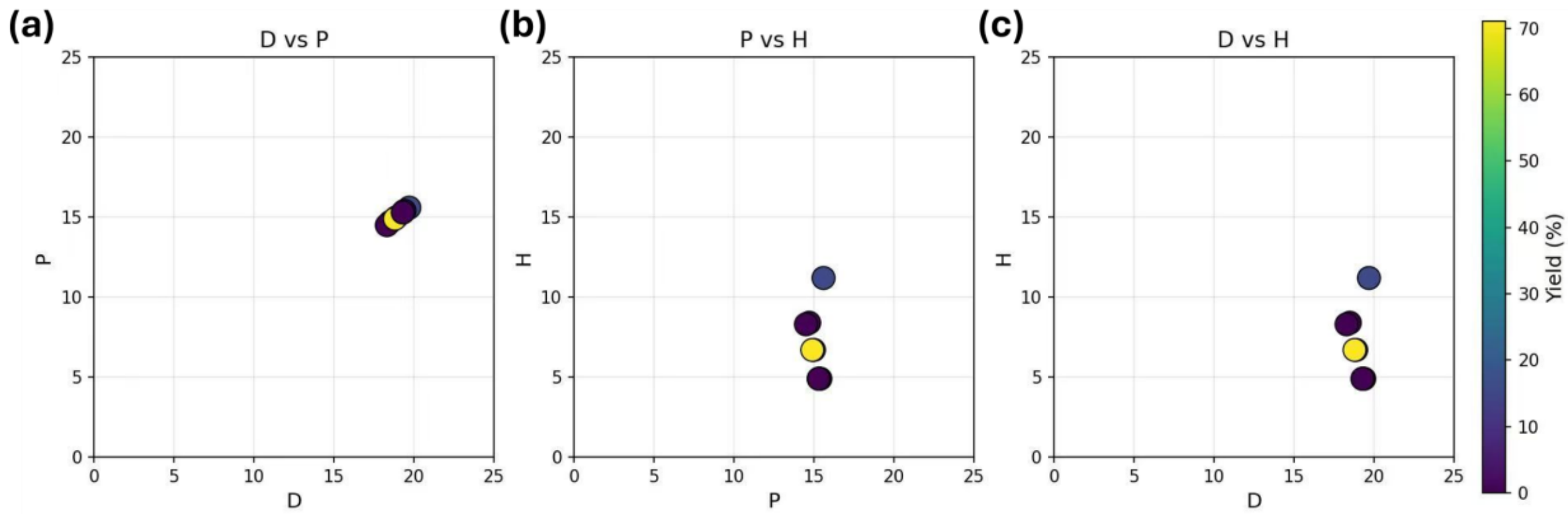

Figure S 5 Distribution of yield versus HSPs of predicted solvent mixtures in the (a) δ_D and δ_P space; (b) δ_D and δ_P space and (c) δ_D and δ_H space.

Further quantitative analysis based on first order or second order regression was not conducted because the clustering of a relatively small number of data points in a three-variable problem may not yield a valid outcome. However, the sensitivity of each HSP dimension in different applications may vary from case to case, so users should take this into consideration.

S2.3 Characterization

The product was characterized by Fourier transform infrared (FT–IR) spectroscopy, ultra violet–visible–near infrared (UV–Vis–NIR) spectrometry and powder X-ray diffraction

(PXRD) to confirm the successful formation of polymer network. FT-IR spectroscopy was recorded using a PerkinElmer Spectrum Two IR Spectrometer. PXRD was measured using a PSD LynxEye detector-equipped Bruker D8 Advance diffractometer. UV–Vis–NIR spectra were measured using a Shimadzu UV2600 spectrophotometer.

FT–IR spectra of the starting materials (PTCDA and melamine) and pure powder product of **pPI-1** samples were measured using a PerkinElmer Spectrum Two IR Spectrometer equipped with a UATR Single Reflection Diamond attenuated total reflectance (ATR) attachment. The wavenumber range in which the spectra was taken was 4000 cm^{-1} to 400 cm^{-1}.

A Shimadzu UV2600 spectrophotometer equipped with an ISR2600Plus integrating sphere attachment was used to record the UV–Vis–NIR spectra of the starting materials and **pPIs** (3 wt%) mixed with barium sulphate ($BaSO_4$). The spectra were acquired between 220 nm and 1400 nm.

A PSD LynxEye detector-equipped Bruker D8 Advance diffractometer was used to acquire the PXRD patterns. The device used Cu Kα radiation with a wavelength of 1.54 Å and operated at 40 kV and 40 mA. 5° to 60° (2-theta) was the range of angles that were investigated, with a step size of 0.02° and a step time of 1 second. To ensure homogeneity of the recorded samples, samples were rotated at a speed of 60 revolutions per minute (rpm).

According to FT–IR results (Figure S 6), the complete condensation of amine moieties to form **pPI-1** is confirmed by the absence of -NH stretching signals (3460–3213 cm^{-1}) from the starting material. The triazine units present in products are confirmed by the signal at 1446–1490 cm^{-1}.

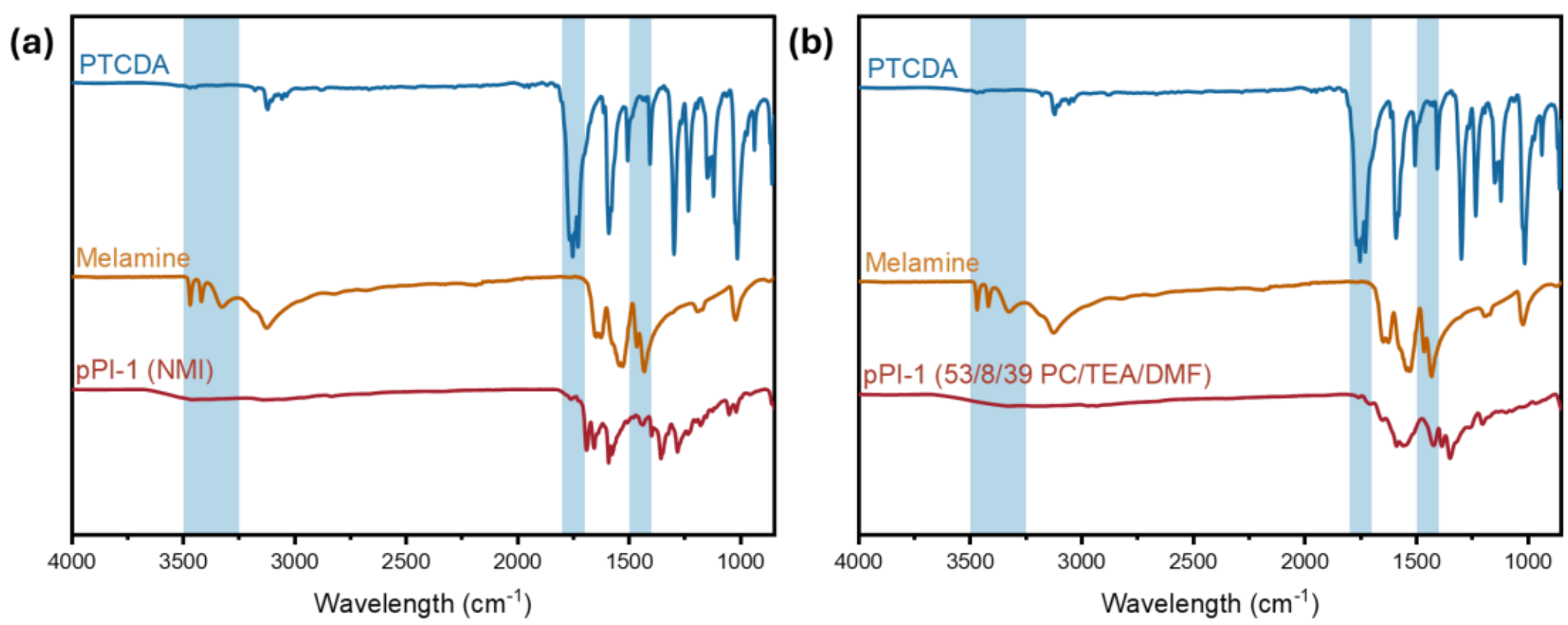

Figure S 6 FT–IR characterization of PTCDA (blue), melamine (yellow), ***pPI-1*** *(red) synthesized in (a) single solvent NMI control group and (b) co-solvent mixtures.*

The FT–IR results align well with UV–Vis–NIR spectra. Compared to starting materials, where sharp absorption peaks below 300 nm (melamine) and around 550 nm (PTCDA)

are found, the absorption characteristics of **pPI-1** extend broadly in the visible region and beyond 1400 nm, indicating the production of products with extended conjugation. In addition, a shoulder at *ca*. 780 nm (highlighted in blue) is found in **pPI-1** synthesized in both the single solvent case and the co-solvent mixtures.

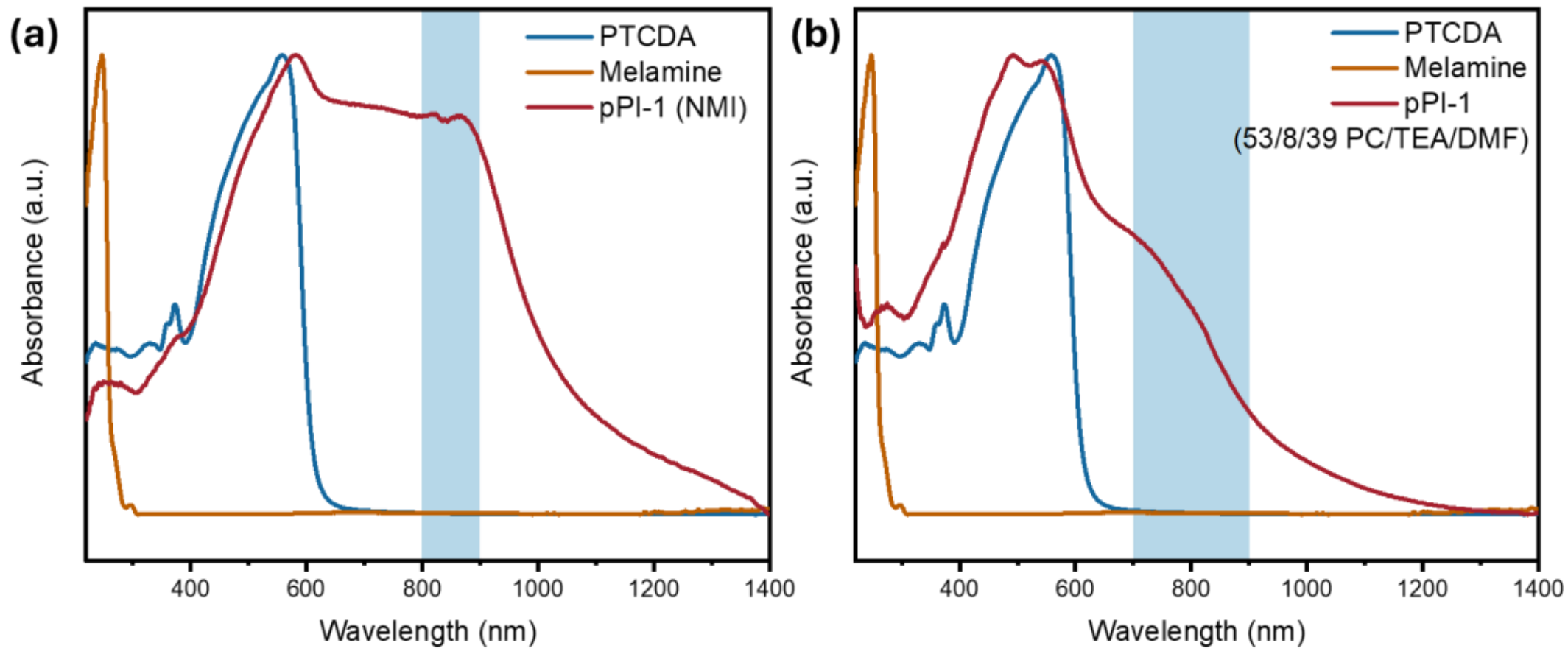

Figure S 7 UV–Vis–NIR spectra of PTCDA (blue), melamine (yellow) and ***pPI-1*** *(red) synthesized in (a) NMI and (b) co-solvent mixtures.*

All isolated polymers were characterized using PXRD to confirm the complete removal of starting materials and oligomers, along with the successful formation of polymers. Results are compared with reference PXRD spectra of melamine and PTCDA (Figure S 8) to ensure no corresponding patterns exist in the PXRD spectra of **pPI-1** products.

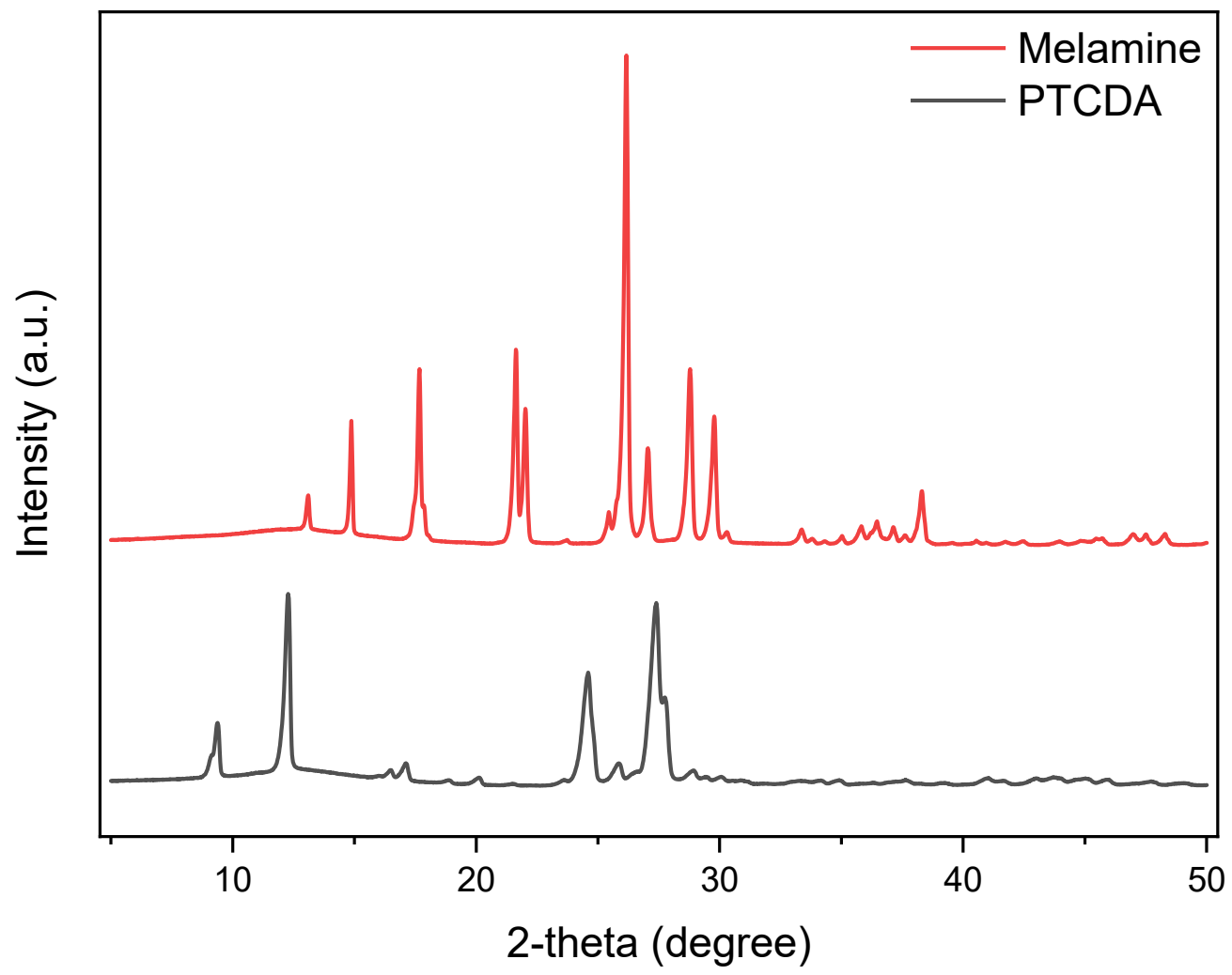

Figure S 8 PXRD spectra of melamine (red) and PTCDA (black).

PXRD spectra of **pPI-1** synthesized in the NMI control group, PC/TEA/DMF mixtures of target δ_H = 9.0 MPa$^{1/2}$, 7.0 MPa$^{1/2}$ and 5.0 MPa$^{1/2}$ are shown in Figure S 9. The target δ_H = 9.0 MPa$^{1/2}$ group, while crude product was obtained, did not yield pure polymers due to

the unremovable PTCDA monomers that present at 12°, 25°, and 28° in the PXRD spectrum. Pure **pPI-1** polymers were confirmed in the rest of the cases.

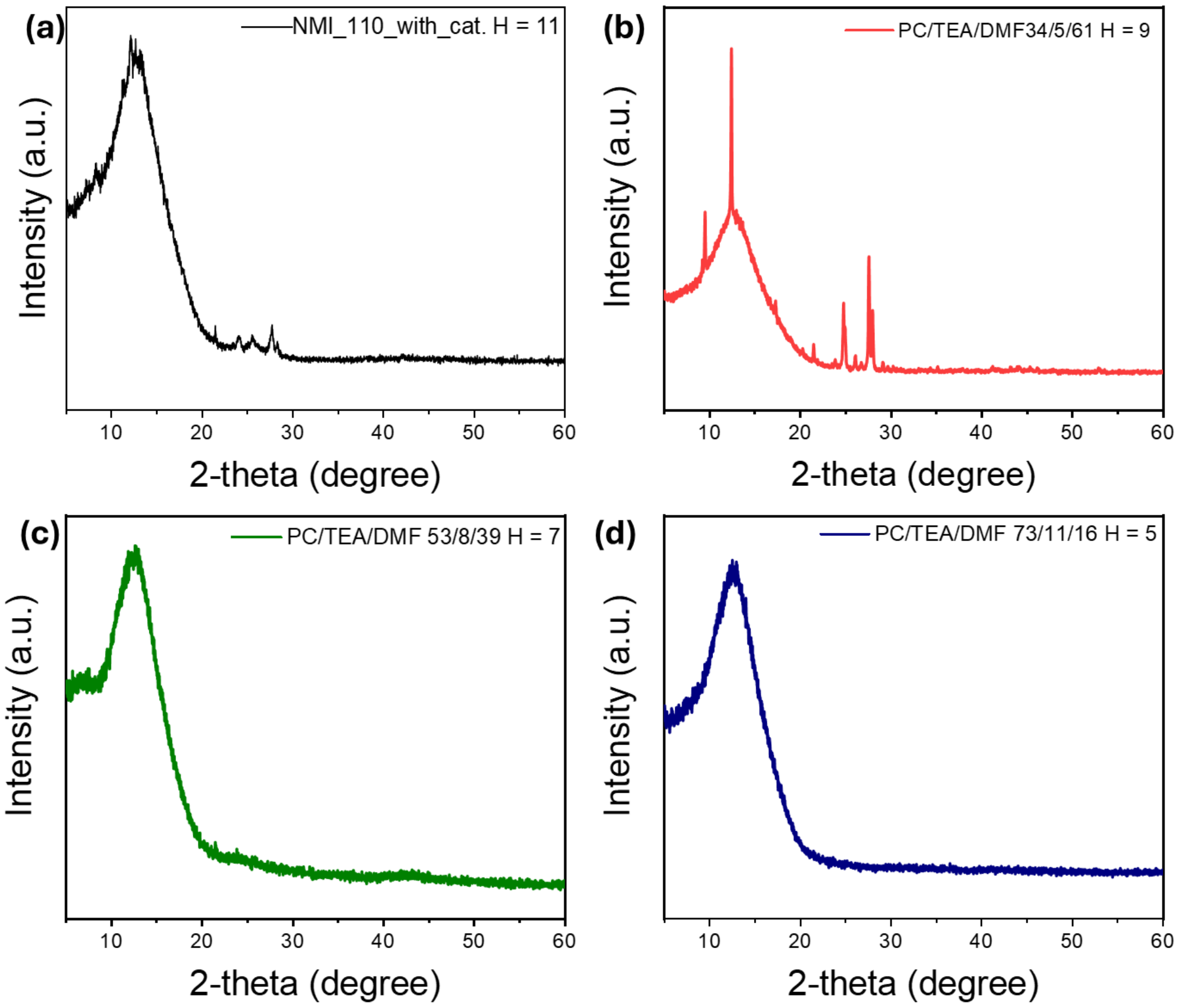

*Figure S 9 PXRD characterisation of **pPI-1** synthesized in (a) NMI and the PC, TEA, DMF mixture series with (b) target δ_H = 9.0 MPa$^{1/2}$, (c) 7.0 MPa$^{1/2}$ and (d) 5.0 MPa$^{1/2}$ The yield obtained in subplot (b) is regarded as invalid due to the unremovable PTCDA monomers signals at 12°, 25°, and 28°.*

PXRD results of PC/TEA/DMSO mixtures at target δ_H = 9.0 MPa$^{1/2}$, 7.0 MPa$^{1/2}$ and 5.0 MPa$^{1/2}$ are shown in Figure S 10a-c. Pure **pPI-1** polymers were only obtained at target δ_H = 7.0 MPa$^{1/2}$ (Figure S 10b). Using other solvents, characteristic peaks of PTCDA were found in the PXRD spectra. In the PC/TEA/NMP group predicted at target δ_H = 5.0 MPa$^{1/2}$ (Figure S 10d), no pure **pPI-1** polymers were obtained, either.

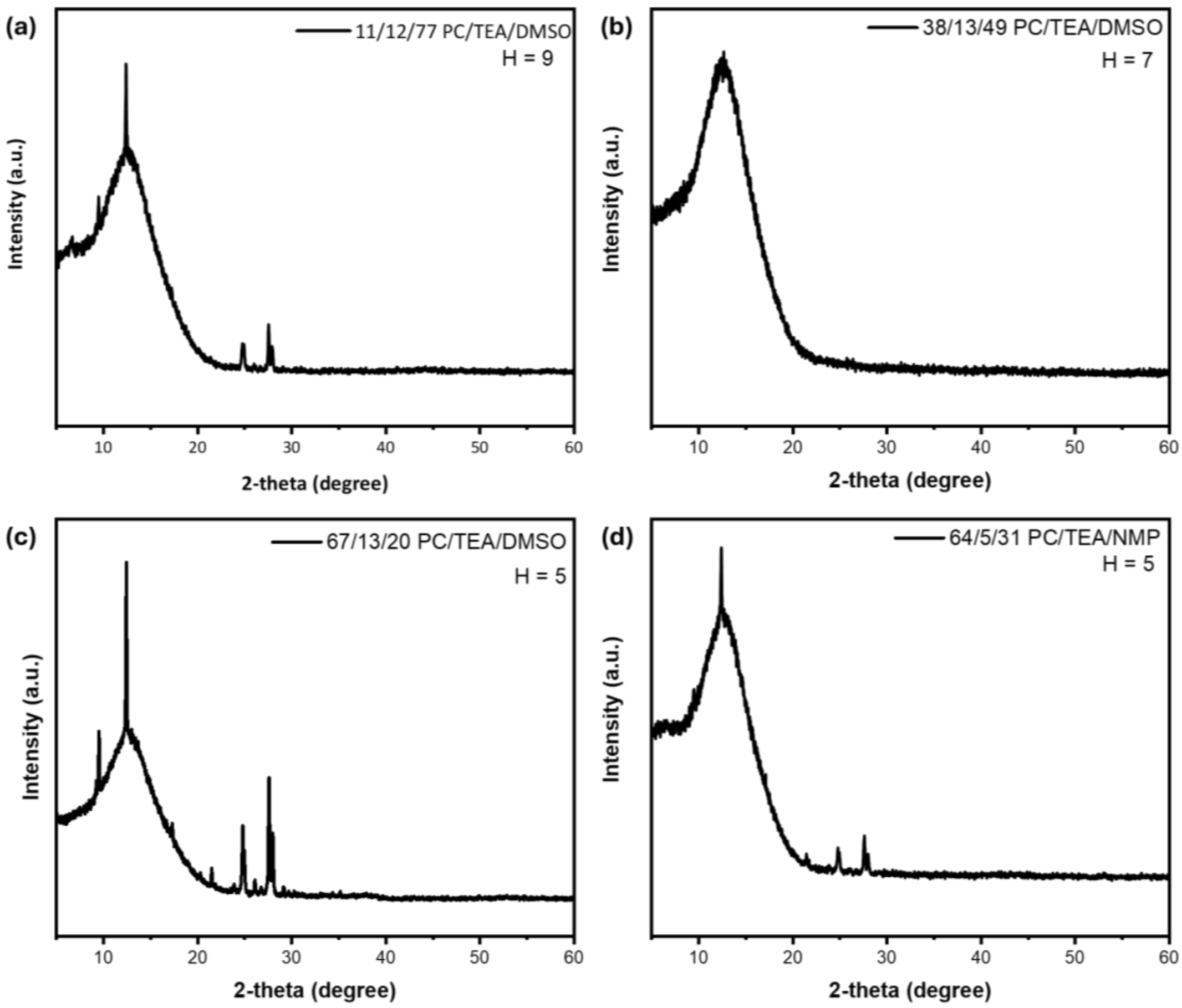

*Figure S 10 PXRD characterisation of **pPI-1** synthesized in the PC/TEA/DMSO series at target δ_H = (a) 9.0 MPa$^{1/2}$, (b) 7.0 MPa$^{1/2}$ and (c) 5.0 MPa$^{1/2}$ and (d) the PC/TEA/NMP mixture at target δ_H = 5.0 MPa$^{1/2}$. Subplots (a), (c), (d) did not yield valid pure polymer products due to co-existence of PTCDA.*

When TEA was excluded, no pure **pPI-1** could be obtained either at target δ_H = 9.0 MPa$^{1/2}$ (Figure S 11a) or 7.0 MPa$^{1/2}$ (Figure S 11b), suggesting the crucial role of a basic co-solvent in this reaction setup.

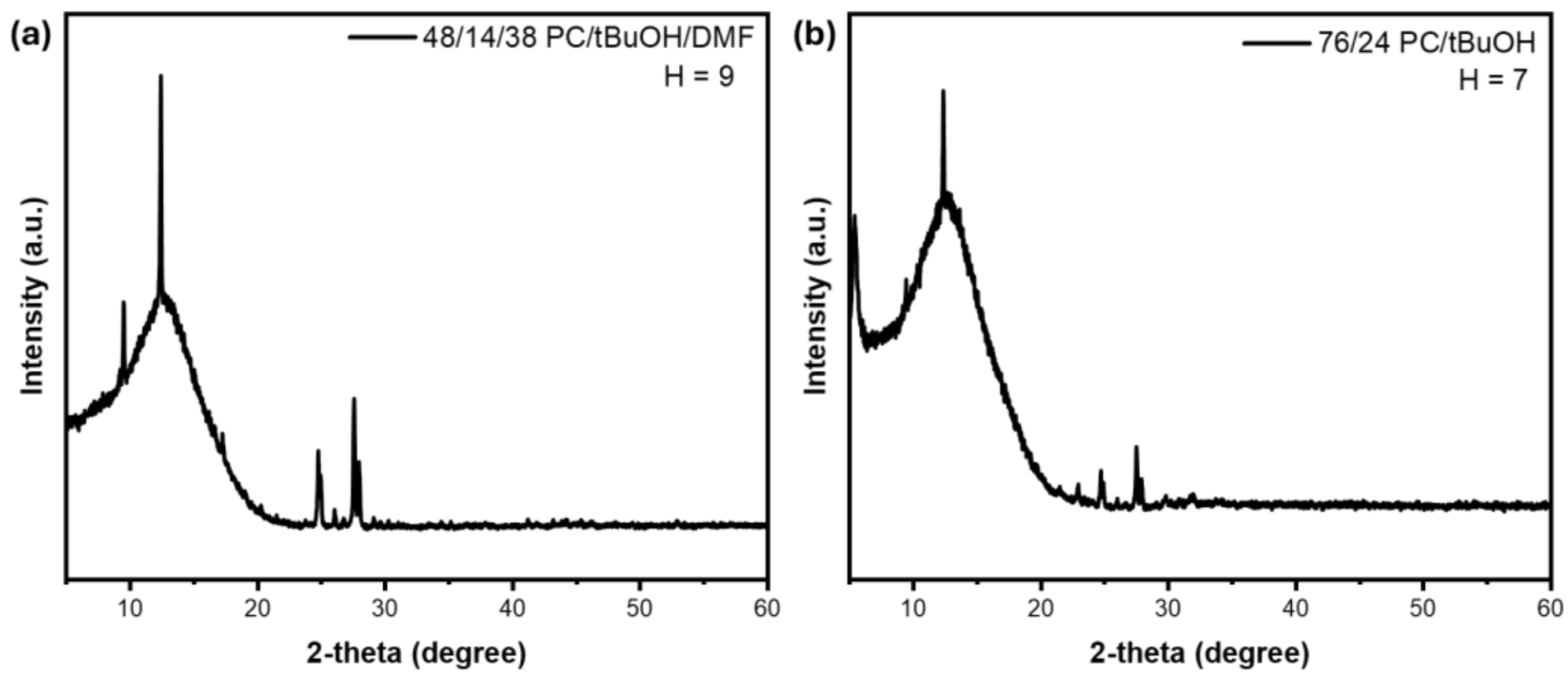

*Figure S 11 PXRD characterisation of **pPI-1** synthesized in the absence of TEA. (a) 48/14/38 PC/tBuOH/DMF, target δ_H = 9.0 MPa$^{1/2}$; (b) 76/24 PC/tBuOH, target δ_H = 7.0 MPa$^{1/2}$. No pure **pPI-1** was obtained in either case.*

S2.4 Economic impact evaluation of predicted solvent mixtures

We noticed the cost of solvent mixtures predicted by *SolvPred* was significantly reduced compared to the original process. Prices of all solvents used in the case study are listed in Table S 28. The optimal solvent group, PC/TEA/DMF, mixed at a volume ratio of 53/8/39 to form a total of 40 mL solvents, costs £2.04, whereas using only NMI costs £71.20 (Table S 29). Applying the optimized solvent mixtures reduced the cost by *ca*. 97% compared to conventional methods.

Table S 28 Prices of all solvents used in the case study.

Chemicals	Price (£/mL)
1-methylimidazole (NMI)	1.78
Triethylamine (TEA)	0.085
tert-Butyl alcohol (*t*BuOH)	0.56
N-Methyl-2-pyrrolidone (NMP)	0.1
Acetonitrile (ACN)	0.088
Dimethyl sulfoxide (DMSO)	0.26
Propylene carbonate (PC)	0.02
Dimethylformamide (DMF)	0.086

Table S 29 Prices breakdown of 40 mL 53/8/39 PC/TEA/DMF versus NMI.

	PC	TEA	DMF	Total
Volume (mL)	21.10	3.25	15.65	40.00
Price (£)	0.42	0.28	1.34	**2.04**
	NMI			
Volume (mL)	40.00			40.00
Price (£)	1.78			**71.2**

References

(1) Hansen, C. M. *Hansen Solubility Parameters: A User's Handbook*, 2nd ed.; CRC press, Boca Raton, 2007.

(2) Kim, S.; Chen, J.; Cheng, T.; Gindulyte, A.; He, J.; He, S.; Li, Q.; Shoemaker, B. A.; Thiessen, P. A.; Yu, B.; Zaslavsky, L.; Zhang, J.; Bolton, E. PubChem 2025 Update. *Nucleic Acids Res.* **2025**, *53* (D1), D1516–D1525.

https://doi.org/10.1093/nar/gkae1059.

(3) Mihalovits, M. Temperature and Pressure Effects on the Hydrogen-Bonding Hansen Solubility Parameter: Cases of n-Alkanols (C1-C5). *J. Mol. Liq.* **2022**, *363*, 119910. https://doi.org/https://doi.org/10.1016/j.molliq.2022.119910.

(4) Hansen, C. M.; Beerbower, A. Solubility Parameters. In *Kirk-Othmer Encyclopedia of Chemical Technology*; Standen, A., Ed.; Interscience New York, 1971; pp 889–910.

(5) Abott, S. *Hansen Solubility Parameters in Practice (HSPiP)*. https://hansen-solubility.com/HSPiP/ (accessed 2026-08-20).

(6) Virtanen, P.; Gommers, R.; Oliphant, T. E.; Haberland, M.; Reddy, T.; Cournapeau, D.; Burovski, E.; Peterson, P.; Weckesser, W.; Bright, J.; van der Walt, S. J.; Brett, M.; Wilson, J.; Millman, K. J.; Mayorov, N.; Nelson, A. R. J.; Jones, E.; Kern, R.; Larson, E.; Carey, C. J.; Polat, \.Ilhan; Feng, Y.; Moore, E. W.; VanderPlas, J.; Laxalde, D.; Perktold, J.; Cimrman, R.; Henriksen, I.; Quintero, E. A.; Harris, C. R.; Archibald, A. M.; Ribeiro, A. H.; Pedregosa, F.; van Mulbregt, P.; SciPy 1.0 Contributors. {SciPy} 1.0: Fundamental Algorithms for Scientific Computing in Python. *Nat. Methods* **2020**, *17*, 261–272. https://doi.org/10.1038/s41592-019-0686-2.

(7) Gómez-Bombarelli, R.; Wei, J. N.; Duvenaud, D.; Hernández-Lobato, J. M.; Sánchez-Lengeling, B.; Sheberla, D.; Aguilera-Iparraguirre, J.; Hirzel, T. D.; Adams, R. P.; Aspuru-Guzik, A. Automatic Chemical Design Using a Data-Driven Continuous Representation of Molecules. *ACS Cent. Sci.* **2018**, *4* (2), 268–276. https://doi.org/10.1021/acscentsci.7b00572.

(8) Landrum, G. RDKit: Open-Source Cheminformatics. 2024. https://doi.org/10.5281/zenodo.591637.

(9) Narzary, B. B.; Karatayeva, U.; Mintah, J.; Villeda-Hernandez, M.; Faul, C. F. J. Bifunctional Metal-Free Porous Polyimide Networks for CO2 Capture and Conversion. *Mater. Chem. Front.* **2023**, *7* (19), 4473–4481. https://doi.org/10.1039/d3qm00639e.

(10) Liao, Y.; Weber, J.; Faul, C. F. J. Fluorescent Microporous Polyimides Based on Perylene and Triazine for Highly CO2-Selective Carbon Materials. *Macromolecules* **2015**, *48* (7), 2064–2073. https://doi.org/10.1021/ma501662r.
